# Horizontal alignment of 5′ -> 3′ intergene distance segment tropy with respect to the gene as the conserved basis for DNA transcription

**DOI:** 10.4155/fsoa-2016-0070

**Published:** 2016-12-02

**Authors:** Hemant Sarin

**Affiliations:** 1 Freelance Investigator in Translational Science & Medicine (unaffiliated), 833 Carroll Road, Charleston, WV 25314, USA

**Keywords:** cell membrane pressuromodulation, eukaryote, genomics, infra-pressuromodulated gene, nuclear pressuromodulation, peptide, prokaryote, small hormone pressuromodulator, supra-pressuromodulated gene, virus

## Abstract

**Aim:**

To study the conserved basis for gene expression in comparative cell types at opposite ends of the cell pressuromodulation spectrum, the lymphatic endothelial cell and the blood microvascular capillary endothelial cell.

**Methods:**

The mechanism for gene expression is studied in terms of the 5′ -> 3′ direction paired point tropy quotients (*prpT*_Q_s) and the final 5′ -> 3′ direction episodic sub-episode block sums split-integrated weighted average-averaged gene overexpression tropy quotient (*esebssiwaagoT*_Q_).

**Results:**

The final 5′ -> 3′ *esebssiwaagoT_Q_* classifies an lymphatic endothelial cell overexpressed gene as a supra-pressuromodulated gene (*esebssiwaagoT*_Q_ ≥ 0.25 < 0.75) every time and classifies a blood microvascular capillary endothelial cell overexpressed gene every time as an infra-pressuromodulated gene (*esebssiwaagoT*_Q_ < 0.25) (100% sensitivity; 100% specificity).

**Conclusion:**

Horizontal alignment of 5′ -> 3′ intergene distance segment tropy *wrt* the gene is the basis for DNA transcription in the pressuromodulated state.

## Lay abstract

Genes are expressed in cells, however, the basis for the expression of certain genes over other genes remains poorly understood. In 2015, it was discovered that hormones bind to their cell membrane receptors to tighten the cell membrane to fine tune the pressurization of a cell, the actual signal for the expression of genes. In this science it is discovered that it can be predicted with certainty which genes will be overexpressed in cells that are more pressurized and which genes will be overexpressed in cells that are less pressurized. This novel discovery sheds light on why cells exist as certain cells in the biological system, why they remain as those cells and why cells transform into other cells such as cancer cells.

**Figure 1.**
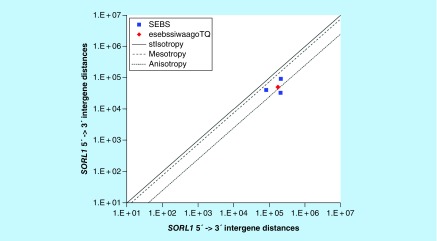
**>11,864 ≤265,005 gene base category, *SORL1*, sub-episode block sums (*MSEBS*; *ASEBS*) and the final episodic sub-episode block sums split-integrated weighted average-averaged gene overexpression tropy quotient (*esebssiwaagoT*_Q_) @ Episode 2.**

**Figure 2.**
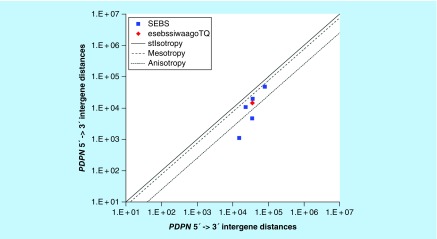
**>11,864 ≤265,005 gene base category, *PDPN*, sub-episode block sums (*MSEBS*; *ASEBS*) and the final episodic sub-episode block sums split-integrated weighted average-averaged gene overexpression tropy quotient (*esebssiwaagoT*_Q_) @ Episode 2.**

**Figure 3.**
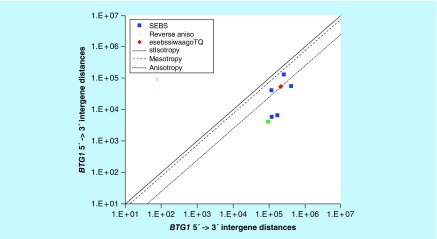
**>11,864 ≤265,005 gene base category, *BTG1*, sub-episode block sums (*MSEBS*; *ASEBS*) and the final episodic sub-episode block sums split-integrated weighted average-averaged gene overexpression tropy quotient (*esebssiwaagoT*_Q_) @ Episode 2.** Filled green square, first non-mono anisotropic point (95,217, 4,153) of the second *ASEBS* (419,487, 57,054) shown without the non-mono anisotropic point (95,217, 4,153); green star, immediately preceding 3′ -> 5′ reverse anisotropic point 78, 93,666.

**Figure 4.**
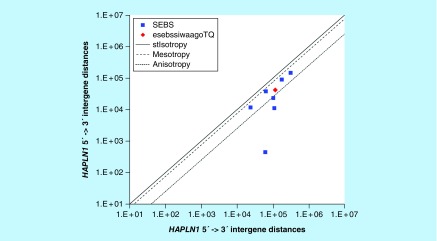
**>11,864 ≤265,005 gene base category, *HAPLN1*, sub-episode block sums (*MSEBS*; *ASEBS*) and the final episodic sub-episode block sums split-integrated weighted average-averaged gene overexpression tropy quotient (*esebssiwaagoT*_Q_) @ Episode 2.**

**Figure 5.**
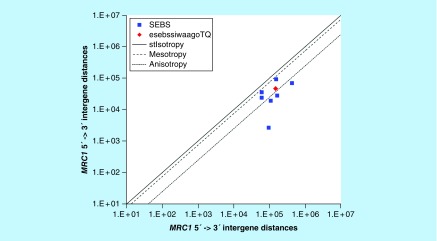
**>11,864 ≤265,005 gene base category, *MRC1*, sub-episode block sums (*MSEBS*; *ASEBS*) and the final episodic sub-episode block sums split-integrated weighted average-averaged gene overexpression tropy quotient (*esebssiwaagoT*_Q_) @ Episode 2.**

**Figure 6.**
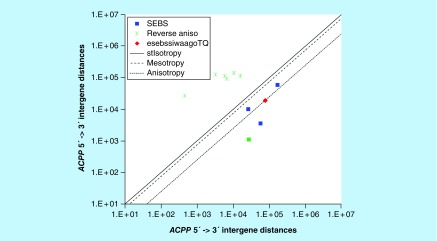
**>11,864 ≤265,005 gene base category, *ACPP*, sub-episode block sums (*MSEBS*; *ASEBS*) and the final episodic sub-episode block sum split-integrated weighted average-averaged gene overexpression tropy quotient (*esebssiwaagoT*_Q_) @ Episode 2.** Filled green square, non-mono anisotropic not considered (NC) first sub-episode block (SEB) (NC *ASEBS* 26,865, 1,099) due to six preceding 3′-> 5′ reverse anisotropic points of greater magnitude (Green stars), in which case the 0 order *prpT*_Q_ SEB (first considered SEB) is the immediately following SEB, a mesotropic SEB.

**Figure 7.**
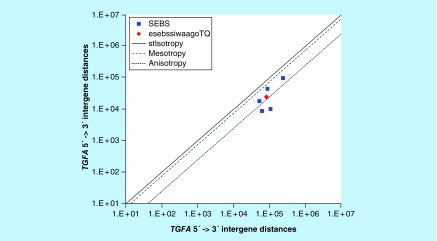
**>11,864 ≤265,005 gene base category, *TGFA*, sub-episode block sums (*MSEBS*; *ASEBS*) and the final episodic sub-episode block sum split-integrated weighted average-averaged gene overexpression tropy quotient (*esebssiwaagoT*_Q_) @ Episode 2.**

**Figure 8.**
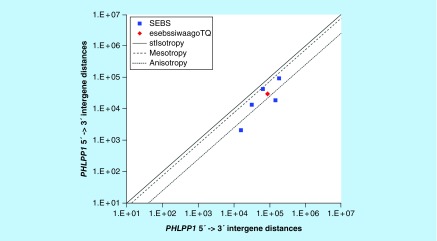
**>11,864 ≤265,005 gene base category, *PHLPP1*, sub-episode block sums (*MSEBS*; *ASEBS*) and the final episodic sub-episode block sums split-integrated weighted average-averaged gene overexpression tropy quotient (*esebssiwaagoT*_Q_) @ Episode 2.**

**Figure 9.**
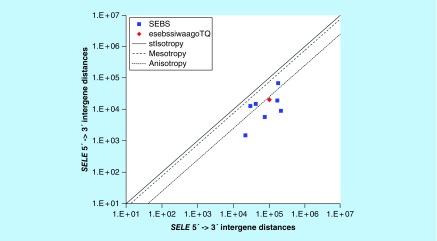
**>11,864 ≤265,005 gene base category, *SELE*, sub-episode block sums (*MSEBS*; *ASEBS*) and the final episodic sub-episode block sums split-integrated weighted average-averaged gene overexpression tropy quotient (*esebssiwaagoT*_Q_) @ Episode 2.**

**Figure 10.**
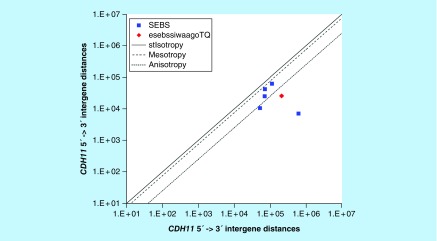
**>11,864 ≤265,005 gene base category, *CDH11*, sub-episode block sums (*MSEBS*; *ASEBS*) and the final episodic sub-episode block sums split-integrated weighted average-averaged gene overexpression tropy quotient (*esebssiwaagoT*_Q_) @ Episode 2.**

**Figure 11.**
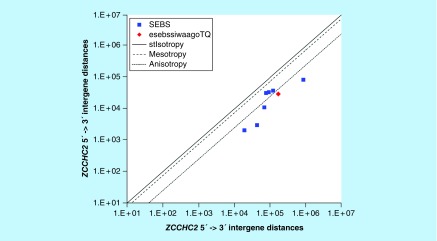
**>11,864 ≤265,005 gene base category, *ZCCHC2*, sub-episode block sums (*MSEBS*; *ASEBS*) and the final episodic sub-episode block sums split-integrated weighted average-averaged gene overexpression tropy quotient (*esebssiwaagoT*_Q_) @ Episode 2.**

**Figure 12.**
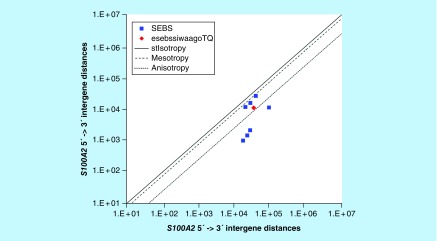
**≤11,864 gene base category, *S100A2*, sub-episode block sums (*MSEBS*; *ASEBS*) and the final episodic sub-episode block sums split-integrated weighted average-averaged gene overexpression tropy quotient (*esebssiwaagoT*_Q_) @ Episode 3.**

**Figure 13.**
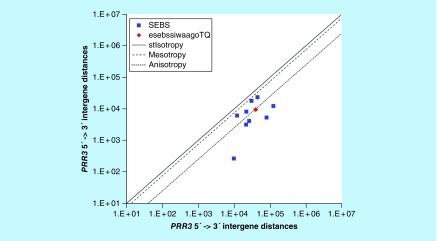
**≤11,864 gene base category, *PRR3*, sub-episode block sums (*MSEBS*; *ASEBS*) and the final episodic sub-episode block sums split-integrated weighted average-averaged gene overexpression tropy quotient (*esebssiwaagoT*_Q_) @ Episode 3.**

**Figure 14.**
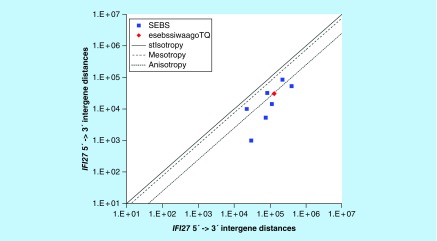
**≤11,864 gene base category, *IFI27*, sub-episode block sums (*MSEBS*; *ASEBS*) and the final episodic sub-episode block sums split-integrated weighted average-averaged gene overexpression tropy quotient (*esebssiwaagoT*_Q_) @ Episode 3.**

**Figure 15.**
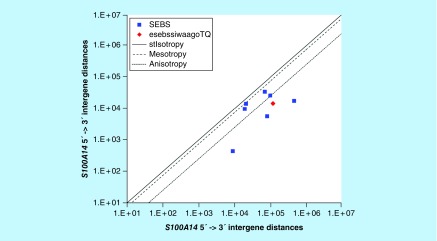
**≤11,864 gene base category, *S100A14*, sub-episode block sums (*MSEBS*; *ASEBS*) and the final episodic sub-episode block sums split-integrated weighted average-averaged gene overexpression tropy quotient (*esebssiwaagoT*_Q_) @ Episode 3.**

**Figure 16.**
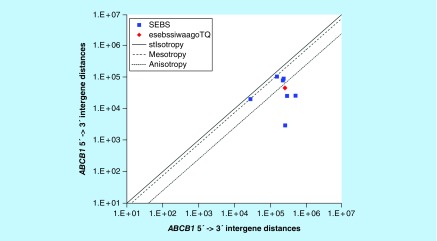
**>265,005 <607,463 gene base category, *ABCB1*, sub-episode block sums (*MSEBS*; *ASEBS*) and the final episodic sub-episode block sums split-integrated weighted average-averaged gene overexpression tropy quotient (*esebssiwaagoT*_Q_) @ Episode 4.**

**Figure 17.**
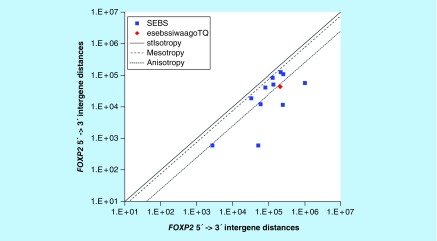
**≥607,463 <2,241,933 gene base category, *FOXP2*, sub-episode block sums (*MSEBS*; *ASEBS*) and the final episodic sub-episode block sums split-integrated weighted average-averaged gene overexpression tropy quotient (*esebssiwaagoT*_Q_) @ Episode 5.**

**Figure 18.**
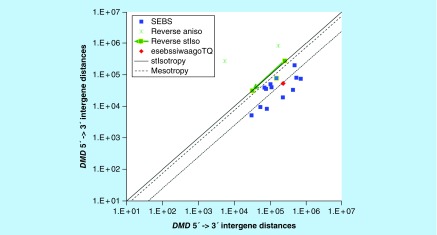
**≥2,241,933 gene base category, *DMD*, sub-episode block sums (*MSEBS*; *ASEBS*) and the final episodic sub-episode block sums split-integrated weighted average-averaged gene overexpression tropy quotient (*esebssiwaagoT*_Q_) @ Episode 6.** Filled green squares with yellow border interconnected by arrowed line, 3′ -> 5′ reverse isotropy point (253,856, 280,037) of the tempered considered (TC) first *MSEBS* upstream intergene distance 0.125-factor adjusted to 31,732, 31,732 instead of upstream intergene distance 0.25-factor adjusted to 63,464, 63,464 as there are two immediately preceding reverse anisotropy points (167,442, 830,657; 5,488, 272,663) of sufficient 3′ -> 5′ reverse anisotropy (Green stars) to diminish the 3′ -> 5′ reverse isotropy stabilizing effect of 253,856, 280,037 to 31,732, 31,732; Filled blue square with yellow border, first *MSEBS* with 0.125-factor adjusted 31,732, 31,732 point.

In the biological system in the physiologic state *in vivo*, water flux across cell membrane channels in response to changes in system osmoregulators (extracellular sodium, intracellular potassium and extracellular and intracellular glucose) maintains baseline biological system osmotic pressure and turgor; however, water flux in response to osmoregulators is not a specific regulator of intracellular pressure as it is a concomitant regulator of both extracellular and intracellular pressure of the biological system.

During the blastocyst-to-gastrula-to-neuralation developmental stages, macropressurization occurs, the mesoderm is subject to the most macropressurization (least intra-cellularly pressurized cells), the endoderm is subject to intermediate macropressurization (intermediately intracellularly pressurized cells) and the ectoderm is subject to the least macropressurization (most intracellularly pressurized cells), as a result of which the baseline densities (g/cm^3^) of the respective germ layers are set. In the case of the ectoderm, there is the development of the cerebrospinal fluid (CSF) suspended buoyant CNS tissue, which begins as the least dense tissue initially containing the most intracellularly pressurized cells that sprout extensively over long distances after which nuclear pressure decreases substantially resulting in non-dividing neuronal cells (less intracellularly pressurized cells) that further differentiate into specific neuron populations (i.e., acetylcholinergic, glutaminergic, γ-aminobutyric acid, dopaminergic, serotonergic) in response to local microenvironment growth factors [1]. This is analogous to cells *in vitro*, when cultured cells are in the proliferative phase at approximately 30% confluence {relatively greater intracellularly pressurized cells, as intracellular pressure is much greater [>>>] than extra-cellular} due to lesser cell-to-cell contact, but are in the non-proliferative phase at approximately 80% confluence {relatively lesser intracellularly pressurized cells, as intracellular pressure is only greater [>] extra-cellular} due to greater cell-to-cell contact, as it has been observed by the atomic force microscopy (AFM) that the Young's modulus (kPa) of cells decreases with increasing E-cadherin micro-bead cell contact surface area (relatively less intracellular pressure with increasing cell contact extracellular pressure) [2].

Cells grown in culture are subject only to atmospheric pressure (i.e. 760 mmHg), however the pressure that cells are subject to *in vivo* is much greater than circulatory blood pressure (i.e. 120/80 mmHg) and greater than atmospheric pressure, as true biological system pressure is the force per unit area (kPa) that cells are actually subject to *in vivo*, as there is pulsatile pressure through inter-endothelial or inter-epithelial junction open cross-sectional surface area in the pressurized biological system *in vivo*. Support of this supposition comes from two observations of cell macropressurization at extreme ends of the cell macropressurization spectrum:

The least, when under atmospheric pressure decreasing underlying substrate stiffness (decreasing stiffness of gel substrate by decreasing cross-linking) [3–5] results in decreased cell proliferation [6] as overall extracellular pressure (atmospheric pressure and substrate pressure) decreases below that of the lowest level of biologically possible macropressurization, which can only be rescued by growth factors [7]; as opposed to
The greatest, when stiff microcapsule-encapsulation [8] or *in situ* application of neoplastic-level stiff intra-ductal pressure to isolated acini [9] results in intimately apposed-and-juxtaposed cell membrane stiffness, actually increases intracellular pressure [10] and results in cell proliferation [8].

These observations taken together imply that:

In the pressurized biological state *in vivo*, normal attached tissue cells are relatively less pressurized cells in comparison to normal free moving circulatory cells that are relatively more pressurized cells (i.e., tri-lobed nucleus neutrophils > bi-lobed nucleus eosinophils > mono-lobed nucleus cells, among others); and that
In the pressurized biological state *in vivo*, there are relative decreases in the effectiveness of growth factors, particularly in the effectiveness of lesser potency growth factors [11].

Building on these observations, it has been recently described that cell membrane pressuromodulation, defined as alterations in cell compliance in response cell membrane pressuromodulators {Δ*P* [mmHg]/Δ*V* [cm^3^]}, where the change in cell volume Δ*V* (Δ cm^3^) is miniscule (∼constant) as compared with the change in intracellular pressure (*Δ*P), *P*postpressuromodulator – *P*pre-pressuromodulator (Δ mmHg), whereby alterations in cell compliance in response to cell membrane pressuromodulators could by assessed *vis a vis* the Young's modulus {*F*orce/*A*rea [kPa]/Δ*L*ength/*L*ength initial (ratio); kPa} [12], as the Young's modulus is a measure of cell membrane compliance and would serve as a surrogate measure of changes in cell compliance itself. Cell membrane pressuromodulation plays the pivotal role in the specific regulation of cellular and nuclear function [13–15], via:

Direct cell membrane pressuromodulator without oxidative stress-mediated decrease in cell membrane compliance and increase in intracellular pressure, which favors cell differentiation toward pluripotency, and cell division or cell mitogenic multi-nucleation, for example, as is the case for aldosterone (number of mineralocorticoid receptors: 169 per cell; *K*_D_ = 0.52 × 10^-10^ with *t*_1/2_ @ receptor: 140 min) [16–20], for dihydrotestosterone/testosterone (< number of receptors) [21–23], for 17β-estradiol [24,25], for TGF-β1 [22], for HGF/SF [26,27], for IL-1α/β [25,28], for EGF [25], for bFGF [29], for PTH/PTHrP [27], for VEGF (2+ endocytic) [30,31], for phorbol of 12-myristate 13-acetate (PMA, TPA; hydroxylo-, carbonylo- endocytic)[25,32–33], and for dynamic stress/strain [34]; via
Direct cell membrane pressuromodulator with oxidative stress-mediated increase in cell membrane compliance and decrease in intracellular pressure, which favors cell differentiation away from pluripotency, for example, as is the case for dexamethasone (number of glucocorticoid receptors: 1322 per cell; *K*_D_ = 3.7 × 10^-9^ with *t*_1/2_ @ receptor: 100 min; > number of receptor-mediated oxidative stress) [16–17,28,30], for dihydrotestosterone/testosterone (> number of receptors; > number of receptor-mediated oxidative stress) [35], and for GM-CSF/CSF-1 (receptor-mediated oxidative stress) [36–38]); and via
Indirect cell membrane pressuromodulator with bilayer cholesterol removal without oxidative stress-mediated decrease in cell membrane compliance and increase in intracellular pressure, which favors cell differentiation toward pluripotency, and cell division or cell mitogenic multi-nucleation, for example, as is the case for ketoconazole [39];
Indirect cell membrane pressuromodulator with esterase activity-related oxidative stress-mediated increase in cell membrane compliance and decrease in intracellular pressure, which favors cell differentiation away from pluripotency, for example, as is the case for 12-myristate and 13-acetate of phorbol 12-myristate 13-acetate (PMA, TPA) [30,33,40]; and
Indirect cell membrane pressuromodulator with bilayer pertubation-mediated increase in cell membrane compliance and decrease in intracellular pressure, which also favors cell differentiation away from pluripotency, for example, as is the case for tocopherols [41,42], for calcifidiol [41,43], and for retinoic acid [43,44].

Even as the various forms of cell membrane pressuromodulation have been shown to be important in the regulation of cellular and nuclear function, an aspect that remains poorly understood is the conserved basis for cellular pressuromodulation state-dependent DNA transcription, which can be understood based on knowledge of the following four knowns for DNA transcription:

The direction of RNA polymerase-dependent DNA transcription is 5′ -> 3′ for both helix (+) and (-) strand transcription;
Genes are transcribable series of bases with a pre-weighted 5′ proximal promoter sequence constitutively bound by certain transcription factors to which additional adapter transcription factors associate on-induction via hydrophobic core interaction [33];
Non-gene intergene segments are non-transcribable promoter-less series of bases with base-associated nuclear protein hydrophobic cores, where shorter intergene segment distances constitute lesser weighted intergene distances; and
In the cases of both bullet points (2) and (3), the anionic phosphodiester moieties associate only loosely with nucleosome histone cationic lysine R-groups [45–47].

Based on these four knowns, it can be postulated with a reason degree of certainty that the necessary prerequisite for gene transcription is a cellular pressuromodulation-dependent establishment of a horizontal 5′ -> 3′ reading frame of the most asymmetrically weighted 5′ -> 3′ anisotropic intergene segment pairs with respect to the gene (*wrt* gene) and the lesser asymmetrically weighted 5′ -> 3′ mesotropic intergene segment pairs (*wrt* gene), while the symmetrically weighted 3′ -> 5′ and 5′ -> 3′ isotropic intergene segment pairs (*wrt* gene) remain horizontal and function as stabilizing intergene segment pairs.

Furthermore, it can be postulated with a reason degree of certainty that the conserved basis for DNA transcription and replicative gene overexpression progression to mitogenic multi-nucleation is associated with decreased cell membrane compliance primarily related to increased endocytic cell membrane pressuromodulation, which results in mitogenic multi-nucleation, for example:

In the case of the multi-nucleated giant cell [48,49] arising from the part-anchored mono-nucleated CD68^+^/CD163^+^ M2 macrophage [37,50];
In the case of the multi-nucleated (enlarged) osteoclast [51] from the part-anchored mono-nucleated TRAP+/DC-STAMP+ osteoclast [52]; and
In the case of the multi-nucleated sprouted diaphragm fenestrated lymphatic capillary endothelial cell (LEnC) [53–56] from the anchored mono-nucleated lymphatic capillary endothelial cell [53].

These cell types all proceed directly to mitogenesis multi-nucleation without preceding cell division, in the case of the multi-nucleated giant cell, due to endocytosis-episodic burst endocytosis of high-molecular-weight debris [48,49]/collagen V via episodic burst overexpression of uPARAP (Endo180; uTPAR; MRC2) [57–59]; in the case of the multi-nucleated osteoclast, due to endocytosis-episodic burst endocytosis of degraded collagen I [60,61] via episodic burst overexpression of OSCAR [51,62]; and in the case of the multi-nucleated LEnC, due to endocytosis-burst endocytosis vesiculo-vacuolo-exosomalization of cell membrane [63] via episodic burst overexpression of VEGFR2 (KDR/Flk-1) [64]. Therefore, the resultant cellular pressuromodulation of such multi-nucleated cell types is a more sustained level of greater pressuromodulation as compared with cell types that undergo mitogenesis immediately followed by cell division, which results in a significant decrease in cellular cum nuclear pressurization. As such, mitogenic without division multi-nucleated cell types can be considered model cell types to study the basis for gene overexpression in over-pressuromodulated cells as compared with the basis for gene overexpression in under-pressuromodulated non-mitogenic cells, for example, the blood microvascular capillary endothelial cell (BMEnC), which is much less pressuromodulated compared with the LEnC.

In this research study, the conserved basis for gene overexpression is studied in comparative cell types at opposing ends of pressuromodulation set point spectrum, the LEnC representing the over-pressuromodulated cell type and BMEnC representing the under-pressuromodulated cell type utilizing a published open access cDNA micro-array mRNA expression dataset [65]. The conserved basis for gene overexpression is understood in terms of the paired point tropy quotients (*prpT*_Q_s) and the 5′ -> 3′ direction episodic sub-episode block sums split-integrated weighted average-averaged gene overexpression tropy quotients (*esebssiwaagoT*_Q_s).

## Methods

### Data acquisition

Seven sets of most differentially overexpressed LEnC and BMEnC genes at the greater than non-adjusted twofold level and two sets of juxtaposed lesser differentially overexpressed LEnC and BMEnC genes between the non-adjusted one- to two-fold level were selected from a published open access dataset of microarray mRNA expression levels [65] (Supplementary file 1 – Supplementary Table S1). For these 18 genes, all of the transcribed loci base locations, both protein coding and noncoding, were mined utilizing the GeneCards [66] genomic neighborhood GeneLoc genome locator database [67] and the LNCipedia.org database [68].

### Determination of the 3′ -> 5′ & 5′ - >3′ direction *prpT*_Q_s

Non-transcribing intergene distances were determined upstream and downstream from the gene of interest. Then, the paired point tropy quotients (*prpT*_Q_; fract) for the polymerase non-transcribing reverse 3′ -> 5′ direction (Equation 1) were determined, and the *prpT*_Q_s for the polymerase transcribing 5′ -> 3′ direction wherein the 0^th^ order *prpT*_Q_ is the first intergene distance pair *prpT*_Q_ (Equation 2) were determined, as follows (Supplementary file 2 – Supplementary Table S2):Equation 1
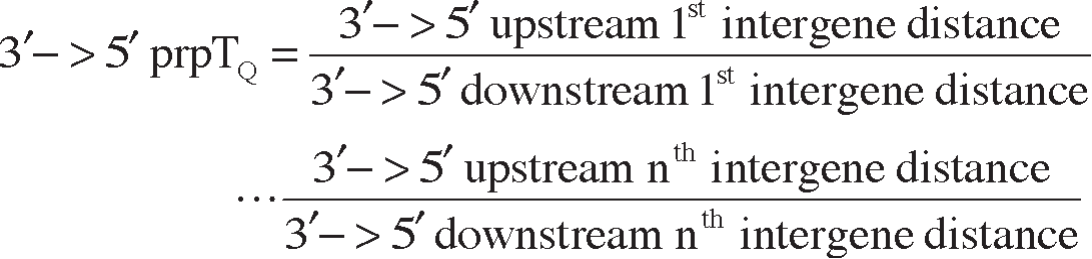

Equation 2
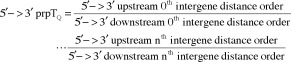



where the total number of *prpT*_Q_s is the *n* which achieves the *n* ^th^ order of 5′-> 3′ *prpT*_Q_s to either 2, 3, 4, 5 or 6 episodes.

### Determination of anistropic & mesotropic sub-episode blocks for characterization of episodicity

The anisotropic and mesotropic sub-episode blocks {SEBs; anisotropic sub-episode block [ASEB], mesotropic sub-episode block [MSEB]} were determined, as follows:

Where an SEB is one with either a single, dual, triple or multiple series of *prpT*_Q_s;
Where the 0^th^ order *prpT*_Q_ SEB is the first 5′ -> 3′ *prpT*_Q_ SEB;
Where an ASEB is one with one *prpT*_Q_, two *prpT*_Q_s, three *prpT*_Q_s or multiple *prpT*_Q_s of < 0.25 each;
Where the 0^th^ order first 5′ -> 3′ *prpT*_Q_ ASEB is a *non-anisotropic SEB* (not considered [NC]) when it is preceded by reverse anisotropy 3′ -> 5′ *prpT*_Q_s of equivalent or greater magnitude;
Where an MSEB is one with one *prpT*_Q_, two *prpT*_Q_s, three *prpT*_Q_s or multiple *prpT*_Q_s of ≥ 0.25 < 0.75 each.

An episode was then defined, as follows:

Where one episode is a single anisotropic *prpT*_Q_(s) sub-episode block (ASEB) followed by a single mesotropic *prpT*_Q_(s) sub-episode block (MSEB), *or vice versa* {i.e., beginning or ending with an ASEB [anisotropic period], beginning or ending with a MSEB [mesotropic period]}, with overlap between the ASEB and the MSEB periods;
Where a stabilizing isotropy (stIsotropy) intergene distance pair is an almost horizontal 5′ -> 3′ or 3′ -> 5′ intergene distance pair that has a *prpT*_Q_ ≥ 0.75 (∼0 slope point) and is always considered to be the immediately preceding stabilizing intergene distance pair for an immediately proceeding SEB, either an ASEB *prpT*_Q_ intergene distance pair or a MSEB *prpT*_Q_ intergene distance pair;
Where instances of stIsotropy *prpT*_Q_ points within an SEB are only considered after determination of the number of initial episodes for categorizing gene (either as an Episode 2, 3, 4, 5 or 6 category gene);
Where the final number of SEBs for a gene category is the number of SEBs following consideration of stIsotropy *prpT*_Q_ points {5′ -> 3′ direction and 3′ -> 5′ direction [*prpT*_Q_ ≥ 0.75]};
Where an immediately preceding factor-adjusted stIsotropy *prpT*_Q_ point intergene distance pair (or one within an SEB) is summed with the immediately proceeding SEB *prpT*_Q_ point intergene distance pair, which may result in an ASEB-to-MSEB conversion or an MSEB-to-stIsotropy conversion (i.e., of a single anisotropic *prpT*_Q_ point ASEB or single mesotropic *prpT*_Q_ point MSEB), and would result in an initial SEB count +/- 2 interconversion (i.e., 5 SEB -> 7 SEB; 5 SEB -> 3 SEB).

### Determination of the 5′ -> 3′ direction episodic sub-episode block sums split-integrated weighted average-averaged gene overexpression tropy quotients (*esebssiwaagoT*_Q_s) to the final *esebssiwaagoT*_Q_

The complete 5′ -> 3′ direction episodic sub-episode block sums split-integrated weighted average-averaged gene overexpression tropy quotients (*esebssiwaagoT*_Q_s; fract) were determined to the final *esebssiwaagoT*_Q_ in upstream anisotropic, upstream mesotropic, downstream anisotropic and downstream mesotropic parts.

First, the upstream part anisotropic sub-episode block sum (*uppASEBS*), the upstream part mesotropic sub-episode block sum (*uppMSEBS*), the downstream part ASEBS (*dppASEBS*) and the downstream part mesotropic sub-episode block sum (*dppMSEBS*) were determined.

The 5′ -> 3′ *uppASEBS* adjusted for *uppASEBS 5′ -> 3′ stabilizing isotropy* (*stIsotropy*) (Equation 3a), 5’ -> 3′ *uppMSEBS* adjusted for *uppMSEBS 5′ -> 3′ stIsotropy* (Equation 3b), 5′ -> 3′ *dppASEBS* adjusted for *5′ -> 3′ dppASEBS stIsotropy* (Equation 3c), and 5′ -> 3′ *dppMSEBS* adjusted for *dppMSEBS 5′ -> 3′ stIsotropy* (Equation 3d), as follows:Equation 3a
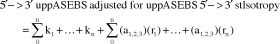

Equation 3b
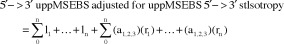

Equation 3c
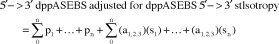

Equation 3d
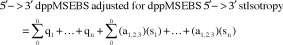

Where k is an upstream 5′ -> 3′ direction intergene segment distance point in an ASEB;
Where l is an upstream 5′ -> 3′ direction intergene segment distance point in an MSEB;
Where p is a downstream 5′ -> 3′ direction intergene segment distance point in an ASEB;
Where q a downstream 5′ -> 3′ direction intergene segment distance point in an MSEB;
Where r is the upstream 5′ -> 3′ direction intergene segment distance stIsotropy point in an ASEB or in an MSEB (r_n_ for an ASEB or MSEB with more than one stIsotropy point);
Where s is the downstream 5′ -> 3′ direction intergene segment distance stIsotropy point in an ASEB or in an MSEB (s_n_ for an ASEB or MSEB with more than one stIsotropy point);
Where a is a_1_ = 0 for no preceding 5′ -> 3′ or 3′ -> 5′ stIsotropy or for preceding 5′ -> 3′ or 3′ -> 5′ stIsotropy more than (>) 5 intergene distance pairs away;
Where a is a_2_ = 0.125 for preceding 5′ -> 3′ or 3′ -> 5′ stIsotropy in the presence of preceding intervening 3′ -> 5′ reverse anisotropy less than or equal to (≤) 5 intergene distance pairs away;
Where a is a_3_ = 0.25 for immediately preceding 5′ -> 3′ or 3′ -> 5′ stIsotropy in the absence of intervening 3′ -> 5′ reverse anisotropy

The 5′ -> 3′ *uppASEBS* adjusted for *uppASEBS 3′ -> 5′ stabilizing isotropy* (*stIsotropy*) (Equation 3e), 5′ -> 3′ *uppMSEBS* adjusted for *uppMSEBS 3′ -> 5′ stIsotropy* (Equation 3f), 5′ -> 3′ *dppASEBS* adjusted for *dppASEBS 3′ -> 5′ stIsotropy* (Equation 3g) and the 5′ -> 3′ *dppMSEBS* adjusted for *dppMSEBS 3′ -> 5′ stIsotropy* were determined (Equation 3h), as follows:Equation 3e
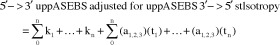

Equation 3f
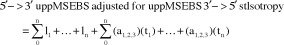

Equation 3g
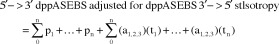

Equation 3h
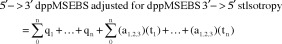

Where t is the upstream 3′ -> 5′ direction intergene segment distance stIsotropy point in an ASEB or in an MSEB (t_n_ for an ASEB or MSEB with more than one stIsotropy point);
Where t is *also* used as the downstream 3′ -> 5′ direction intergene segment distance stIsotropy point in an ASEB or in an MSEB (t_n_ for an ASEB or MSEB with more than one stIsotropy point).

Second, the upstream part ASEB sums (*uppASEBS*) split-integrated weighted average (*uppasebssiwa*) (Equation 4a), the upstream part MSEB sums (*uppMSEBS*) split-integrated weighted average (*uppmsebssiwa*) (Equation 4b), the downstream part ASEB sums (*dppASEBS*) split-integrated average (*dppasebssiwa*) (Equation 4c), and the downstream part MSEB sums (*dppMSEBS*) split-integrated weighted average (*dppmsebssiwa*) (Equation 4d) were determined, as follows:Equation 4a
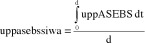

Equation 4b
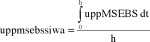

Equation 4c
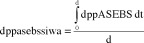

Equation 4d
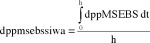

Where d is the number of integrated upstream part anisotropic sub-episode block sums (*uppASEBS*) and the number of integrated downstream part anisotropic sub-episode block sums (*dppASEBS*);
Where h is the number of integrated upstream part mesotropic sub-episode block sums (*uppMSEBS*) and the number of integrated downstream part mesotropic sub-episode block sums (*dppMSEBS*).

Third, the average of the *uppasebssiwa* and the *uppmsebssiwa* (*uppesebssiwaa*) (Equation 5a), and the average of the *dppasebssiwa* and the *dppmsebssiwa* (*dppesebssiwaa*) (Equation 5b) were determined, as follows:Equation 5a
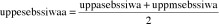

Equation 5b
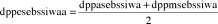



Fourth, the complete episodic sub-episode block sums split-integrated weighted average-averaged gene overexpression tropy quotients (*esebssiwaagoT*_Q_s) (Equation 6) were determined to the final complete *esebssiwaagoT*_Q_, as follows:Equation 6
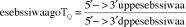

Where the *esebssiwaagoT*_Q_ at Episode 2 is the final *esebssiwaagoT*_Q_ for genes > 11,864 ≤ 265,005 bases;
Where the *esebssiwaagoT*_Q_ at Episode 3 is the final *esebssiwaagoT*_Q_ for genes ≤ 11,864 bases;
Where the *esebssiwaagoT*_Q_ at Episode 4 is the final *esebssiwaagoT*_Q_ for genes > 265,005 < 607,463 bases;
Where the *esebssiwaagoT*_Q_ at Episode 5 is the final *esebssiwaagoT*_Q_ for genes ≥ 607,463 < 2,241,933 bases;
Where the *esebssiwaagoT*_Q_ at Episode 6 is the final *esebssiwaagoT*_Q_ for genes ≥ 2,241,933 bases.

Fifth, genes were determined to be either infra-pressuromodulated or supra-pressuromodulated, as follows:

Where a gene with an anisotropic final *esebssiwaagoT*_Q_ for genes < 0.25 is a infra-pressuromodulated gene (Infra gene);
Where a gene with a mesotropic final *esebssiwaagoT*_Q_ for genes ≥ 0.25 < 0.75 is a supra-pressuromodulated gene (Supra gene).

### Plotting of sub-episode block sum (*ASEBS*, *MSEBS*) & final *esebssiwaagoT_Q_* data

The 5′ -> 3′ downstream part *ASEBS* (*dppASEBS*) (x-axis) and the 5′ -> 3′ upstream part *ASEBS* (*uppASEBS*) (y-axis) point data; the 5′ -> 3′ downstream part *MSEBS* (*dppMSEBS*) (x-axis) and the 5′ -> 3′ upstream part *MSEBS* (*uppMSEBS*) (y-axis) point data; and the final 5′ -> 3′ downstream part episodic sub-episode block sums split-integrated weighted average-average (*dppesebssiwaa*) (x-axis) and the final 5′ -> 3′ upstream part episodic sub-episode block sums split-integrated weighted average-average (u*ppesebssiwaa*) (y-axis) point data were *log* plotted as the final complete episodic sub-episode block sums split-integrated weighted average-averaged gene overexpression tropy quotient (final *esebssiwaagoT*_Q_). In cases where there was preceding 3′ -> 5′ reverse anisotropy equivalent or greater in magnitude the reverse anisotropy points were also plotted (upstream part, x-axis; downstream part, y-axis).

## Results

### >11,864 ≤ 265,005 gene base category, *SORL1*

For *SORL1*, the beginning 5′ -> 3′ episodic character is dual mesotropy followed by dual anisotropy (B); the middle 5′ -> 3′ episodic character is dual mesotropy (M), stabilizing isotropy stabilized mono mesotropy followed by reverse stabilizing isotropy converted mono mesotropy-to-stabilizing isotropy and stabilized converted mono anisotropy-to-mesotropy (M); and the ending 5′ -> 3′ episodic character is multi mesotropy (E). For *SORL1*, the middle 3′ -> 5′ episodic character is mesotropy reverse stabilizing converting isotropy (M). *SORL1* is a (5[-2]: 3) SEB Episode 2 gene (Table 1).

For *SORL1*, there are two final MSEBs and there is one final ASEB (Figure 1). For *SORL1*, the integrated *uppmsebssiwa* is 65,960 at Episode 2 (h = 2) and the integrated *uppasebssiwa* is 33,201 at Episode 2 (d = 1); and the integrated *dppmsebssiwa* is 144,058 at Episode 2 (h = 2) and the integrated *dppasebssiwa* is 203,780 at Episode 2 (d = 1). For *SORL1*, the *uppesebssiwaa* is 49,581 and the *dppesebssiwaa* is 173,919 that results in an *esebssiwaagoT*_Q_ of 0.29 at Episode 2. *SORL1* meets the threshold of ≥ 0.25 < 0.75 for a supra-pressuromodulated gene (Table 2 & Supplementary file 3 – Supplementary Table S3).

### >11,864 ≤265,005 gene base category, *PDPN*

For *PDPN*, the beginning 5′ -> 3′ episodic character is reverse stabilizing isotropy converted mono mesotropy-to-stabilizing isotropy and stabilized mono mesotropy (B), mono mesotropy (B) followed by mono anisotropy (B); the middle 5′ -> 3′ episodic character is tri mesotropy (M), stabilizing isotropy and reverse stabilizing isotropy stabilized mono mesotropy (M), stabilizing isotropy stabilized mono mesotropy (M) followed by mono anisotropy (M); and the ending 5′ -> 3′ episodic character is reverse stabilizing isotropy stabilized mono mesotropy (E). For *PDPN*, the beginning 3′ -> 5′ episodic character is mesotropy reverse stabilizing converting isotropy (B); the middle 3′ -> 5′ episodic character is mesotropy reverse stabilizing isotropy (M); and the ending 3′ -> 5′ episodic character is mesotropy reverse stabilizing isotropy (E). *PDPN* is a (5) SEB Episode 2 gene (Table 1).

For *PDPN*, there are three final MSEBs and there are two final ASEBs (Figure 2). For *PDPN*, the integrated *uppmsebssiwa* is 25,875 at Episode 2 (h = 3) and the integrated *uppasebssiwa* at Episode 2 is 2867 (d = 2); and the integrated *dppmsebssiwa* is 45,697 at Episode 2 (h = 3) and the integrated *dppasebssiwa* is 24,905 at Episode 2 (d = 2). For PDPN, the *uppesebssiwaa* is 14,371 and the *dppesebssiwaa* is 35,301 that results in an *esebssiwaagoT*_Q_ of 0.41 at Episode 2. *PDPN* meets the threshold of ≥ 0.25 < 0.75 for a supra-pressuromodulated gene (Table 2 & Supplementary file 3 – Supplementary Table S3).

### >11,864 ≤265,005 gene base category, *BTG1*

For *BTG1*, the beginning 5′ -> 3′ episodic character is tri anisotropy (B), tri mesotropy (B), reverse stabilizing isotropy stabilized mono mesotropy (B) followed by mono mesotropy (B); the middle 5′ -> 3′ episodic character is not considered (NC) non-mono anisotropy (M), stabilizing isotropy stabilized mono anisotropy (M), mono anisotropy (M) followed by mono mesotropy (M); and the ending 5′ -> 3′ episodic character is reverse stabilizing isotropy stabilized mono anisotropy (E) followed by dual anisotropy (E). For *BTG1*, the beginning 3′ -> 5′ episodic character is mesotropy reverse stabilizing isotropy (B), the middle 3′ -> 5′ episodic character is reverse anisotropy (M); and the ending 3′ -> 5′ episodic character is anisotropy reverse stabilizing isotropy (E). *BTG1* is a (5) SEB Episode 2 gene (Table 1).

For *BTG1*, there are two final MSEBs (first *MSEBS* 259,332, 131,445; second *MSEBS* 118,464, 42,123), and there are three final ASEBs (first *ASEBS* 120,346, 5,971; second *ASEBS* 419,487, 57,054; third *ASEBS* 171,058, 6,711). There is the first non-mono anisotropic point (95,217, 4,153) of the second *ASEBS* (419,487, 57,054) that is not considered (NC) as there is an immediately preceding 3′ -> 5′ reverse anisotropic point of equivalent magnitude (79, 93,667) (Figure 3). For *BTG1*, the integrated *uppmsebssiwa* is 84,939 at Episode 2 (h = 2) and the integrated *uppasebssiwa* at Episode 2 is 23,241 (d = 3); and the integrated *dppmsebssiwa* at Episode 2 is 188,894 (h = 2) and the integrated *dppasebssiwa* at Episode 2 is 235,679 (d = 3). For *BTG1*, the *uppesebssiwaa* is 54,090 and the *dppesebssiwaa* is 212,287 that results in an *esebssiwaagoT*_Q_ of 0.25 at Episode 2. *BTG1* meets the threshold of ≥ 0.25 < 0.75 for a supra-pressuromodulated gene (Table 2 & Supplementary file 3 – Supplementary Table S3).

### > 11,864 ≤ 265,005 gene base category, *HAPLN1*

For *HAPLN1*, the beginning 5′ -> 3′ episodic character is dual mesotropy (B), mono anisotropy (B), stabilizing isotropy converted mono anisotropy-to-mesotropy (B) followed by stabilizing isotropy stabilized mono anisotropy (B); the middle 5′ -> 3′ episodic character is mono mesotropy (M), reverse stabilizing isotropy stabilized mono mesotropy (M), mono mesotropy (M) followed by mono anisotropy (M); and the ending 5′ -> 3′ episodic character is mono mesotropy (E). For *HALPN1*, the middle 3′ -> 5′ episodic character is mesotropy reverse stabilizing isotropy (M). *HAPLN1* is a [5(+2): 7] SEB Episode 2 gene (Table 1).

For *HAPLN1*, there are four final MSEBs and there are three final ASEBs (Figure 4). For *HAPLN1*, the integrated *uppmsebssiwa* is 71,228 at Episode 2 (h = 4) and the integrated *uppasebssiwa* is 11,635 at Episode 2 (d = 3); and the integrated *dppmsebssiwa* is 144,030 at Episode 2 (h = 4) and the integrated *dppasebssiwa* is 90,544 at Episode 2 (d = 3). For *HAPLN1*, the *uppesebssiwaa* is 41,431 and the *dppesebssiwaa* is 117,287 that results in an *esebssiwaagoT*_Q_ of 0.35 at Episode 2. *HAPLN1* meets the threshold of ≥ 0.25 < 0.75 for a supra-pressuromodulated gene (Table 2 & Supplementary file 3 – Supplementary Table S3).

### >11,864 ≤265,005 gene base category, *MRC1*

For *MRC1*, the beginning 5′ -> 3′ episodic character is multi (6) anisotropy [B], stabilizing isotropy and reverse stabilizing isotropy converted mono anisotropy-to-mesotropy (B), mono anisotropy (B), stabilizing isotropy stabilized mono mesotropy (B) followed by stabilizing isotropy stabilized mono mesotropy (B); the middle 5′ -> 3′ episodic character is dual anisotropy (M) followed by dual mesotropy (M); and the ending 5′ -> 3′ episodic character is multi (4) anisotropy (E). For *MRC1*, the beginning 3′ -> 5′ episodic character is anisotropy reverse stabilizing converting isotropy (B). *MRC1* is a (5 [+2]: 7) SEB Episode 2 gene (Table 1).

For *MRC1*, there are 3 final MSEBs and there are 4 final ASEBs (Figure 5). For *MRC1*, the integrated *uppmsebssiwa* is 50,393 at Episode 2 (h = 3) and the integrated *uppasebssiwa* is 29,632 at Episode 2 (d = 4); and the integrated *dppmsebssiwa* is 91,136 at Episode 2 (h = 3) and the integrated *dppasebssiwa* is 198,095 at Episode 2 (d = 4). For *MRC1*, the *uppesebssiwaa* is 46,390 and the *dppesebssiwaa* is 148,945 that results in an *esebssiwaagoT*_Q_ of 0.28 at Episode 2. *MRC1* meets the threshold of ≥ 0.25 < 0.75 for a supra-pressuromodulated gene (Table 2 & Supplementary file 3 – Supplementary Table S3).

### >11,864 ≤265,005 gene base category, *ACPP*

For *ACPP*, the beginning 5′ -> 3′ episodic character is NC nonmono anisotropy (B), mono mesotropy deviation from constancy (B) followed by mono anisotropy (B); the middle 5′ -> 3′ episodic character is mono mesotropy deviation from constancy (M) followed by stabilizing isotropy and reverse stabilizing isotropy converted mono anisotropy-to-mesotropy deviation from constancy (M); and the ending 5′ -> 3′ episodic character is mono mesotropy deviation from constancy (E). For *ACPP*, the beginning 3′ -> 5′ episodic character is reverse anisotropy (B). *ACPP* is a (5 [-2]: 3) SEB Episode 2 gene (Table 1).

For *ACPP*, of the considered SEBs, there are two final MSEBs (first *MSEBS* 26,298, 10,139; second *MSEBS* 167,150, 58,743), and there is one final mono-ASEB (first and only *ASEBS* 56,614, 3601). The non-mono-anisotropic not considered (NC) SEB (NC *ASEBS* 26,865, 1099) is the 0 order SEB as there are a series of six preceding 3′ -> 5′ reverse anisotropic points of greater magnitude (Figure 6). For *ACPP*, the integrated *uppmsebssiwa* is 34,438 at Episode 2 (h = 2) and the integrated *uppasebssiwa* is 3600 at Episode 2 (d = 1); and the integrated *dppmsebssiwa* is 96,721 at Episode 2 (h = 2) and the integrated *dppasebssiwa* is 56,613 at Episode 2 (d = 1). For *ACPP*, the *uppesebssiwaa* is 19,019 and the *dppesebssiwaa* is 76,667 that results in an *esebssiwaagoT*_Q_ of 0.25 at Episode 2. *ACPP* meets the threshold of ≥ 0.25 < 0.75 for a supra-pressuromodulated gene (Table 2 & Supplementary file 3 – Supplementary Table S3).

### >11,864 ≤265,005 gene base category, *TGFA*

For *TGFA*, the beginning 5′ -> 3′ episodic character is stabilizing isotropy stabilized mono mesotropy (B), dual mesotropy (B), mono anisotropy (B), reverse stabilizing isotropy stabilized mono anisotropy (B) followed by mono anisotropy (B); the middle 5′ -> 3′ episodic character is mono mesotropy (M), reverse stabilizing isotropy stabilized mono mesotropy (M), stabilizing isotropy and reverse stabilizing isotropy converted mono anisotropy-to-mesotropy (M) followed by mono anisotropy (M); and the ending 5′ -> 3′ episodic character is tri mesotropy (E). For *TGFA*, the beginning 3′ -> 5′ episodic character is anisotropy reverse stabilizing isotropy (B); and the middle 3′ -> 5′ episodic character is mesotropy reverse stabilizing isotropy (M) and anisotropy reverse stabilizing converting isotropy (M). *TGFA* is a (5) SEB Episode 2 gene (Table 1).

For *TGFA*, there are three final MSEBs and there are two final ASEBs (Figure 7). For *TGFA*, the integrated *uppmsebssiwa* is 53,114 at Episode 2 (h = 3) and the integrated *uppasebssiwa* is 9528 at Episode 2 (d = 2); and the integrated *dppmsebssiwa* is 124,663 at Episode 2 (h = 3) and the integrated *dppasebssiwa* is 84,199 at Episode 2 (d = 2). For TGFA, the *uppesebssiwaa* is 24,929 and the *dppesebssiwaa* is 81,129 that results in an *esebssiwaagoT*_Q_ of 0.31 at Episode 2. TGFA meets the threshold of ≥ 0.25 < 0.75 for a supra-pressuromodulated gene (Table 2 & Supplementary file 3 – Supplementary Table S3).

### >11,864 ≤265,005 gene base category, *PHLPP1*

For *PHLPP1*, the beginning 5′ -> 3′ episodic character is tri mesotropy (B) followed by mono anisotropy (B); the middle 5′ -> 3′ episodic character is stabilizing isotropy and reverse stabilizing isotropy converted mono mesotropy-to stabilizing isotropy and stabilized mono mesotropy (M), stabilizing isotropy stabilized mono anisotropy (M), mono anisotropy (M), mono mesotropy (M) followed by mono anisotropy (M); and the ending 5′ -> 3′ episodic character is dual mesotropy (E). For *PHLPP1*, the middle 3′ -> 5′ episodic character is mesotropy reverse stabilizing converting isotropy (M). *PHLPP1* is a (5) SEB Episode 2 gene (Table 1).

For *PHLPP1*, there are three final MSEBs and there are two final ASEBs (Figure 8). For *PHLPP1*, the integrated *uppmsebssiwa* is 49,476 at Episode 2 (h = 3) and the integrated *uppasebssiwa* is 10,343 at Episode 2 (d = 2); and the integrated *dppmsebssiwa* is 92,687 at Episode 2 (h = 3) and the integrated *dppasebssiwa* is 80,268 at Episode 2 (d = 2). For *PHLPP1*, the *uppesebssiwaa* is 29,910 and the *dppesebssiwaa* is 86,477 that results in an *esebssiwaagoT*_Q_ of 0.35 at Episode 2. *PHLPP1* meets the threshold of ≥ 0.25 < 0.75 for a supra-pressuromodulated gene (Table 2 & Supplementary file 3 – Supplementary Table S3).

### >11,864 ≤265,005 gene base category, *SELE*

For *SELE*, the beginning 5′ -> 3′ episodic character is stabilizing isotropy stabilized mono anisotropy (B) followed dual mesotropy (B); the middle 5′ -> 3′ episodic character is mono anisotropy (M), stabilizing isotropy stabilized mono anisotropy (M), mono anisotropy (M), reverse stabilizing isotropy converted anisotropy-to-mesotropy (M), dual anisotropy (M) followed by mono mesotropy (M); and the ending 5′ -> 3′ episodic character is stabilizing isotropy stabilized mono anisotropy (E) followed by mono anisotropy (E). For *SELE*, the middle 3′ -> 5′ episodic character is anisotropy reverse stabilizing isotropy (M) and anisotropy reverse converting stabilizing isotropy (M). *SELE* is a [5(+2): 7] SEB Episode 2 gene (Table 1).

For *SELE*, there are three final MSEBs and there are four final ASEBs (Figure 9). For *SELE*, the integrated *uppmsebssiwa* is 32,129 at Episode 2 (h = 3) and the integrated *uppasebssiwa* is 8,849 at Episode 2 (d = 4); and the integrated *dppmsebssiwa* is 84,404 at Episode 2 (h = 3) and the integrated *dppasebssiwa* is 120,813 at Episode 2 (d = 4). For *SELE*, the *uppesebssiwaa* is 20,489 and the *dppesebssiwaa* is 102,609 that results in an *esebssiwaagoT*_Q_ of 0.20 at Episode 2. *SELE* meets the threshold of < 0.25 for an infra-pressuromodulated gene (Table 2 & Supplementary file 3 – Supplementary Table S3).

### >11,864 ≤265,005 gene base category, *CDH11*

For *CDH11*, the beginning 5′ -> 3′ episodic character is mono mesotropy (B) followed by reverse stabilizing isotropy stabilized mono anisotropy (B); the middle 5′ -> 3′ episodic character is mono mesotropy (M) followed by dual anisotropy (M); and the ending 5′ -> 3′ episodic character is dual mesotropy (E). For *CDH11*, the middle 3′ -> 5′ episodic character is anisotropy reverse stabilizing isotropy (M). *CDH11* is a (5) SEB Episode 2 gene (Table 1).

For *CDH11*, there are three final MSEBs and there are two final ASEBs (Figure 10). For *CDH11*, the integrated *uppmsebssiwa* is 42,644 at Episode 2 (h = 3) and the integrated *uppasebssiwa* is 8781 at Episode 2 (d = 2); and the integrated *dppmsebssiwa* is 84,268 at Episode 2 (h = 3) and the integrated *dppasebssiwa* is 333,604 at Episode 2 (d = 2). For *CDH11*, the *uppesebssiwaa* is 25,713 and the *dppesebssiwaa* is 208,936 that results in an *esebssiwaagoT*_Q_ of 0.12 at Episode 2. *CDH11* meets the threshold of < 0.25 for an infra-pressuromodulated gene (Table 2 & Supplementary file 3 – Supplementary Table S3).

### >11,864 ≤265,005 gene base category, *ZCCHC2*

For *ZCCHC2*, the beginning 5′ -> 3′ episodic character is mono anisotropy (B), reverse stabilizing isotropy converted anisotropy-to-mesotropy (B), mono anisotropy (B), dual stabilizing isotropy converted anisotropy-to-mesotropy (B) followed by stabilizing isotropy stabilized mono mesotropy (B); the middle 5′ -> 3′ episodic character is mono anisotropy (M) followed by dual mesotropy (M); and the ending 5′ -> 3′ episodic character is multi (5) anisotropy (E). For *ZCCHC2*, the beginning 3′ -> 5′ episodic character is anisotropy reverse stabilizing converting isotropy (B). *ZCCHC2* is a (5 [+2]: 7) SEB Episode 2 gene (Table 1).

For *ZCCHC2*, there are three final MSEBs and there are four final ASEBs (Figure 11). For *ZCCHC2*, the integrated *uppmsebssiwa* is 34,591 at Episode 2 (h = 3) and the integrated *uppasebssiwa* is 25,000 at Episode 2 (d = 4); and the integrated *dppmsebssiwa* is 97,524 at Episode 2 (h = 3) and the integrated *dppasebssiwa* is 246,271 at Episode 2 (d = 4). For *ZCCHC2*, the *uppesebssiwaa* is 29,796 and the *dppesebssiwaa* is 171,898 that results in an *esebssiwaagoT*_Q_ of 0.17 at Episode 2. *ZCCHC2* meets the threshold of < 0.25 for an infra-pressuromodulated gene (Table 2 & Supplementary file 3 – Supplementary Table S3).

### ≤ 11,864 gene base category, *S100A2*

For *S100A2*, the beginning 5′ -> 3′ episodic character is reverse stabilizing isotropy stabilized mono anisotropy (B) followed by dual mesotropy (B); the middle 5′ -> 3′ episodic character is mono anisotropy (M), reverse stabilizing isotropy stabilized mono mesotropy (M), multi anisotropy (M), mono mesotropy (M) followed by stabilizing isotropy and reverse stabilizing isotropy stabilized mono mesotropy (M); and the ending 5′ -> 3′ episodic character is stabilizing isotropy stabilized mono anisotropy (E). For *S100A2*, the beginning 3′ -> 5′ episodic character is anisotropy reverse stabilizing isotropy (B); and the middle 3′ -> 5′ episodic character is mesotropy reverse stabilizing isotropy (M) and mesotropy reverse stabilizing isotropy (M). *S100A2* is a (7) SEB Episode 3 gene (Table 1).

For *S100A2*, there are three final MSEBs and there are four final ASEBs (Figure 12). For *S100A2*, the integrated *uppmsebssiwa* is 17,298 at Episode 3 (h = 3) and the integrated *uppasebssiwa* is 3916 at Episode 3 (d = 4); and the integrated *dppmsebssiwa* is 29,298 at Episode 3 (h = 3) and the integrated *dppasebssiwa* is 40,214 at Episode 3 (d = 4). For *S100A2*, the *uppesebssiwaa* is 10,838 and the *dppesebssiwaa* is 34,756 that results in an *esebssiwaagoT*_Q_ of 0.31 at Episode 3. *S100A2* meets the threshold of ≥ 0.25 < 0.75 for a supra-pressuromodulated gene (Table 2 Supplementary file 3 – Supplementary Table S3).

### ≤11,864 gene base category, *PRR3*

For *PRR3*, the beginning 5′ -> 3′ episodic character is stabilizing isotropy stabilized mono anisotropy (B), mono anisotropy (B) followed by dual mesotropy (B); the middle 5′ -> 3′ episodic character is mono anisotropy (M), reverse stabilizing isotropy converted anisotropy-to-mesotropy (M), dual anisotropy (M), stabilizing isotropy stabilized mono mesotropy (M), dual mesotropy (M), multi (6) anisotropy (M) followed by multi mesotropy (M); and the ending 5′ -> 3′ episodic character is multi (6) anisotropy (E). For *PRR3*, the middle 3′ -> 5′ episodic character is anisotropy reverse stabilizing isotropy (M). *PRR3* is a [7(+2): 9] SEB Episode 3 gene (Table 1).

For *PRR3*, there are four final MSEBs and there are five final ASEBs (Figure 13). For *PRR3*, the integrated *uppmsebssiwa* is 14,114 at Episode 3 (h = 4) and the integrated *uppasebssiwa* is 5063 at Episode 3 (d = 5); and the integrated *dppmsebssiwa* is 27,585 at Episode 3 (h = 4) and the integrated *dppasebssiwa* is 53,078 at Episode 3 (d = 5). For *PRR3*, the *uppesebssiwaa* is 9588 and the *dppesebssiwaa* is 40,331 that results in an *esebssiwaagoT*_Q_ of 0.24 at Episode 3. *PRR3* meets the threshold of < 0.25 for an infra-pressuromodulated gene (Table 2 & Supplementary file 3 – Supplementary Table S3).

### ≤11,864 gene base category, *IFI27*

For *IFI27*, the beginning 5′ -> 3′ episodic character is mono anisotropy (B) followed by mono mesotropy (B); the middle 5′ -> 3′ episodic character is dual anisotropy (M), tri mesotropy (M), dual anisotropy (M), stabilizing isotropy stabilized mono anisotropy (M), dual anisotropy (M) followed by stabilizing isotropy and reverse stabilizing isotropy stabilized mono mesotropy (M); and the ending 5′ -> 3′ episodic character is mono anisotropy (E). For *IFI27*, the beginning 3′ -> 5′ episodic character is mesotropy reverse stabilizing isotropy (B). *IFI27* is a (7) SEB Episode 3 gene (Table 1).

For *IFI27*, there are three final MSEBs and there are 4 final ASEBs (Figure 14). For *IFI27*, the integrated *uppmsebssiwa* is 42,010 at Episode 3 (h = 3) and the integrated *uppasebssiwa* is 18,359 at Episode 3 (d = 4); and the integrated *dppmsebssiwa* is 108,053 at Episode 3 (h = 3) and the integrated *dppasebssiwa* is 151,833 at Episode 3 (d = 4). For *IFI27*, the *uppesebssiwaa* is 30,185 and the *dppesebssiwaa* is 129,943 that results in an *esebssiwaagoT*_Q_ of 0.23 at Episode 3. *IFI27* meets the threshold of < 0.25 for an infra-pressuromodulated gene (Table 2 & Supplementary file 3 – Supplementary Table S3).

### ≤11,864 gene base category, *S100A14*

For *S100A14*, the beginning 5′ -> 3′ episodic character is tri mesotropy (B), stabilizing isotropy stabilized mono mesotropy (B), stabilizing isotropy stabilized mono mesotropy (B), stabilizing isotropy converted mono anisotropy-to-mesotropy (B) followed by mono anisotropy (B); the middle 5′ -> 3′ episodic character is dual mesotropy (M), stabilizing isotropy stabilized mono anisotropy (M), mono anisotropy (M), dual stabilizing isotropy and dual reverse stabilizing isotropy stabilized mono mesotropy (M), mono mesotropy (M) followed by multi anisotropy (M); and the ending 5′ -> 3′ episodic character is stabilizing isotropy stabilized mono mesotropy (E). For *S100A14*, the middle 3′ -> 5′ episodic character is mesotropy reverse stabilizing isotropy (M), and mesotropy reverse stabilizing isotropy (M). *S100A14* is a (7) SEB Episode 3 gene (Table 1).

For *S100A14*, there are four final MSEBs and there are three final ASEBs (Figure 15). For *S100A14*, the integrated *uppmsebssiwa* is 21,523 at Episode 3 (h = 4) and the integrated *uppasebssiwa* is 8033 at Episode 3 (d = 3); and the integrated *dppmsebssiwa* is 53,204 at Episode 3 (h = 4) and the integrated *dppasebssiwa* is 188,934 at Episode 3 (d = 3). For *S100A14*, the *uppesebssiwaa* is 14,778 and the *dppesebssiwaa* is 121,069 that results in an *esebssiwaagoT*_Q_ of 0.12 at Episode 3. *S100A14* meets the threshold of < 0.25 for an infra-pressuromodulated gene (Table 2 & Supplementary file 3 – Supplementary Table S3).

### >265,005 <607,463 gene base category, *ABCB1*

For *ABCB1*, the beginning 5′ -> 3′ episodic character is stabilizing isotropy stabilized, reverse stabilizing isotropy stabilized, reverse stabilizing isotropy stabilized and stabilizing isotropy stabilized mono mesotropy (B) followed by tri anisotropy (B); the middle 5′ -> 3′ episodic character is dual mesotropy (M), mono anisotropy (M), mono mesotropy (M), tri anisotropy (M), mono mesotropy (M) followed by stabilizing isotropy and reverse stabilizing isotropy stabilized mono mesotropy (M); and the ending 5′ -> 3′ episodic character is stabilizing isotropy converted anisotropy-to-mesotropy (E) followed by dual mesotropy (E). For *ABCB1*, the beginning 3′ -> 5′ episodic character is mesotropy reverse stabilizing isotropy (B) and mesotropy reverse stabilizing isotropy (B); and the middle 3′ -> 5′ episodic character is mesotropy reverse stabilizing isotropy (M). *ABCB1* is a [9(-2): 7] SEB Episode 4 gene (Table 1).

For *ABCB1*, there are four final MSEBs and there are three final ASEBs (Figure 16). For *ABCB1*, the integrated *uppmsebssiwa* is 74,163 at Episode 4 (h = 4) and the integrated *uppasebssiwa* is 18,256 at Episode 4 (d = 3); and the integrated *dppmsebssiwa* is 161,925 at Episode 4 (h = 4) and the integrated *dppasebssiwa* is 355,477 at Episode 4 (d = 3). For *ABCB1*, the *uppesebssiwaa* is 46,210 and the *dppesebssiwaa* is 258,701 that results in an *esebssiwaagoT*_Q_ of 0.18 at Episode 4. *ABCB1* meets the threshold of < 0.25 for an infra-pressuromodulated gene (Table 2 & Supplementary file 3 – Supplementary Table S3>).

### ≥ 607,463 < 2,241,933 gene base category, *FOXP2*

For *FOXP2*, the beginning 5′ -> 3′ episodic character is reverse stabilizing isotropy converted mono mesotropy-to-stabilizing isotropy and stabilized mono mesotropy (B) followed by mono anisotropy (B); the middle 5′ -> 3′ episodic character is tri mesotropy (M), multi (4) anisotropy (M), dual mesotropy (M), mono anisotropy (M), stabilizing isotropy stabilized mono mesotropy (M), mono mesotropy (M), mono anisotropy (M), dual mesotropy (M) followed by tri anisotropy (M); and the ending 5′ -> 3′ episodic character is mono mesotropy (E). For *FOXP2*, the beginning 3′ -> 5′ episodic character is mesotropy reverse stabilizing converting isotropy (B). *FOXP2* is a (11) SEB Episode 5 gene (Table 1).

For *FOXP2*, there are six final MSEBs and there are five final ASEBs (Figure 17). For *FOXP2*, the integrated *uppmsebssiwa* is 69,583 at Episode 5 (h = 6) and the integrated *uppasebssiwa* is 15,948 at Episode 5 (d = 5); and the integrated *dppmsebssiwa* is 140,583 at Episode 5 (h = 6) and the integrated *dppasebssiwa* is 273,470 at Episode 5 (d = 5). For *FOXP2*, the *uppesebssiwaa* is 42,754 and the *dppesebssiwaa* is 207,027 that results in an *esebssiwaagoT*_Q_ of 0.21 at Episode 5. *FOXP2* meets the threshold of < 0.25 for an infra-pressuromodulated gene (Table 2; Supplementary file 3 – Supplementary Table S3).

### ≥ 2,241,933 gene base category, *DMD*

For *DMD*, the beginning 5′ -> 3′ episodic character is mono anisotropy (B), mono mesotropy (B) followed stabilizing isotropy and tempered considered (TC) part-reverse stabilizing isotropy stabilized mono mesotropy (B); the middle 5′ -> 3′ episodic character is tri anisotropy (M), stabilizing isotropy stabilized mono mesotropy (M), mono mesotropy (M), dual anisotropy (M), mono mesotropy (M), stabilizing isotropy stabilized mono anisotropy (M), multi (6) anisotropy (M), mono mesotropy (M), mono anisotropy (M), mono mesotropy (M), mono anisotropy (M) followed by mono mesotropy (M); and the ending 5′ -> 3′ episodic character is mono anisotropy (E). For *DMD*, the beginning 3′ -> 5′ episodic character is mesotropy reverse stabilizing isotropy (B), reverse anisotropy (B) and reverse anisotropy (B). *DMD* is a (13) SEB Episode 6 gene (Table 1).

For *DMD*, there are six final MSEBs, and there are seven final ASEBs. The tempered considered (TC) 3′ -> 5′ reverse isotropy point (253,856, 280,037) of first *MSEBS* is upstream intergene distance 0.125-factor adjusted to 31,732, 31,732 instead of upstream intergene distance 0.25-factor adjusted to 63,464, 63,464 as there are two immediately preceding reverse anisotropy points (167,442, 830,657; 5,488, 272,663) of sufficient 3′ -> 5′ reverse anisotropy to diminish the 3′ -> 5′ reverse isotropy stabilizing effect of 253,856, 280,037 to 31,732, 31,732 (Figure 18). For *DMD*, the integrated *uppmsebssiwa* is 74,292 at Episode 6 (h = 6) and the integrated *uppasebssiwa* is 32,973 at Episode 6 (d = 7); and the integrated *dppmsebssiwa* is 163,570 at Episode 6 (h = 6) and the integrated *dppasebssiwa* is 296,028 at Episode 6 (d = 7). For *DMD*, the *uppesebssiwaa* is 53,632 and the *dppesebssiwaa* is 229,799 that results in an *esebssiwaagoT*_Q_ of 0.23 at Episode 6. *DMD* meets the threshold of < 0.25 for an infra-pressuromodulated gene (Table 2 & Supplementary file 3 – Supplementary Table S3).

## Discussion

### 5′ -> 3′ direction paired point tropy quotients (*prpT*_Q_s) for characterization of intergene distance pair SEB episodicity

The 3′ -> 5′ and 5′ -> 3′ direction paired point tropy quotients (*prpT*_Q_s) represent the point-by-point 3′ -> 5′ and 5′ -> 3′ direction upstream and downstream intergene distance pair tropies from the gene of interest, respectively, to achieve the nth order of 5′ -> 3′ direction intergene distance pair tropies for the initial number of SEBs to establish the episodicity per gene category.

The transcribing 5′ -> 3′ direction intergene segment pair tropies are necessary to establish the initial anisotropic (single point, dual point, triple point or multiple point SEB; each *prpT*_Q_ point of SEB < 0.25) and mesotropic (single point, dual point, triple point or multiple point SEB; each *prpT*_Q_ point ≥ 0.25 < 0.75) periodicity for determination of the number SEBs for the gene of interest, five initial SEBs for Episode 2 category genes, seven initial SEBs for Episode 3 category genes, nine initial SEBs for Episode 4 category genes, 11 initial SEBs for Episode 5 category genes and 13 initial SEBs for Episode 6 category genes.

Upon establishment of the initial subepisodic block episodicity, there is further consideration of:

The instances where there are preceding transcribing 5′ -> 3′ direction stabilizing isotropy *prpT*_Q_s (5′ -> 3′ stIsotropy *prpT*_Q_s ≥ 0.75), as 0.25 factor-adjusted 5′ -> 3′ direction stabilizing isotropies for part-dependent contribution to increasing the magnitude of tropy effect of the immediately following *prpT*_Q_ point of the following SEB, in which case the affected *prpT*_Q_ point of the SEB may or may not remain anisotropic (anisotropy-to-mesotropy converted tropy) or mesotropic (mesotropy-to-stabilizing isotropy converted tropy) (initial SEB +/- 2 per interconversion); and of;
The instances where there are preceding nontranscribing 3′ -> 5′ direction reverse stabilizing isotropy *prpT*_Q_s (3′ -> 5′ stIsotropy *prpT*_Q_s ≥ 0.75), either as 0.25 factor-adjusted for immediately preceding 3′ -> 5′ direction reverse stabilizing isotropy (ies) for part-dependent contribution to increasing the magnitude of tropy effect of the immediately following *prpT*_Q_ point of the following SEB, or as a 0.125 factor-adjusted for interposed preceding 3′ -> 5′ direction reverse stabilizing isotropy within a series of 3′ -> 5′ reverse anisotropy *prpT*_Q_s (3′ -> 5′ *prpT*_Q_s < 0.25) for less that part-dependent contribution to increasing the magnitude of tropy effect of the immediately following *prT*_Q_ point of the following SEB, in which case the affected SEB also may or may not remain anisotropic (anisotropy-to-mesotropy converted tropy) or mesotropic (mesotropy-to-stabilizing isotropy converted tropy) (initial SEB +/- 2 per inter-conversion).

The transcribing 5′ -> 3′ direction intergene segment pair tropy method establishes the initial number of SEBs, and excludes the number of SEB interconversions and the final number of SEBs, based on which the initial number of episodes for a gene of interest can be determined with certainty (i.e., five initial SEBs = 2 episodes for Episode 2 category genes).

### Final 5′ -> 3′ direction episodic sub-episode block sums split-integrated weighted average-averaged gene overexpression tropy quotient (*esebssiwaagoT_Q_*) for supra-pressuromodulated & infra-pressuromodulated genes

The final 5′ -> 3′ direction episodic sub-episode block sums split-integrated weighted average-averaged gene overexpression tropy quotient (*esebssiwaagoT_Q_*) represents for example:

The serially split-integrated averaged anisotropic part SEB sums (*ASEBS*) to the *n* ^th^ anisotropic SEBS (i.e., anisotropic SEBS 1 + anisotropic SEBS 2 + anisotropic SEBS 3/3 = the third split-integrated average anisotropic SEBS [*uppasebssiwa*, *dppasebssiwa*]); and
The serially split-integrated averaged mesotropic part SEB sums (*MSEBS*) to the *n* ^th^ mesotropic SEBS (i.e., mesotropic SEBS 1 + mesotropic SEBS 2/2 = the second split-integrated average mesotropic SEBS) (*uppmsebssiwa*, *dppmsebssiwa*), respectively; thereafter,
The *uppasebssiwa* and the *uppasebssiwa* averaged together for the *uppesebssiwaa*, and the *dppasebssiwa* and the *dppasebssiwa* averaged together for the *dppesebssiwaa*, whereby the *uppesebssiwaa*/*dppesebssiwaa* yield the *esebssiwaagoT_Q_* at the Episode 2 fifth SEB, which would be the SEB count in the case of a non-converted Episode 2 category gene. As indicated above, in the case of both the anisotropic intergene distance segment SEB sums (*ASEBS*) and the mesotropic intergene distance segment SEB sums (*MSEBS*), each of steps prior the final calculation step is done in upstream part (*upp-*) and downstream part (*dpp-*) intergene distance segment SEBSs (*uppASEBSs*, *dppASEBSs*; *uppMSEBSs*, *dppMSEBSs*) (see ‘Methods’ section for detail).

For the >11,864 ≤265,005 gene base category (i.e., *SORL1*, *PDPN*, *BTG1*, *HAPLN1*, *MRC1*, *ACPP*, *TGFA*, *PHLPP1*, *SELE*, *CDH11*, *ZCCHC2*), the final *esebssiwaagoT*_Q_ is to the end of Episode 2, which implies that intermediate genes appear to be most sensitive to the cellular pressuromodulation effect. In contrast, for the ≤ 11,864 gene base category (i.e., *S100A2*, *PRR3*, *IFI27*, *S100A14*), the final *esebssiwaagoT*_Q_ is at the end of Episode 3, which implies that smaller genes appear to be less sensitive to cellular pressuromodulation effect. For the > 265,005 < 607,463 gene base category (i.e., *ABCB1*), the final *esebssiwaagoT_Q_* is at the end of Episode 4; for the ≥ 607,463 < 2,241,933 gene base category (i.e., *FOXP2*), the final *esebssiwaagoT*_Q_ is at the end of Episode 5; and for the ≥ 2,241,933 gene base category, the final *esebssiwaagoT*_Q_ is at the end of Episode 6 (i.e., *DMD*), which implies that larger genes appear to also be less sensitive to cellular pressuromodulation effect.

The final *esebssiwaagoT*_Q_ classifies a LEnC overexpressed gene as a supra-pressuromodulated gene (*esebssiwaagoT_Q_* ≥ 0.25 < 0.75) every time and classifies a BMEnC overexpressed gene every time as an infra-pressuromodulated gene (*esebssiwaagoT*_Q_ < 0.25) (100% sensitivity; 100% specificity), and therefore, is 100% accurate.

### Relevance of the final *esebssiwaagoT_Q_* for classification of genes as either supra-pressuromodulated or infra-pressuromodulated

Genes can be classified as either as a supra-pressuromodulated gene (Supra: *esebssiwaagoT*_Q_ ≥ 0.25 < 0.75) or as an infra-pressuromodulated gene (Infra: *esebssiwaagoT*_Q_ < 0.25) with accuracy.

It can be expected that the expression of a Supra or Infra gene will correlate with the pressuromodulation state of a cell type, in which case the most pressuromodulated cell types should express a Supra gene at the highest level, while the least pressuromodulated cell types should express a Supra gene at the lowest level; whereas, the least pressuromodulated cell types should express an Infra gene at the highest level, while the most pressuromodulated cell type should express an Infra gene at the lowest level. This being the case, all Supra genes will be overexpressed in response to increases in cell membrane pressuromodulation, while being underexpressed in response to decreases in cell membrane pressuromodulation; and all Infra genes will be overexpressed in response to decreases in cell membrane pressuromodulation, while being underexpressed in response to increases in cell membrane pressuromodulation. It can be further postulated that there is a graded decrease in the pressuromodulation state of the cell in the progression from zygote (spermatocyte oocyte fusion) totipotency-to-pluripotency-to-differentiation, in which case Supra gene expression would be gradedly lesser in the spectrum toward differentiation away from pluripotency including in the case of Supra gene quintessential Supra transcription factor adapter gene expression, while Infra gene expression would be gradedly greater in the spectrum toward differentiation away from pluripotency including in the case of Infra gene quintessential Infra transcription factor adapter gene expression.

Based on the methodology of this research study all genes can be classified as either Supra (*esebssiwaagoT*_Q_ ≥ 0.25 < 0.75) or Infra (*esebssiwaagoT*_Q_ < 0.25) with accuracy. It is further envisioned that early passage primary cells can be rank-ordered by cell pressuromodulation state with additional knowledge of Supra and Infra gene expression level differences between cell types, in which case limiting Supra and Infra transcription factor gene and the limiting Supra and Infra transcription factor adapter gene expression differences between cell types could provide further valuable insight into cell lineage fates.

## Conclusion

Based on the findings of this study, an infra-pressuromodulated gene (Infra: *esebssiwaagoT*_Q_ < 0.25) requires lesser cellular pressuromodulation to be overexpressed, that is, to become optimally horizontally aligned for transcription, in contrast to a supra-pressuromodulated gene (Supra: *esebssiwaagoT*_Q_ ≥ 0.25 < 0.75) that requires greater cellular pressuromodulation to be overexpressed, that is, to become optimally horizontally aligned for transcription, when an infra-pressuromodulated gene becomes less than optimally horizontally aligned. Therefore, horizontal alignment of 5′ -> 3′ direction intergene distance segment tropy with respect to the gene is the conserved basis for DNA transcription for genes in the pressuromodulated state. This finding is ubiquitously applicable, as it would, for example, also be the basis for viral DNA or RNA stand replication and transcription, for viral DNA or RNA strand transfection vector expression upon integration into the host genome, and for circular bacterial plasmid gene expression, in which case there is gravitational parallel horizontal walking analogous to ‘a rodent on a Ferris Wheel.’

## Future perspective

Based on the findings of this study, the final 5′ -> 3′ *esebssiwaagoT*_Q_ accurately classifies a gene as either a supra-pressuromodulated gene (Supra: *esebssiwaagoT*_Q_ ≥ 0.25 < 0.75) every time or an infra-pressuromodulated gene (Infra: *esebssiwaagoT*_Q_ < 0.25) every time (100% sensitivity; 100% specificity), and therefore, is 100% accurate. Therefore, it now becomes possible to classify every gene as either Supra or Infra by applying the *esebssiwaagoT*_Q_, without the need for additional experimental data from cells on opposite ends of the pressuromodulation spectrum.

It can be further postulated that in the multicellular organism, the fact that a wide-spectrum of cell types exist in the biological system is entirely attributable to cell membrane pressuromodulation-mediated differences across cell types in Supra and Infra gene expression levels. As such, with *a priori* knowledge of whether a gene is either a Supra or an Infra gene, it would be possible to rank order the entire spectrum of cell types of the multicellular biological system, ranging from pluripotent-to-differentiated (more pressuromodulated normal state-to-less pressuromodulated normal state), as well as those ranging from normal-to-neoplastic (less pressuromodulated normal state-to-more pressuromodulated abnormal state) based on ‘pressuromodulation state indices’ with cDNA microarray-based Supra gene mRNA expression levels and Infra gene mRNA expression levels for a given cell type (Supra-to-Infra index) as well as the same for different cell types (Supra-to-Supra and Infra-to-Infra indices). With such knowledge it will become easy to appreciate that, in fact, cellular pressuromodulation state-mediated changes in Supra and Infra gene expression levels is the likely basis for the wide-spectrum of cellular differentiation in the multicellular biological system as well as the basis for the maintenance of the neoplastic state.

**Table 1.** **Episodic and sub-episodic block character as per the paired point tropy quotients (*prpT*_Q_s).**

**Gene symbol**	**Number of episodes (number of final SEBs)**	**Beginning (B), middle (M) and ending (E) of episodic character as per 5′ -> 3′ *prpT_Q_*s (SEB character)**	**Episodic character as per 3′ -> 5′ *prpT_Q_*s**	**Gene type**
*SORL1*	2 (5 [-2]: 3)	(1) Dual mesotropy (B) (2) Dual anisotropy deviation from constancy variant (B) (3a) Dual mesotropy (M) (3b) Stabilizing isotropy stabilized mono mesotropy (M) (3c) Reverse stabilizing isotropy converted mono mesotropy-to-stabilizing isotropy and stabilized converted mono anisotropy-to-mesotropy (M) (3d) Multi mesotropy (E)	Mesotropy reverse stabilizing converting isotropy (M)	Supra
*PDPN*	2 (5)	(1a) Reverse stabilizing isotropy converted mono mesotropy-to-stabilizing isotropy and stabilized mono mesotropy (B) (1b) Mono mesotropy (B) (2) Mono anisotropy (B) (3a) Tri mesotropy (M) (3b) Stabilizing isotropy and reverse stabilizing isotropy stabilized mono mesotropy (M) (3c) Stabilizing isotropy stabilized mono mesotropy (M) (4) Mono anisotropy (M) (5)Reverse stabilizing isotropy stabilized mono mesotropy (E)	Mesotropy reverse stabilizing converting isotropy (B) Mesotropy reverse stabilizing isotropy (M) Mesotropy reverse stabilizing isotropy (E)	Supra
*BTG1*	2 (5)	(1) Tri anisotropy (B) (2a) Tri mesotropy (B) (2b) Reverse stabilizing isotropy stabilized mono mesotropy (B) (2c) Mono mesotropy (B) (NC 3a) non-mono anisotropy (M) (3b) Stabilizing isotropy stabilized mono anisotropy (M) (3c) Mono anisotropy (M) (4) Mono mesotropy (M) (5a) Reverse stabilizing isotropy stabilized mono anisotropy (E) (5b) Dual anisotropy (E)	Mesotropy reverse stabilizing isotropy (B) Reverse anisotropy (M), Anisotropy reverse stabilizing isotropy (E)	Supra
*HAPLN1*	2 (5 [+2]: 7)	(1) Dual mesotropy (B) (2) Mono anisotropy (B) (3) Stabilizing isotropy converted mono anisotropy-to-mesotropy (B) (4) Stabilizing isotropy stabilized mono anisotropy (B) (5a) Mono mesotropy (M) (5b) Reverse stabilizing isotropy stabilized mono mesotropy (M) (5c) Mono mesotropy (M) (6) Mono anisotropy (M) (7) Mono mesotropy (E)	Mesotropy reverse stabilizing isotropy (M)	Supra
*MRC1*	2 (5 [+2]: 7)	(1) Multi (6) anisotropy (B) (2) Stabilizing isotropy and reverse stabilizing isotropy converted mono anisotropy-to-mesotropy (B) (3) Mono anisotropy (B) (4a) Stabilizing isotropy stabilized mono mesotropy (B) (4b) Stabilizing isotropy stabilized mono mesotropy (B) (5) Dual anisotropy (M) (6) Dual mesotropy (M) (7) Multi (4) anisotropy (E)	Anisotropy reverse stabilizing converting isotropy (B)	Supra
*ACPP*	2 (5 [-2]: 3)	(NC) Nonmono anisotropy (B) (1) Mono mesotropy deviation from constancy (B) (2) Mono anisotropy (B) (3a) Mono mesotropy deviation from constancy (M) (3b) Stabilizing isotropy and reverse stabilizing isotropy converted mono anisotropy-to-mesotropy deviation from constancy (M) (3c) Mono mesotropy deviation from constancy (E)	Reverse anisotropy (B)	Supra
*TGFA*	2 (5)	(1a) Stabilizing isotropy stabilized mono mesotropy (B) (1b) Dual mesotropy (B) (2a) Mono anisotropy (B) (2b) Reverse stabilizing isotropy stabilized mono anisotropy (B) (2c) Mono anisotropy (B) (3a) Mono mesotropy (M) (3b) Reverse stabilizing isotropy stabilized mono mesotropy (M) (3c) Stabilizing isotropy and reverse stabilizing isotropy converted mono anisotropy-to-mesotropy (M) (4) Mono anisotropy (M) (5) Tri mesotropy (E)	Anisotropy reverse stabilizing isotropy (B) Mesotropy reverse stabilizing isotropy (M) Anisotropy reverse stabilizing converting isotropy (M)	Supra
*PHLPP1*	2 (5)	(1) Tri mesotropy (B) (2) Mono anisotropy (B) (3) Stabilizing isotropy and reverse stabilizing isotropy converted mono mesotropy-to stabilizing isotropy and stabilized mono mesotropy (M) (4a) Stabilizing isotropy stabilized mono anisotropy (M) (4b) Mono anisotropy (M) (5) Mono mesotropy (E)	Mesotropy reverse stabilizing converting isotropy (M)	Supra
*SELE*	2 (5 [+2]: 7)	(1) Stabilizing isotropy stabilized mono anisotropy (B) (2) Dual mesotropy (B) (3a) Mono anisotropy (M) (3b) Stabilizing isotropy stabilized mono anisotropy (M) (3c) Mono anisotropy (M) (4) Reverse stabilizing isotropy converted anisotropy-to-mesotropy (M) (5) Dual anisotropy (M) (6) Mono mesotropy (M) (7a) Stabilizing isotropy stabilized mono anisotropy (E) (7b) Mono anisotropy (E)	Anisotropy reverse stabilizing isotropy (M) Anisotropy reverse converting stabilizing isotropy (M)	Infra
*CDH11*	2 (5)	(1) Mono mesotropy (B) (2) Reverse stabilizing isotropy stabilized mono anisotropy (B) (3) Mono mesotropy (M) (4) Dual anisotropy (M) (5) Dual mesotropy (E)	Anisotropy reverse stabilizing isotropy (M)	Infra
*ZCCHC2* (*C18orf49*; *KIAA1744*)	2 (5 [+2]: 7)	(1) Mono anisotropy (B) (2) Reverse stabilizing isotropy converted anisotropy-to-mesotropy (B) (3) Mono anisotropy (B) (4a) Dual stabilizing isotropy converted anisotropy-to-mesotropy (B) (4b) Stabilizing isotropy stabilized mono mesotropy (B) (5) Mono anisotropy (M) (6) Dual mesotropy (M) (7) Multi (5) anisotropy (E)	Anisotropy reverse stabilizing converting isotropy (B)	Infra
*S100A2*	3 (7)	(1) Reverse stabilizing isotropy stabilized mono anisotropy (B) (2) Dual mesotropy (B) (3) Mono anisotropy (M) (4) Reverse stabilizing isotropy stabilized mono mesotropy (M) (5) Multi anisotropy (M) (6a) Mono mesotropy (M) (6b) Stabilizing isotropy and reverse stabilizing isotropy stabilized mono mesotropy (M) (7) Stabilizing isotropy stabilized mono anisotropy (E)	Anisotropy reverse stabilizing isotropy (B) Mesotropy reverse stabilizing isotropy (M) Mesotropy reverse stabilizing Isotropy (M)	Supra
*PRR3*	3 (7 [+2]: 9)	(1a) Stabilizing isotropy stabilized mono anisotropy (B) (1b) Mono anisotropy (B) (2) Dual mesotropy (B) (3) Mono anisotropy (M) (4) Reverse stabilizing isotropy converted anisotropy-to-mesotropy (M) (5) Dual anisotropy (M) (6a) Stabilizing isotropy stabilized mono mesotropy (M) (6b) Dual mesotropy (M) (7) Multi (6) anisotropy (M) (8) Multi mesotropy (M) (9) Multi (6) anisotropy (E)	Anisotropy reverse stabilizing isotropy (M)	Infra
*IFI27*	3 (7)	(1) Mono anisotropy (B) (2) Mono mesotropy (B) (3) Dual anisotropy (M) (4) Tri mesotropy (M) (5a) Dual anisotropy (M) (5b) Stabilizing isotropy stabilized mono anisotropy (M) (5c) Dual anisotropy (M) (6) Stabilizing isotropy and reverse stabilizing isotropy stabilized mono mesotropy (M) (7) Mono anisotropy (E)	Mesotropy reverse stabilizing isotropy (B)	Infra
*S100A14*	3 (7)	(1a) Tri mesotropy (B) (1b) Stabilizing isotropy stabilized mono mesotropy (B) (1c) Stabilizing isotropy stabilized mono mesotropy (B) (1d) Stabilizing isotropy converted mono anisotropy-to-mesotropy (B) (2) Mono anisotropy (B) (3) Dual mesotropy (M) (4a) Stabilizing isotropy stabilized mono anisotropy (M) (4b) Mono anisotropy (M) (5a) Dual stabilizing isotropy and dual reverse stabilizing isotropy stabilized mono mesotropy (M) (5b) Mono mesotropy (M) (6) Multi anisotropy (M) (7) Stabilizing isotropy stabilized mono mesotropy (E)	Mesotropy reverse stabilizing isotropy (M) Mesotropy reverse stabilizing isotropy (M)	Infra
*ABCB1*	4 (9 [-2]: 7)	(1) Stabilizing isotropy stabilized, reverse stabilizing isotropy stabilized, reverse stabilizing isotropy stabilized and stabilizing isotropy stabilized mono mesotropy (B) (2) Tri anisotropy (B) (3) Dual mesotropy (M) (4) Mono anisotropy (M) (5) Mono mesotropy (M) (6) Tri anisotropy (M) (7a) Mono mesotropy (M) (7b) Stabilizing isotropy and reverse stabilizing isotropy stabilized mono mesotropy (M) (7c) Stabilizing isotropy converted anisotropy-to-mesotropy (E) (7d) Dual mesotropy (E)	Mesotropy reverse stabilizing isotropy (B) Mesotropy reverse stabilizing isotropy (B) Mesotropy reverse stabilizing isotropy (M)	Infra
*FOXP2*	5 (11)	(1) Reverse stabilizing isotropy converted mono mesotropy-to-stabilizing isotropy and stabilized mono mesotropy (B) (2) Mono anisotropy (B) (3) Tri mesotropy (M) (4) Multi (4) anisotropy (M) (5) Dual mesotropy (M) (6) Mono anisotropy (M) (7a) Stabilizing isotropy stabilized mono mesotropy (M) (7b) Mono mesotropy (M) (8) Mono anisotropy (M) (9) Dual mesotropy (M) (10) Tri anisotropy (M) (11) Mono mesotropy (E)	Mesotropy reverse stabilizing converting isotropy (B)	Infra
*DMD*	6 (13)	(1) Mono anisotropy (B) (2a) Mono mesotropy (B) (2b) Stabilizing isotropy and part-reverse stabilizing isotropy stabilized mono mesotropy (B) (3) Tri anisotropy (M) (4a) Stabilizing isotropy stabilized mono mesotropy (M) (4b) Mono mesotropy (M) (5) Dual anisotropy (M) (6) Mono mesotropy (M) (7a) Stabilizing isotropy stabilized mono anisotropy (M) (7b) Multi (6) anisotropy (M) (8) Mono mesotropy (M) (9) Mono anisotropy (M) (10) Mono mesotropy (M) (11) Mono anisotropy (M) (12) Mono mesotropy (M) (13) Mono anisotropy (E)	(TC) Mesotropy part-reverse stabilizing isotropy (B) Reverse anisotropy (B) Reverse anisotropy (B)	Infra

**Table 2.** **Final episodic sub-episode block sums split-integrated weighted average-averaged gene overexpression tropy quotient (*esebssiwaagoT*_Q_) per gene category.**

**Gene symbol**	**Number of transcribed gene bases**	**Gene base category**	***Uppasebssiwa* + *uppmsebssiwa*** **2**	***Dppasebssiwa* + *dppmsebssiwa*** **2**	***uppesebssiwaa*** ***dppesebssiwaa***	***esebssiwaagoT*_Q_**	**Gene type**
*SORL1*	181,560 (197,782)	>11,864 ≤ 265,005	33,201 + 65,960 2	203,780 + 144,058 2	49,581 173,919	0.29 (@ Episode 2)	Supra
*PDPN*	34,493	> 11,864 ≤ 265,005	2867 + 25,875 2	24,905 + 45,697 2	14,371 35,301	0.41 (@ Episode 2)	Supra
*BTG1*	5620 (160,922)	>11,864 ≤265,005	23,241 + 84,939 2	235,679 + 188,894 2	54,090 212,287	0.25 (@ Episode 2)	Supra
*HAPLN1*	83,809	>11,864 ≤265,005	11,635 + 71,228 2	90,544 + 144,030 2	41,431 117,287	0.35 (@ Episode 2)	Supra
*MRC1*	101,828	>11,864 ≤265,005	29,632 + 50,393 2	198,095 + 91,136 2	46,390 148,945	0.28 (@ Episode 2)	Supra
*ACPP*	50,936	>11,864 ≤265,005	3600 + 34,438 2	56,613 + 96,721 2	19,019 76,667	0.25 (@ Episode 2)	Supra
*TGFA*	106,914	>11,864 ≤265,005	9528 + 53,114 2	84,199 + 124,663 2	24,929 81,129	0.31 (@ Episode 2)	Supra
*PHLPP1*	265,005	>11,864 ≤265,005	10,343 + 49,476 2	80,268 + 92,687 2	29,910 86,477	0.35 (@ Episode 2)	Supra
*SELE*	42,066 (74,076)	>11,864 ≤265,005	8849 + 32,129 2	120,813 + 84,404 2	20,489 102,609	0.20 (@ Episode 2)	Infra
*CDH11*	182,360	>11,864 ≤265,005	8781 + 42,644 2	333,604 + 84,268 2	25,713 208,936	0.12 (@ Episode 2)	Infra
*ZCCHC2*	64,703	>11,864 ≤265,005	25,000 + 34,591 2	246,271 + 97,524 2	29,796 171,898	0.17 (@ Episode 2)	Infra
*S100A2*	6783	≤11,864	3916 + 17,759 2	40,214 + 29,298 2	10,838 34,756	0.31 (@ Episode 3)	Supra
*PRR3*	7988	≤11,864	5063 + 14,114 2	53,078 + 27,585 2	9588 40,331	0.24 (@ Episode 3)	Infra
*IFI27*	11,864	≤11,864	18,359 + 42,010 2	151,833 + 108,053 2	30,185 129,943	0.23 (@ Episode 3)	Infra
*S100A14*	2732 (4051)	≤11,864	8033 + 21,523 2	188,934 + 53,204 2	14,778 121,069	0.12 (@ Episode 3)	Infra
*ABCB1*	209,691 (386,184)	>265,005 <607,463	18,256 + 74,163 2	355,477 + 161,925 2	46,210 258,701	0.18 (@ Episode 4)	Infra
*FOXP2*	607,463	≥607,463 <2,241,933	15,948 + 69,583 2	273,470 + 140,583 2	42,754 207,027	0.21 (@ Episode 5)	Infra
*DMD*	2,241,933	≥2,241,933	32,973 + 74,292 2	296,028 + 163,570 2	53,632 229,799	0.23 (@ Episode 6)	Infra

**Executive summary**

Comparative cell types at opposite ends of the cell pressuromodulation spectrum include, for example, the lymphatic endothelial cell (LEnC) is a mitogenic without division cell, which is an over-pressuromodulated cell, while the blood microvascular capillary endothelial cell (BMEnC) is non-mitogenic cell, which is an under-pressuromodulated cell; and the multi-nucleated giant cell is also an over-pressuromodulated cell, while the macrophage (mono-nucleated) is an under-pressuromodulated cell, both model cell type-pairs for comparing cell type cDNA microarray mRNA expression levels.
Seven sets of most differentially overexpressed LEnC and BMEnC genes (nonadjusted > twofold) and two sets of juxtaposed lesser differentially overexpressed LEnC and BMEnC genes (nonadjusted one- to two-fold) were selected from a published open access dataset. For these 18 genes, all of the transcribed loci base locations, both protein coding and noncoding, were mined online. The nontranscribing intergene distances were determined upstream and downstream for each gene *wrt* gene. The transcribing 3′ -> 5′ direction and 5′ -> 3′ *prpT*_Q_s (fract) were determined, as were the number of *initial* anisotropic and mesotropic sub-episode blocks (ASEB, MSEB) for each gene categorized by number of bases [>11,864 ≤265,005 (five sub-episode blocks, 5 SEBs); Episode 2]; ≤11,864 (seven SEBs; Episode 3); >265,005 <607,463 (nine SEBs; Episode 4); ≥ 607,463 < 2,241,933 (11 SEBs; Episode 5); ≥2,241,933 (13 SEBs; Episode 6)]. The 5′ -> 3′ upstream part anisotropic sub-episode block sums (*uppASEBS*) split-integrated weighted average (*uppasebssiwa*), the 5′ -> 3′ downstream part anisotropic sub-episode block sums (*dppASEBS*) split-integrated weighted average (*dppasebssiwa*), the 5′ -> 3′ upstream part mesotropic sub-episode block sums (*uppMSEBS*) split-integrated weighted average (*uppmsebssiwa*), and the 5′ -> 3′ downstream part mesotropic sub-episode block sums (*dppMSEBS*) split-integrated weighted average (*dppmsebssiwa*) were determined, based on which the final 5′ -> 3′ upstream part episodic sub-episode block sums split-integrated weighted average-average (*uppesebssiwaa*) and the final 5′ -> 3′ downstream part episodic sub-episode block sums split-integrated weighted average-average (*dppesebssiwaa*) and were determined, whereby the final complete episodic sub-episode block sums split-integrated weighted average-averaged gene overexpression tropy quotient (final complete  *esebssiwaagoT*_Q_) for each gene per category was determined. The 5′ -> 3′ uppASEBS (y-axis), dppASEBS (x-axis) [*ASEBS*], uppMSEBS (y-axis) and dppMSEBS (x-axis) [*MSEBS*], and the final 5′ -> 3′ *uppesebssiwaa* (y-axis) and *dppesebssiwaa* (x-axis) [final complete 5′ -> 3′ *esebssiwaagoT*_Q_] were *log* plotted for each gene.
The final 5′ -> 3′ *esebssiwaagoT*_Q_ classifies a LEnC overexpressed gene as a supra-pressuromodulated gene (*esebssiwaagoT*_Q_ ≥ 0.25 < 0.75) every time and classifies a BMEnC overexpressed gene every time as an infra-pressuromodulated gene (*esebssiwaagoT*_Q_ < 0.25) (100% sensitivity; 100% specificity), therefore a methodology that is 100% accurate.
An infra-pressuromodulated gene (Infra: *esebssiwaagoT*_Q_ < 0.25) requires lesser cellular pressuromodulation to be overexpressed, that is, to become optimally horizontally aligned for transcription, in contrast to a supra-pressuromodulated gene (Supra: *esebssiwaagoT*_Q_ ≥ 0.25 < 0.75) that requires greater cellular pressuromodulation to be overexpressed, that is, to become optimally horizontally aligned for transcription, when an infra-pressuromodulated gene becomes less than optimally horizontally aligned.
