# Supplementary material for: Horizontal alignment of 5′ -> 3′ intergene distance segment tropy with respect to the gene as the conserved basis for DNA transcription
Source: Future Sci OA. 2016 Dec 2;3(1):FSO160. doi: 10.4155/fsoa-2016-0070 (PMC5351715; doi:10.4155/fsoa-2016-0070)
Supplement: Supplementary file 1 [file fsoa-03-160-s1.doc]

Supplementary file 1 - Table S1. Selection of genes differentially overexpressed for analysis

| Gene Protein Name | Gene  Symbol | Ch  Locus | Strand  (+, -) | LEnC  Expression (au)  (Nelson *et al*, 2007) | BMEnC  Expression  (au)  (Nelson *et al*, 2007) | Over-expression  Cell Type | %  Over-expression |
| --- | --- | --- | --- | --- | --- | --- | --- |
| SorLA-1 (LR11) | *SORL1* | 11q24.1 | (+) | 6.80 | 2.46 | LEnC | 276 |
| Podoplanin (GP36) | *PDPN* | 1p36.21 | (+) | 11.81 | 4.65 | LEnC | 254 |
| B-cell translocation gene 1 (BTG1) | *BTG1* | 12q21.33 | (-) | 6.73 | 2.85 | LEnC | 236 |
| Hyaluran and proteoglycan link protein 1 (CRTL1) | *HAPLN1* | 5q14.3 | (-) | 10.92 | 4.67 | LEnC | 234 |
| Mannose Receptor (MRC1)  (CD206 Antigen) | *MRC1* | 10p12.33 | (+) | 11.63 | 5.48 | LEnC | 212 |
| Thiamine Monophosphatase | *ACPP* | 3q22.1 | (+) | 6.71 | 3.19 | LEnC | 210 |
| Transforming Growth Factor-alpha | *TGFA* | 2p13.3 | (-) | 7.91 | 3.79 | LEnC | 209 |
| S100 calcium binding protein A2 | *S100A2* | 1q21.3 | (-) | 8.15 | 6.87 | LEnC | 119 |
| PH domain and Leucine-rich  repeat protein phosphatase | *PHLPP* (*KIAA060*) | 18q21.33 | (+) | 9.26 | 8.19 | LEnC | 113 |
| Multidrug Resistant Protein 1  (MDR1; CD243; P-gp1) | *ABCB1* | 7q21.12 | (-) | 2.50 | 8.19 | BMEnC | 328 |
| E-Selectin (CD62E) | *SELE* | 1q24.2 | (-) | 3.27 | 9.97 | BMEnC | 305 |
| Proline-Rich Polypeptide 3 | *PRR3* | 6p21.33 | (+) | 2.60 | 6.92 | BMEnC | 266 |
| Cadherin 11, type 2 | *CDH11* | 16q21 | (-) | 4.13 | 10.38 | BMEnC | 251 |
| Dystrophin | *DMD* | Xp21.1 | (-) | 3.52 | 8.59 | BMEnC | 244 |
| Forkhead box P2 | *FOXP2* | 7q31.1 | (+) | 2.77 | 6.71 | BMEnC | 242 |
| Interferon alpha-inducible p27 | *IFI27* | 14q32.12 | (+) | 6.37 | 14.47 | BMEnC | 227 |
| S100 calcium binding protein A14 | *S100A14* | 1q21.3 | (-) | 5.06 | 7.23 | BMEnC | 143 |
| Zinc finger, CCHC domain containing 2 | *ZCCHC2* (*C18orf49*; *KIAA1744*) | 18q21.33 | (+) | 5.11 | 7.01 | BMEnC | 137 |
