# Supplementary material for: Horizontal alignment of 5′ -> 3′ intergene distance segment tropy with respect to the gene as the conserved basis for DNA transcription
Source: Future Sci OA. 2016 Dec 2;3(1):FSO160. doi: 10.4155/fsoa-2016-0070 (PMC5351715; doi:10.4155/fsoa-2016-0070)
Supplement: Supplementary file 2 [file fsoa-03-160-s2.pdf]

Table S2. Intergene distances with tropy pairing and paired gene overexpression tropy quotients (*$prpT_Q$*s) for respective episodes per gene category

Transcribed Location No.	Gene Symbol	Ch Locus	Strand (+, -)	Start from pter (base)	End from pter (base)	No. of Gene Bases	No. of Intergene Bases	3'->5' & 5'->3' Tropy Pairing	$prpT_Q$ (fract)	5' -> 3' Paired Order No.	OE Cell Type	No. of Episodes	Gene Category
1	PIR45272		(+)	119,585,965	119,585,995	31	**1,826**	**33**					
2	PIR32447		(+)	119,587,820	119,587,851	32	**12,259**	**32**					
3	LNC-CTD-2523D13.1.1-1		(+)	119,600,109	119,617,416	17,308	-8,992						
4	LOC105369520		(+)	119,608,423	119,659,530	51,108	50,323	31	0.003				
5	RNU6-1123P		(+)	119,656,311	119,656,416	106							
6	LOC105369525		(+)	119,709,852	119,720,221	10,370	**9,363**	**30**					
7	LOC102724301		(+)	119,729,583	119,739,623	10,041	**5,534**	**29**					
8	GC11P119746		(+)	119,745,156	119,748,429	3,274	**3,533**	**28**					
9	LOC101929156		(+)	119,751,961	119,770,074	18,114	**5,984**	**27**					
10	LNC-CTD-2523D13.1.1-4		(+)	119,776,057	119,784,117	8,061	**1,985**	**26**					
11	GC11P119786		(+)	119,786,101	119,786,667	567	16,633	25	0.21				
12	GC11P119803		(+)	119,803,299	119,804,749	1,451	4,040	24	0.39				
13	LNC-CTD-2523D13.1.1-5		(+)	119,808,788	119,810,697	1,910	**6,441**	**23**					
14	LNC-AP000679.2.1-2		(+)	119,817,137	119,821,850	4,714	**19,716**	**22**					
15	GC11P119841		(+)	119,841,565	119,848,488	6,924	48,973	21	0.12				
16	LNC-AP000679.2.1-1		(+)	119,897,460	119,908,000	10,541	-2,644						
17	LOC105369526		(+)	119,905,355	119,951,015	45,661	**75,781**	**20**					
18	LOC105369527		(+)	120,026,795	120,028,995	2,201	10,691	19	0.43				
19	LNC-AP000679.2.1-3		(+)	120,039,685	120,053,591	13,907	30,854	18	0.42				
20	LNC-POU2F3-2		(+)	120,084,444	120,088,221	3,778	40,282	17	0.13				
21	LOC105369529		(+)	120,128,502	120,139,424	10,923	29,121	16	*0.89*				
22	LOC729173		(+)	120,168,544	120,171,679	3,136	16,197	15	*0.90*				
23	LNC-TMEM136-1		(+)	120,187,875	120,189,896	2,022	**7,934**	**14**					
24	LNC-POU2F3-1		(+)	120,197,829	120,198,350	522	**12,418**	**13**					
25	OAF		(+)	120,210,767	120,230,332	19,566	6,309	12	0.10				
26	POU2F3		(+)	120,236,640	120,319,945	83,306	-741						
27	LNC-GRIK4-2		(+)	120,319,203	120,328,461	9,259	-3,830						
28	TMEM136		(+)	120,324,630	120,333,688	9,059	**2,719**	**11**					
29	ARHGEF12		(+)	120,336,406	120,489,936	153,531	-43,427						
30	LNC-AP002348.1-1		(+)	120,446,508	120,686,147	239,640	-174,400						
31	GRIK4		(+)	120,511,746	120,988,904	477,159	35,169	10	0.42				
32	TBCEL		(+)	121,024,072	121,090,775	66,704	**10,399**	**9**					
33	TECTA		(+)	121,101,173	121,191,493	90,321	26,606	8	0.43				
34	LNC-SC5DL-1		(+)	121,218,098	121,231,384	13,287	**5,955**	**7**					
35	LOC105369533		(+)	121,237,338	121,245,159	7,822	**41**	**6**					
36	LOC105369534		(+)	121,245,199	121,247,035	1,837	**33,162**	**5**					
37	LNC-SORL1-3		(+)	121,280,196	121,280,422	227	12,032	4	0.002				
38	SC5D		(+)	121,292,453	121,313,410	20,958	96,078	3	0.001				
39	GC11P121409		(+)	121,409,487	121,409,713	227	18,012	2					
40	LNC-SC5DL-2		(+)	121,427,724	121,429,482	1,759	**22,722**	**1**					
**41w/in**	**SORL1**	**11q24.1**	(+)	121,452,203	121,633,762	**181,560**					**LEnC**	**2**	**> 11,864**
42	**GC11P121607**		(+)	121,607,711	121,649,984	**42,274**							**≤ 265, 005**
43	GC11P121696		(+)	121,695,078	121,700,031	4,954	**45,095**	**1**	**0.50**	**0**			
44	GC11P121735		(+)	121,735,366	121,739,968	4,603	**35,336**	**2**	**0.51**	**1**			
45	LMC-SORL1-1		(+)	121,740,076	121,760,026	19,951	109	3					

46	LNC-SORL1-2	(+)	121,760,051	121,777,195	17,145	26	4		
47	GC11P121775	(+)	121,775,710	121,787,508	11,799	-1,484			
48	LNC-SORL1-4	(+)	121,959,224	121,959,664	441	**171,717**	**5**	**0.19**	**2**
49	GC11P121991	(+)	121,991,728	121,994,631	2,904	**32,065**	**6**	**0.001**	**3**
50	RNU6-256P	(+)	122,004,355	122,004,451	97	**9,725**	**7**	**0.61**	**4**
51	GC11P122016	(+)	122,016,012	122,016,700	689	11,562	8		
52	LNC-UBASH3B-2	(+)	122,051,515	122,053,688	2,174	**34,816**	**9**	**0.30**	**5**
53	LNC-UBASH3B-5	(+)	122,068,292	122,068,509	218	14,605	10		
54	GC11P122071	(+)	122,071,266	122,072,490	1,225	**2,758**	**11**	***0.99***	**6**
55	LNC-UBASH3B-4	(+)	122,073,146	122,074,158	1,013	657	12		
56	GC11P122088	(+)	122,088,516	122,088,956	441	**14,359**	**13**	***0.86***	**7**
57	GC11P122103	(+)	122,103,899	122,104,055	157	**14,944**	**14**	**0.53**	**8**
58	GC11P122118	(+)	122,118,572	122,139,937	21,366	14,518	15		
59	LNC-UBASH3B-2	(+)	122,165,898	122,179,274	13,377	25,962	16		
60	GC11P122184	(+)	122,184,487	122,184,757	271	5,214	17		
61	GC11P122197	(+)	122,197,584	122,197,801	218	12,828	18		
62	GC1P122202	(+)	122,202,438	122,203,450	1,013	4,638	19		
63	LNC-UBASH3B-1	(+)	122,282,462	122,289,181	6,720	**79,013**	**20**	***0.96***	**9**
64	ENSG00000255219	(+)	122,295,190	122,308,019	12,830	6,010	21		
65	GC11P122331	(+)	122,330,676	122,336,982	6,307	**22,658**	**22**	***0.87***	**10**
66	GC11P122347	(+)	122,347,586	122,349,070	1,485	**10,605**	**23**	**0.61**	**11**
67	GC11P122350	(+)	122,350,656	122,351,412	757	1,587	24		
68	GC11P122354	(+)	122,354,864	122,356,440	1,577	3,453	25		
69	GC11P122358	(+)	122,358,472	122,360,494	2,023	**2,033**	**26**	***0.98***	**12**
70	GC11P122388	(+)	122,388,924	122,395,791	6,868	**28,431**	**27**	**0.21**	**13**
71	GC11P122407	(+)	122,406,778	122,408,150	1,373	**10,988**	**28**	**0.32**	**14**
72	GC11P122418	(+)	122,418,214	122,420,847	2,634	**10,065**	**29**	**0.55**	**15**
73	GC11P122450	(+)	122,449,207	122,449,331	125	**28,361**	**30**	**0.33**	**16**
74	RNU4ATAC10P	(+)	122,449,210	122,449,331	122				
75	GC11P122453	(+)	122,449,496	122,454,841	5,346	166	31		
76	GC11P122452/RNU4ATAC5P	(+)	122,451,472	122,451,599	128				
77	LNC-UBASH3B-3	(+)	122,489,559	122,489,982	424	**34,719**	**32**	**0.35**	**17**
78	GC11P122524	(+)	122,524,522	122,580,201	55,680	**72,924**	**33**	**0.03**	
79	GC01P011758	(+)	11,755,863	11,758,432	2,570	**3,356**	**42**		
80	C1orf167	(+)	11,761,787	11,789,585	27,799	**6,603**	**41**		
81	LNC-C1orf187-4	(+)	11,796,187	11,806,729	10,543	-632			
82	CLCN6	(+)	11,806,096	11,843,144	37,049	-2,824			
83	NPPA-AS1/LNC-C1orf167-2	(+)	11,840,319	11,849,642	9,324	28,128	40	0.06	
84	LOC390997	(+)	11,877,769	11,880,758	2,990	**9,149**	**39**		
85	LNC-C1orf167-3	(+)	11,889,906	11,894,365	4,460	6,012	38	0.84	
86	LNC-NPPA-AS1	(+)	11,900,376	11,908,136	7,761	-14			
87	LOC105376742	(+)	11,908,121	11,914,327	6,207	19,879	37	0.42	
88	PLOD1	(+)	11,934,205	11,975,542	41,338	**4,640**	**36**		
89	MFN2	(+)	11,980,181	12,013,515	33,335	**5,728**	**35**		
90	MIIP	(+)	12,019,242	12,032,049	12,808	**13,361**	**34**		
91	GC01P012045	(+)	12,045,409	12,126,583	81,175	-63,252			
92	TNFRSF8	(+)	12,063,330	12,144,207	80,878	22,737	33	0.67	
93	MIR7846	(+)	12,166,943	12,167,038	96	-88			

94	TNFRSF1B		(+)	12,166,949	12,209,228	42,280	20,812	32	0.47				
95	VPS13D		(+)	12,230,039	12,512,047	282,009	13,234	31	*0.76*				
96	LOC105376744		(+)	12,525,280	12,528,868	3,589	**3,694**	**30**					
97	GC01P012533		(+)	12,532,561	12,535,780	3,220	**4,386**	**29**					
98	PIR43536		(+)	12,540,165	12,540,191	27	17,090	28	0.88				
99	LNC-AADACL4-1/PIR41325		(+)	12,557,280	12,557,327	48	30,160	27	0.02				
100	LNC-VPS13D-1		(+)	12,587,486	12,589,505	2,020	55,043	26	0.02				
101	AADACL4		(+)	12,644,547	12,667,086	22,540	25,824	25	0.71				
102	ENSG00000226166		(+)	12,692,909	12,693,020	112	**23,096**	**24**					
103	AADACL3		(+)	12,716,115	12,728,759	12,645	17,428	23	0.35				
104	C1orf158		(+)	12,746,186	12,763,699	17,514	-475						
105	PRAMEF12		(+)	12,763,223	12,777,906	14,684	13,492	22	0.61				
106	PRAMEF1		(+)	12,791,397	12,796,628	5,232	9,540	21	0.11				
107	LNC-C1orf158-1		(+)	12,806,167	12,823,847	17,681	28,965	20	0.01				
108	GC01P012854		(+)	12,852,811	12,855,992	3,182	**1,095**	**19**					
109	PRAMEF2		(+)	12,857,086	12,861,909	4,824	54,702	18	0.02				
110	PRAMEF7		(+)	12,916,610	12,920,482	3,873	**2,073**	**17**					
111	RNU6-1072P		(+)	12,922,554	12,922,660	107	**3,503**	**16**					
112	PRAMEF29P		(+)	12,926,162	12,928,882	2,721	76,167	15	0.46				
113	PRAMEF22		(+)	13,005,048	13,005,624	577	23,716	14	0.32				
114	PRAMEF28		(+)	13,029,339	13,032,199	2,861	**6,001**	**13**					
115	GC01P013038		(+)	13,038,199	13,039,061	863	21,793	12	0.13				
116	PRAMEF25		(+)	13,060,853	13,078,038	17,186	49,032	11	0.18				
117	PRAMEF36P		(+)	13,127,069	13,132,274	5,206	14,504	10	0.41				
118	LNC-PRAMEF22-1		(+)	13,146,777	13,147,256	480	176,784	9	0.20				
119	ENSG00000228229		(+)	13,324,039	13,324,518	480	65,115	8	*0.78*				
120	PRAMEF17		(+)	13,389,632	13,392,629	2,998	17,822	7	*0.82*				
121	PRAMEF20		(+)	13,410,450	13,421,328	10,879	8,307	6	0.18				
122	LNC-PRAMEF9-1		(+)	13,429,634	13,430,113	480	57,465	5	0.39				
123	LOC102724856		(+)	13,487,577	13,493,138	5,562	20,083	4	0.46				
124	ENSG00000259961		(+)	13,513,220	13,516,270	3,051	6,749	3	0.46				
125	LNC-PRAMEF16-1		(+)	13,523,018	13,523,497	480	31,505	2	0.22				
126	BRWD1P1		(+)	13,555,001	13,555,694	694	**27,772**	**1**					
**127**	**PDPN**	**01p36.21**	(+)	13,583,465	13,617,957	**34,493**					**LEnC**	**2**	**> 11,864**
128	LNC-PRAMEF15-1		(+)	13,650,431	13,650,910	480	**32,475**	**1**	***0.86***	**0**			**≤ 265, 005**
129	GC01P013657		(+)	13,657,926	13,657,961	36	7,017	2					
130	PIR40018		(+)	13,661,085	13,661,111	27	3,125	3					
131	LOC105378612		(+)	13,670,248	13,674,725	4,478	9,138	4					
132	GC01P013696		(+)	13,696,914	13,698,781	1,868	22,190	5					
133	PRDM2		(+)	13,700,240	13,825,079	124,840	1,460	6					
134	LNC-PDPN-1		(+)	13,839,715	13,842,765	3,051	14,637	7					
135	KAZN		(+)	13,893,515	15,118,048	1,224,534	50,751	8					
136	TMEM51		(+)	15,152,532	15,220,480	67,949	34,485	9					
137	LOC100421184		(+)	15,226,364	15,227,549	1,186	5,885	10					
138	FHAD1		(+)	15,236,602	15,400,283	163,682	9,054	11					
139	GCO1P015399		(+)	15,399,726	15,407,022	7,297	-556						
140	EFHD2		(+)	15,409,895	15,430,343	20,449	2,874	12					
141	CTRC		(+)	15,438,439	15,449,247	10,809	**8,097**	**13**	**0.74**	**1**			
142	CELA2A		(+)	15,456,728	15,472,091	15,364	7,482	14					
143	CELA2B		(+)	15,465,909	15,491,400	25,492	-6,181						

144	DNAJC16	(+)	15,526,813	15,592,379	65,567	35,414	15		
145	GC01P015603	(+)	15,603,740	15,605,536	1,797	**11,362**	**16**	**0.31**	**2**
146	CHCHD2P6		15,604,528	15,605,318	791				
147	LNC-TMEM51-1	(+)	15,613,541	15,616,137	2,597	**8,006**	**17**	**0.26**	**3**
148	DDI2	(+)	15,617,458	15,669,044	51,587	1,322	18		
149	PLEKHM2	(+)	15,684,267	15,734,769	50,503	**15,224**	**19**	**0.07**	**4**
150	SLC25A34	(+)	15,735,088	15,741,396	6,309	320	20		
151	TMEM82	(+)	15,742,422	15,747,982	5,561	1,027	21		
152	FBLIM1	(+)	15,756,171	15,786,594	30,424	8,190	22		
153	RPL12P14	(+)	15,792,727	15,793,346	620	6,134	23		
154	RPS16P1	(+)	15,828,212	15,828,733	522	**34,867**	**24**	**0.66**	**5**
155	GC01P015847	(+)	15,847,101	15,945,538	98,438	18,369	25		
156	GC01P015946	(+)	15,946,838	15,947,784	947	1,301	26		
157	LOC101928531	(+)	15,948,508	15,954,649	6,142	725	27		
158	ENSG00000234607	(+)	15,969,632	15,970,194	563	14,984	28		
159	TBC1D3P6	(+)	15,976,131	15,997,206	21,076	**5,938**	**29**	**0.74**	**6**
160	C1orf64	(+)	16,004,236	16,008,807	4,572	**7,031**	**30**	**0.53**	**7**
161	CLCNKA	(+)	16,018,875	16,034,050	15,176	10,069	31		
162	CLCNKB	(+)	16,043,736	16,057,326	13,591	9,687	32		
163	LNC-SLC25A34-1	(+)	16,072,670	16,073,595	926	15,345	33		
164	PIR52803	(+)	16,090,594	16,090,623	30	**17,000**	**34**	***0.79***	**8**
165	GC01P016103/LNC-TMEM82-1	(+)	16,103,231	16,111,333	8,103	**12,609**	**35**	**0.45**	**9**
166	LNC-SPEN-2	(+)	16,145,920	16,146,866	947	**34,588**	**36**	**0.13**	**10**
167	ENSG00000227959	(+)	16,155,211	16,157,329	2,119	8,346	37		
168	LOC101927479	(+)	16,155,001	16,168,216	13,216	-2,327			
169	GC01P016173	(+)	16,173,273	16,176,219	2,947	5,058	38		
170	ARHGEF19-AS1	(+)	16,197,854	16,198,357	504	**21,636**	**39**	**0.42**	**11**
171	LNC-FBLIM1-1	(+)	16,199,997	16,203,206	3,210	1,641	40		
172	GC01P016210	(+)	16,210,969	16,217,507	6,539	**7,764**	**41**	***0.85***	
173	PIR39576	(+)	16,244,010	16,244,037	28	**26,504**	**42**	**0.13**	

174	LOC718084	(-)	89,010,681	89,019,692	9,012	**28,496**	**31**	**0.36**	
175	ENSG00000257156	(-)	89,048,187	89,309,553	261,367	37,680	30		
176	RNU7-120P	(-)	89,282,223	89,282,285	63				
177	DUSP6	(-)	89,347,232	89,353,271	6,040	1,155	29		
178	PIR57068	(-)	89,354,425	89,354,451	27	**50,008**	**28**	**0.01**	**16**
179	LNC-DUSP6-2	(-)	89,404,458	89,413,469	9,012	28,496	27		
180	LNC-DUSP6-1	(-)	89,441,964	89,703,330	261,367	-115,280			
181	ATP2B1	(-)	89,588,049	89,709,353	121,305	**104,143**	**26**	**0.03**	**15**
182	LNC-POC1B-1/LNC-GALNT4-1	(-)	89,813,495	89,919,192	105,698	230	25		
183	BRWD1P2	(-)	89,919,421	89,919,776	356	**13,218**	**24**	**0.04**	**14**
184	LNC-POC1B-GALNT4-2	(-)	89,932,993	89,941,782	8,790	11,384	23		
185	GC12M089953	(-)	89,953,165	90,025,324	72,160	**118,464**	**22**	**0.36**	**13**
186	GC12M090143	(-)	90,143,787	90,145,074	1,288	**168,141**	**21**	**0.02**	**12**
187	LNC-ATP2B1-1	(-)	90,313,214	90,313,553	340	**215,285**	**20**	**0.11**	**11**
188	LNC-ATP2B1-2	(-)	90,528,837	90,537,544	8,708	**33,472**	**19**	***0.94***	**10**
189	LNC-ATP2B1-3	(-)	90,571,015	90,587,981	16,967	29,779	18		
190	ENSG00000257995	(-)	90,617,759	90,622,822	5,064	15,297	17		
191	LNC-ATP2B1-4	(-)	90,638,118	90,647,093	8,976	**98,765**	**16**	***0.91***	**9**

192	GC12M090745		(-)	90,745,857	90,785,864	40,008	-24,060						
193	LOC105369895		(-)	90,761,803	90,809,151	47,349	**95,217**	**15**	**0.04**	**8 (NC)**			
194	LNC-C12orf12-4		(-)	90,904,367	90,905,544	1,178	79	14					
195	CCER1		(-)	90,905,622	90,955,176	49,555	8,504	13					
196	EPYC		(-)	90,963,679	91,007,906	44,228	**3,631**	**12**	**0.46**	**7**			
197	LNC-C12orf12-3		(-)	91,011,536	91,016,599	5,064	**33,893**	**11**	**0.34**	**6**			
198	KERA		(-)	91,050,491	91,058,354	7,864	44,276	10					
199	LUM		(-)	91,102,629	91,111,831	9,203	**28,654**	**9**	**0.60**	**5**			
200	DCN		(-)	91,140,484	91,183,123	42,640	-27,433						
201	LNC-C12orf12-2		(-)	91,155,689	91,200,553	44,865	28,408	8					
202	LOC105369898		(-)	91,228,960	91,365,436	136,477	199	7					
203	LOC105369896		(-)	91,365,634	91,399,373	33,740	7,564	6					
204	PIR55198		(-)	91,406,936	91,406,967	32	**23,459**	**5**	**0.61**	**4**			
205	GC12M091431		(-)	91,430,425	91,522,727	92,303	-36,824						
206	LOC105369899		(-)	91,485,902	91,633,824	147,923	**158,625**	**4**	**0.45**	**3**			
207	LNC-DCN-1		(-)	91,792,448	91,821,793	29,346	**49,330**	**3**	**0.03**	**2**			
208	ENSG00000258224		(-)	91,871,122	91,871,230	109	**47,137**	**2**	**0.09**	**1**			
209	LOC105369901		(-)	91,918,366	91,961,098	42,733	**23,879**	**1**	**0.02**	**0**			
210	**LINC01619**		(-)	91,984,976	92,142,914	**157,939**							
**211w/in**	**BTG1**	**12q21.33**	(-)	92,140,278	92,145,897	**5,620**					**LEnC**	**2**	**> 11,864**
212	LOC101930023		(-)	92,146,489	92,363,858	217,370	**593**	**1**					**≤ 265, 005**
213	LNC-LINCO1619		(-)	92,367,962	92,536,690	168,729	**4,105**	**2**					
214	LNC-RP11-24B21.1.1-1		(-)	92,537,962	92,539,009	1,048	**1,273**	**3**					
215	LNC-BTG1-2		(-)	92,610,794	92,611,310	517	**71,786**	**4**					
216	LNC-BTG1-4		(-)	92,625,698	92,655,730	30,033	**14,389**	**5**					
217	LNC-CLLU1OS-1		(-)	92,641,531	92,757,608	116,078	-14,198						
218	EEA1		(-)	92,770,637	92,929,331	158,695	13,030	6	0.58				
219	GC12M092931		(-)	92,931,510	92,932,667	1,158	2,180	7	0.09				
220	ENSG00000257322		(-)	93,003,415	93,215,679	212,265	70,749	8	0.40				
221	LOC643339		(-)	93,003,758	93,377,736	373,979	-211,920						
222	RPL41P5		(-)	93,083,293	93,083,716	424							
223	GC12M093281		(-)	93,280,370	93,412,196	131,827	-97,365						
224	UBE2N		(-)	93,405,673	93,443,445	37,773	-6,522						
225	ENSG00000200011		(-)	93,460,726	93,460,826	101	**17,282**	**9**					
226	LNC-UBE2N-1		(-)	93,519,828	93,532,867	13,040	59,003	10	*0.75*				
227	LNC-EEA1-6		(-)	93,529,560	93,530,895	1,336	-3,306						
228	SOCS2-AS1		(-)	93,542,463	93,571,768	29,306	**11,569**	**11**					
229	LNC-UBE2N-1		(-)	93,573,434	93,771,512	198,079	**1,667**	**12**					
230	LNC-UBE2N-4		(-)	93,799,449	93,801,099	1,651	27,938	13	0.30				
231	LOC105369911		(-)	93,894,965	93,943,606	48,642	93,867	14	0.001				
232	LNC-SOCS2-AS1		(-)	93,936,239	93,965,544	29,306	-7,366						
233	LOC105369912		(-)	93,969,696	94,012,119	42,424	**4,153**	**15**					
234	RN7SKP263		(-)	94,008,739	94,009,047	309							
235	LNC-UBE2N-3		(-)	94,101,567	94,131,599	30,033	**89,449**	**16**					
236	ENSG00000258035		(-)	94,168,006	94,186,044	18,039	36,408	17	0.42				
237	CEP83		(-)	94,265,652	94,459,988	194,337	79,609	18	0.37				
238	RBMS2P1		(-)	94,423,468	94,425,050	1,583							
239	ENSG00000257400		(-)	94,491,546	94,496,442	4,897	**31,559**	**19**					
240	GC12M094521/LOC400061		(-)	94,520,433	94,521,980	1,548	**23,992**	**20**					
241	GC12M094524		(-)	94,524,787	94,525,002	216	**2,808**	**21**					

242	TMCC3		(-)	94,567,124	94,650,562	83,439	**42,123**	**22**					
243	GC12M094666/LNC-RP11-1105G2.3.1-1		(-)	94,665,314	94,670,971	5,658	14,753	23	*0.77*				
244	LNC-RP11-1105G2.3.1-5		(-)	94,671,534	94,676,620	5,087	**564**	**24**					
245	LNC-RP11-1105G2.3.1-6		(-)	94,702,603	94,706,954	4,352	25,984	25	0.01				
246	LNC-RP11-1105G2.3.1-7		(-)	94,709,809	94,713,570	3,762	**2,856**	**26**					
247	LNC-RP11-1105G2.3.1-8		(-)	94,794,680	94,796,112	1,433	81,111	27	0.35				
248	LNC-RP11-1105G2.3.1-9		(-)	94,796,555	94,801,873	5,319	**444**	**28**					
249	GC12M094805		(-)	94,805,586	94,816,144	10,559	3,714	29	0.31				
250	GC12M094870		(-)	94,869,258	94,910,545	41,288	53,115	30	0.71				
251	NDUFA12/LNC-CCDC41-2		(-)	94,897,055	95,009,927	112,873	-13,489						
252	NR2C1		(-)	95,020,229	95,073,703	53,475	**10,303**	**31**					

253	SSBP2		(-)	81,413,021	81,751,797	338,777	**89,885**	**33**	***0.81***				
254	LNC-RPS23-2		(-)	81,841,681	81,842,129	449	10,175	32					
255	LNC-RPS23-4		(-)	81,852,303	81,852,514	212	30,082	31					
256	LNC-RPS23-3		(-)	81,882,595	81,883,230	636	**126,161**	**30**	**0.22**				
257	PPIAP11		(-)	82,009,390	82,010,140	751	**62,916**	**29**	**0.60**	**12**			
258	ATG10-AS1		(-)	82,073,055	82,073,702	648	**60,956**	**28**	**0.01**	**11**			
259	GC05M082134		(-)	82,134,657	82,175,481	40,825	6,413	27					
260	LNC-TMEM167A-7		(-)	82,181,893	82,191,527	9,635	**23,669**	**26**	**0.45**	**10**			
261	LNC-TMEM167A-3		(-)	82,215,195	82,216,938	1,744	15,932	25					
262	GC05M082232		(-)	82,232,869	82,233,415	547	2,863	24					
263	LNC-TMEM167A-2		(-)	82,236,277	82,236,802	526	21,616	23					
264	GC05M082258		(-)	82,258,417	82,279,560	21,144	28,545	22					
265	LNC-TMEM167A-1		(-)	82,308,104	82,308,928	825	7,957	21					
266	LNC-TMEM167A-6		(-)	82,316,884	82,317,185	302	**28,011**	**20**	***0.82***	**9**			
267	LNC-TMEM167A-5		(-)	82,345,195	82,346,783	1,589	1,885	19					
268	LNC-TMEM167A-4		(-)	82,348,667	82,373,682	25,016	**172,181**	**18**	**0.31**	**8**			
269	ENSG00000248393		(-)	82,545,862	82,546,310	449	10,175	17					
270	GC05M082556		(-)	82,556,484	82,556,695	212	19,046	16					
271	LNC-TMEM167A-8		(-)	82,575,740	82,576,463	724	10,314	15					
272	ENSG00000248870		(-)	82,586,776	82,587,411	636	**113,009**	**14**	**0.71**	**7**			
273	GC05M082700		(-)	82,700,419	82,702,470	2,052	**106,831**	**13**	**0.09**	**6**			
274	LNC-HAPLN1-2		(-)	82,809,300	82,858,133	48,834	-20,836						
275	LNC-HAPLN1-1		(-)	82,837,296	82,877,139	39,844	36,229	12					
276	LOC105379051		(-)	82,913,367	82,924,327	10,961	**12,725**	**11**	***0.76***	**5**			
277	LNC-EDIL3-6		(-)	82,937,051	82,937,306	256	3,153	10					
278	ENSG00000249857		(-)	82,940,458	82,940,983	526	70,459	9					
279	LOC105379052		(-)	83,011,441	83,023,190	11,750	1,804	8					
280	GC05M083024		(-)	83,024,993	83,037,368	12,376	**12,009**	**7**	**0.17**	**4**			
281	ENSG00000271862		(-)	83,049,376	83,050,964	1,589	1,883	6					
282	TMEM167A		(-)	83,052,846	83,077,863	25,018	**93,123**	**5**	***0.84***	**3**			
283	LNC-EDIL3-4		(-)	83,170,985	83,171,640	656	**102,256**	**4**	**0.23**	**2**			
284	GC05M083273		(-)	83,273,895	83,284,277	10,383	-4,356						
285	COQ19BP2		(-)	83,279,920	83,280,644	725	**121,835**	**3**	**0.53**	**1**			
286	LNC-HAPLN1-3		(-)	83,402,478	83,477,060	74,583	29,981	2					
287	LOC105379054		(-)	83,507,040	83,562,372	55,333	-31,019						
288	VCAN-AS1		(-)	83,531,352	83,581,320	49,969	**56,486**	**1**	**0.44**	**0**			
**289**	**HAPLN1**	**5q14.3**	(-)	83,637,805	83,721,613	**83,809**					**LEnC**	**2**	**> 11,864**

290	RPL13AP14	(-)	83,746,388	83,746,989	602	**24,776**	**1**		**≤ 265, 005**
291	LOC100421623	(-)	83,875,166	83,875,821	656	128,178	2	0.23	
292	EDIL3	(-)	83,940,554	84,384,867	444,314	**64,734**	**3**		
293	LNC-EDIL3-1	(-)	84,408,160	84,408,574	415	**23,294**	**4**		
294	LNC-EDIL3-9	(-)	84,486,447	84,486,648	202	**77,874**	**5**		
295	LNC-EDIL3-10	(-)	84,505,878	84,506,721	844	19,231	6	0.10	
296	LNC-EDIL3-2	(-)	84,508,776	84,509,432	657	**2,056**	**7**		
297	LNC-EDIL3-11	(-)	84,533,499	84,533,892	394	24,068	8	0.07	
298	LNC-EDIL3-3	(-)	84,715,846	84,716,269	424	181,955	9	0.39	
299	LNC-EDIL3-12	(-)	84,839,926	84,915,815	75,890	123,658	10	0.03	
300	LNC-EDIL3-13	(-)	84,925,509	84,943,631	18,123	9,695	11		
301	LOC100289244	(-)	84,999,618	85,000,315	698	55,988	12	0.65	
302	GC05M085010	(-)	85,010,268	85,032,669	22,402	**9,954**	**13**		
303	ENSG00000249349	(-)	85,112,342	85,112,756	415	**79,674**	**14**		
304	RBBP4P6	(-)	85,190,627	85,190,974	348	77,872	15	0.13	
305	ENSG00000250253	(-)	85,210,060	85,210,903	844	19,087	16	*0.998*	
306	LOC101060076	(-)	85,210,038	85,216,823	6,786	-864			
307	ENSG00000248170	(-)	85,237,681	85,238,074	394	20,859	17	0.49	
308	LOC101929321	(-)	85,291,405	85,304,187	12,783	**53,332**	**18**		
309	GC05M085340	(-)	85,340,694	85,396,965	56,272	36,508	19	0.05	
310	ENSG00000248667	(-)	85,420,028	85,420,451	424	**23,064**	**20**		
311	ENSG00000215953	(-)	85,528,044	85,528,134	91	107,594	21	0.07	
312	PIR37251	(-)	85,587,695	85,587,721	27	59,562	22	0.48	
313	LNC-AC008394.1-9	(-)	85,858,379	85,866,294	7,916	270,659	23	0.08	
314	LOC105379063	(-)	86,010,709	86,031,103	20,395	144,416	24	0.02	
315	PIR59371	(-)	86,272,716	86,272,744	29	241,614	25	0.07	
316	GC05M086283	(-)	86,283,339	86,283,369	31	**10,596**	**26**		
317	GC05M086306/ENSG00000280009	(-)	86,305,176	86,306,149	974	21,808	27	0.29	
318	GC05M086307	(-)	86,306,596	86,317,384	10,789	**448**	**28**		
319	LOC105379064	(-)	86,355,018	86,429,992	74,975	**37,635**	**29**		
320	PIR44300	(-)	86,457,741	86,457,769	29	**27,750**	**30**		
321	LNC-AC008394.1-3	(-)	86,501,225	86,535,962	34,738	43,457	31	0.69	
322	LNC-AC008394.1-4	(-)	86,590,008	86,590,290	283	54,047	32	0.19	
323	RNU6-804P	(-)	86,663,144	86,663,250	107	**72,855**	**33**		

324	PIR60156	(+)	14,910,000	14,910,027	28	**18,546**	**48**	
325	LNC-HSPA14-2	(+)	14,928,572	14,928,947	376	**4,099**	**47**	
326	GC10P014933	(+)	14,933,045	14,957,078	24,034	-2,849		
327	MEIG1	(+)	14,954,228	14,988,050	33,823	**2,546**	**46**	
328	LNC=MEIG1-2	(+)	14,990,595	14,991,170	576	**10,269**	**45**	
329	GC10P015013/LNC-MEIG1-1	(+)	15,001,438	15,030,049	28,612	**2,179**	**44**	
330	OLAH	(+)	15,032,227	15,073,852	41,626	17,871	43	0.30
331	LNC-RPP38-4/RPP38/LOC105376431	(+)	15,091,722	15,140,192	48,471	-955		
332	LNC-RPP38-1	(+)	15,139,236	15,181,817	42,582	26,082	42	0.60
333	LOC105376433	(+)	15,207,898	15,241,767	33,870	**11,897**	**41**	
334	LNC-RPP38-5	(+)	15,253,663	15,256,604	2,942	22,888	40	0.46
335	LNC-RPP38-3	(+)	15,279,491	15,283,766	4,276	164,898	39	0.20
336	GC10P015448	(+)	15,448,663	15,466,196	17,534	152,050	38	0.08
337	GC10P015618	(+)	15,618,245	15,637,887	19,643	99,887	37	0.05

338	LOC101928678		(+)	15,737,773	15,850,013	112,241	**11,834**	**36**					
339	GC10P015861/GC10P015862		(+)	15,861,846	15,863,066	1,221	40,780	35	0.72				
340	LNC-PTER-6		(+)	15,903,845	15,904,481	637	**85**	**34**					
341	LNC-PTER-5		(+)	15,904,565	15,905,065	501	100,744	33	0.29				
342	ENSG00000252537		(+)	16,005,808	16,005,923	116	119,629	32	0.36				
343	GC10P016125		(+)	16,125,551	16,126,613	1,063	17,643	31	0.01				
344	GC10P016144		(+)	16,144,255	16,146,576	2,322	**2,560**	**30**					
345	GC10P016147		(+)	16,149,135	16,151,084	1,950	**7,642**	**29**					
346	GC10P016158		(+)	16,158,725	16,174,390	15,666	**59,458**	**28**					
347	GC10P016233		(+)	16,233,847	16,235,100	1,254	**41,818**	**27**					
348	LOC102724039		(+)	16,276,917	16,295,858	18,942	**24,843**	**26**					
349	LNC-PTER-2		(+)	16,320,700	16,337,857	17,158	22,850	25	0.05				
350	LNC-PTER-4		(+)	16,360,706	16,377,863	17,158	59,081	24	0.06				
351	PTER		(+)	16,436,943	16,513,745	76,803	11,086	23	0.45				
352	RNU2-18P		(+)	16,476,003	16,476,164	162							
353	LOC101928701		(+)	16,524,830	16,539,768	14,939	**29,747**	**22**					
354	LNC-PTER-1		(+)	16,569,514	16,581,773	12,260	**27,899**	**21**					
355	LNC-PTER-3		(+)	16,609,671	16,621,779	12,109	**3,308**	**20**					
356	GC10P016625		(+)	16,625,086	16,625,103	18	14,619	19	*0.85*				
357	GC10P016639		(+)	16,639,721	16,642,491	2,771	124,745	18	0.03				
358	ENSG00000275001		(+)	16,767,235	16,767,416	182	50,297	17	0.42				
359	LOC105376435		(+)	16,817,712	16,821,245	3,534	22,403	16	0.20				
360	GC10P016843		(+)	16,843,647	16,904,650	61,004	235,271	15	*0.83*				
361	GC10P017139		(+)	17,139,920	17,140,009	90	87,927	14	0.68				
362	VIM		(+)	17,227,935	17,237,593	9,659	33,276	13	*0.78*				
363	LNC-STAM-4		(+)	17,270,868	17,271,749	882	115,188	12	0.09				
364	ST8SIA6-AS1		(+)	17,386,936	17,413,503	26,568	23,788	11	0.33				
365	LOC105376436		(+)	17,437,290	17,453,546	16,257	-24,622						
366	LNC-ST8SIA6-AS1		(+)	17,428,923	17,455,502	26,580	**13,440**	**10**					
367	LNC-VIM-2		(+)	17,468,941	17,490,287	21,347	**6,152**	**9**					
368	LNC-STAM-5		(+)	17,496,438	17,497,814	1,377	67,291	8	0.11				
369	LNC-STAM-3		(+)	17,565,104	17,577,586	12,483	-300						
370	PRPF38AP2		(+)	17,577,285	17,578,215	931	24,696	7	0.64				
371	ENSG00000280126		(+)	17,602,910	17,603,127	218	16,235	6	0.01				
372	LNC-STAM-2		(+)	17,619,361	17,620,211	851	**21,082**	**5**					
373	GC10P017641		(+)	17,641,292	17,642,931	1,640	**1,195**	**4**					
374	STAM		(+)	17,644,125	17,716,824	72,700	**9,961**	**3**					
375	LNC-TMEM236-1		(+)	17,726,784	17,730,156	3,373	**22,055**	**2**					
376	TMEM236		(+)	17,752,210	17,800,887	48,678	**8,457**	**1**					
**377**	**MRC1**	**10p12.33**	(+)	17,809,343	17,911,170	**101,828**				**LEnC**	**LEnC**	**2**	**> 11,864**
378	SLC39A12		(+)	17,951,839	18,043,292	91,454	**40,670**	**1**	**0.21**	**0**			**≤ 265, 005**
379	CACNB2		(+)	18,140,677	18,541,869	401,193	**97,386**	**2**	**0.23**	**1**			
380	ARL5B		(+)	18,659,335	18,681,639	22,305	**117,467**	**3**	**0.08**	**2**			
381	LOC105376440		(+)	18,710,294	18,836,471	126,178	**28,656**	**4**	**0.04**	**3**			
382	AIFM1P1		(+)	18,735,675	18,738,829	3,155							
383	ENSG00000234813		(+)	18,940,501	18,940,953	453	**104,031**	**5**	**0.20**	**4**			
384	LNC-ARL5B-1		(+)	18,941,165	18,943,952	2,788	213	6					
385	PIR61812		(+)	18,959,749	18,959,779	31	15,798	7					
386	LNC-CACNB2-1		(+)	18,967,049	18,970,567	3,519	7,271	8					
387	LNC-ARL5B-4		(+)	19,007,055	19,010,573	3,519	**36,489**	**9**	**0.17**	**5**			

388	LNC-ARL5B-5	(+)	19,024,674	19,027,560	2,887	14,102	10	0.95	6
389	LNC-ARL5B-2	(+)	19,035,362	19,036,782	1,421	7,803	11		
390	MALRD1	(+)	19,046,931	19,790,401	743,471	10,150	12		
391	UBE2V2P1	(+)	19,051,455	19,052,172	718				
392	HMGN1P20	(+)	19,489,101	19,489,400	300				
393	MTND2P16	(+)	19,747,822	19,748,789	968				
394	PLXDC2	(+)	19,816,227	20,289,856	473,630	25,827	13		
395	AMD1P1	(+)	20,350,049	20,351,100	1,052	60,194	14		
396	ENSG00000270727	(+)	20,547,171	20,547,422	252	196,072	15		
397	MIR4675	(+)	20,551,970	20,552,046	77	4,549	16		
398	LNC-PLXDC2-11	(+)	20,573,293	20,578,649	5,357	21,248	17		
399	GC10P020582	(+)	20,582,911	20,584,691	1,781	4,263	18		
400	LOC105376442	(+)	20,597,130	20,610,771	13,642	12,440	19		
401	LNC-PLXDC2-3	(+)	20,638,978	20,640,029	1,052	28,208	20	0.12	7
402	MTND1P21	(+)	20,804,281	20,804,580	300	164,253	21	0.17	8
403	LNC-PLXDC2-5	(+)	20,836,100	20,836,351	252	31,521	22	0.94	9
404	ENSG00000230112	(+)	20,841,375	20,841,560	186	5,025	23		
405	LOC102725112	(+)	20,845,375	20,884,942	39,568	3,816	24		
406	LNC-PLXDC2-1	(+)	20,886,074	20,898,900	12,827	1,133	25		
407	LNC-PLXDC2-8	(+)	20,925,994	20,938,818	12,825	27,095	26	0.92	10
408	EIF4BP2	(+)	21,028,983	21,030,793	1,811	90,166	27	0.46	11
409	LNC-PLXDC2-9	(+)	21,093,222	21,093,506	285	62,430	28	0.95	12
410	NPM1P30	(+)	21,110,682	21,113,703	3,022	17,177	29	0.44	13
411	LNC-PLXDC2-10	(+)	21,134,588	21,173,848	39,261	20,886	30	0.12	14
412	NEBL-AS1	(+)	21,173,990	21,175,048	1,059	143	31		
413	ENSG00000228860	(+)	21,217,891	21,218,279	389	42,844	32		
414	LUZP4P1	(+)	21,247,752	21,248,095	344	29,474	33		
415	RNU6-15P	(+)	21,321,961	21,322,067	107	73,867	34	0.001	15
416	GC10P021351	(+)	21,351,387	21,351,418	32	29,321	35		
417	RNMTL1P1	(+)	21,394,016	21,394,592	577	42,599	36	0.28	16
418	LNC-MLLT10-6	(+)	21,399,611	21,402,317	2,707	5,020	37		
419	LOC105376445	(+)	21,414,787	21,418,654	3,868	12,471	38		
420	ENSG00000278650	(+)	21,452,197	21,452,412	216	33,544	39		
421	LNC-NEBL-AS1	(+)	21,462,919	21,463,977	1,059	10,508	40		
422	GC10P021482/ENSG00000199222	(+)	21,481,715	21,481,817	103	17,739	41	0.67	17
423	GC10P021497	(+)	21,497,404	21,497,751	348	15,588	42		
424	LNC-MLLT10-5	(+)	21,503,196	21,504,644	1,449	5,446	43		
425	MLLT10	(+)	21,524,675	21,742,633	217,959	20,032	44	0.11	18
426	RN7SKP219	(+)	21,785,292	21,785,587	296	42,660	45	0.24	19
427	GC10P021805	(+)	21,805,589	22,137,000	331,412	20,003	46	0.13	20
428	RN7SKP37	(+)	21,933,143	21,933,438	296				
429	ADIPOR1P1	(+)	22,162,992	22,165,794	2,803	25,993	47	0.16	21
430	ENSG00000260205	(+)	22,218,074	22,221,168	3,095	52,281	48	0.35	
431	PIR57357	(+)	128,843,556	128,843,586	31	542	88		
432	PIR39381	(+)	128,844,127	128,844,155	29	88	87		
433	PIR33616	(+)	128,844,242	128,844,274	33	916	86		
434	PIR46908	(+)	128,845,189	128,845,219	31	1,094	85		
435	PIR60654	(+)	128,846,312	128,846,341	30	11			

436	PIR49019	(+)	128,846,351	128,846,383	33	**53**	**84**	
437	PIR31091	(+)	128,846,435	128,846,471	37	-6		
438	PIR61690	(+)	128,846,464	128,846,492	29	9		
439	PIR40735	(+)	128,846,500	128,846,533	34	**177**	**83**	
440	PIR34433	(+)	128,846,709	128,846,737	29	**478**	**82**	
441	PIR62781	(+)	128,847,214	128,847,247	34	**591**	**81**	
442	PIR50086	(+)	128,847,837	128,847,867	31	4		
443	PIR36202	(+)	128,847,870	128,847,899	30	-14		
444	PIR59776	(+)	128,847,884	128,847,912	29	**68**	**80**	
445	PIR58446	(+)	128,847,979	128,848,008	30	318	79	0.49
446	PIR39248	(+)	128,848,325	128,848,357	33	**205**	**78**	
447	PIR40712	(+)	128,848,561	128,848,590	30	4		
448	PIR48931	(+)	128,848,593	128,848,623	31	**237**	**77**	
449	PIR62313	(+)	128,848,859	128,848,889	31	**122**	**76**	
450	PIR37304	(+)	128,849,010	128,849,039	30	**46**	**75**	
451	PIR40951	(+)	128,849,084	128,849,114	31	**58**	**74**	
452	PIR36846	(+)	128,849,171	128,849,197	27	-11		
453	PIR35133	(+)	128,849,185	128,849,223	39	-18		
454	PIR33463	(+)	128,849,204	128,849,234	31	-10		
455	PIR48195	(+)	128,849,223	128,849,253	31	**77**	**73**	
456	PIR36774	(+)	128,849,329	128,849,359	31	**18**	**72**	
457	PIR49042	(+)	128,849,376	128,849,405	30	11		
458	PIR34870	(+)	128,849,415	128,849,445	31	**24**	**71**	
459	PIR40997	(+)	128,849,468	128,849,498	31	-5		
460	PIR33412	(+)	128,849,492	128,849,522	31	**1,252**	**70**	
461	PIR52743	(+)	128,850,773	128,850,803	31	**1,163**	**69**	
462	PIR56572	(+)	128,851,965	128,851,996	32	**171**	**68**	
463	PIR37790	(+)	128,852,166	128,852,197	32	**374**	**67**	
464	PIR49684	(+)	128,852,570	128,852,599	30	**148**	**66**	
465	PIR51418	(+)	128,852,746	128,852,777	32	**18**	**65**	
466	PIR52320	(+)	128,852,794	128,852,823	30	**152**	**64**	
467	PIR36760	(+)	128,852,974	128,853,004	31	**1,222**	**63**	
468	PIR57078	(+)	128,854,225	128,854,254	30	**501**	**62**	
469	PIR44450	(+)	128,854,754	128,854,784	31	-11		
470	PIR52523	(+)	128,854,772	128,854,802	31	**733**	**61**	
471	PIR58303	(+)	128,855,534	128,855,565	32	**394**	**60**	
472	PIR36399	(+)	128,855,958	128,855,988	31	**122**	**59**	
473	PIR55750	(+)	128,856,109	128,856,145	37	**65**	**58**	
474	PIR57858	(+)	128,856,209	128,856,238	30	**115**	**57**	
475	PIR37466	(+)	128,856,352	128,856,381	30	**489**	**56**	
476	PIRC14	(+)	128,856,869	128,872,603	15,735	**6,888**	**55**	
477	ACAD9	(+)	128,879,490	128,916,067	36,578	**1,914**	**54**	
478	LNC-COPG-4	(+)	128,917,980	128,922,243	4,264	**10,737**	**53**	
479	PIR62265	(+)	128,932,979	128,933,005	27	**2,025**	**52**	
480	PIR53454	(+)	128,935,029	128,935,055	27	**959**	**51**	
481	PIR40899	(+)	128,936,013	128,936,041	29	**13,353**	**50**	
482	LNC-COPG-2	(+)	128,949,393	128,955,149	5,757	-3,376		
483	PIRC15	(+)	128,951,772	128,971,491	19,720	**1,680**	**49**	
484	LNC-C3orf37-3	(+)	128,973,170	128,973,882	713	**17,815**	**48**	
485	LNC-C3orf37-2	(+)	128,991,696	128,994,026	2,331	7,226	47	0.87

486	EFCC1/LNC-H1FX-AS1	(+)	129,001,251	129,045,068	43,818	**696**	**46**	
487	LOC100130760	(+)	129,045,763	129,047,000	1,238	**6,430**	**45**	
488	LNC-C3orf37-4	(+)	129,053,429	129,053,917	489	**1,533**	**44**	
489	GP9	(+)	129,055,449	129,062,411	6,963	**1,844**	**43**	
490	LNC-C3orf37-5	(+)	129,064,254	129,065,007	754	35,135	42	0.50
491	LNC-IF122-2	(+)	129,100,141	129,112,992	12,852	**425**	**41**	
492	LNC-IF122-1	(+)	129,113,416	129,113,979	564	**9,461**	**40**	
493	ENSG00000273174	(+)	129,123,439	129,124,003	565	**18,969**	**39**	
494	GC03P129341	(+)	129,142,971	129,169,251	26,281	-536		
495	LNC-RHO-4	(+)	129,168,714	129,195,351	26,638	-11,322		
496	LOC105374101	(+)	129,184,028	129,196,302	12,275	**34,441**	**38**	
497	LOC105374102/LNC-RHO-1	(+)	129,230,742	129,236,184	5,443	13,392	37	
498	COPG1	(+)	129,249,575	129,277,773	28,199	**1,044**	**36**	
499	HMCES	(+)	129,278,816	129,306,186	27,371	**4,007**	**35**	
500	LNC-H1FOO-1	(+)	129,310,192	129,310,488	297	**4,905**	**34**	
501	H1FX-AS1	(+)	129,315,392	129,326,225	10,834	**8,306**	**33**	
502	MARK2P3	(+)	129,334,530	129,335,305	776	**10,106**	**32**	
503	LOC100421508	(+)	129,345,410	129,346,164	755	28,596	31	0.71
504	GC03P129374	(+)	129,374,759	129,405,615	30,857	34,422	30	0.53
505	IFT122	(+)	129,440,036	129,520,507	80,472	**6,199**	**29**	
506	LNC-TRH-8	(+)	129,526,705	129,527,779	1,075	**861**	**28**	
507	RHO	(+)	129,528,639	129,535,344	6,706	**7,871**	**27**	
508	H1FOO	(+)	129,543,214	129,551,467	8,254	39,883	26	0.71
509	RN7SL752P	(+)	129,591,349	129,591,635	287	**21,080**	**25**	
510	LNC-TMCC1-AS1	(+)	129,612,714	129,637,418	24,705	-15,468		
511	GC03P129621	(+)	129,621,949	130,001,803	379,855	-409		
512	RPS17P9	(+)	129,985,092	129,998,936	13,845			
513	LNC-COL6A5-3	(+)	130,001,393	130,001,797	405	**2,736**	**24**	
514	PIR61902	(+)	130,004,532	130,004,560	29	7,621	23	0.66
515	LNC-COL6A5-2	(+)	130,012,180	130,012,603	424	69,229	22	0.11
516	ALG1L2	(+)	130,081,831	130,113,227	31,397	-1,431		
517	LOC105374104	(+)	130,111,795	130,120,569	8,775	79,140	21	0.80
518	SNRPCP8	(+)	130,199,708	130,200,493	786	12,328	20	0.29
519	COL6A4P2	(+)	130,212,820	130,273,806	60,987	-37,786		
520	LNC-COL6A6-1	(+)	130,236,019	130,279,028	43,010	**14,292**	**19**	
521	LOC100507083	(+)	130,293,319	130,293,760	442	22,858	18	0.15
522	GC03P130316	(+)	130,316,617	130,328,217	11,601	**17,300**	**17**	
523	COL6A5	(+)	130,345,516	130,484,846	139,331	32,332	16	0.03
524	COL6A6	(+)	130,517,177	130,678,155	160,979	39,507	15	0.08
525	LNC-NEK11-4	(+)	130,717,661	130,718,055	395	27,702	14	0.05
526	LNC-ATP2C1-1	(+)	130,745,756	130,748,722	2,967	65,002	13	0.06
527	LOC105374107	(+)	130,813,723	130,836,545	22,823	8,296	12	0.27
528	GSTO3P	(+)	130,827,638	130,828,433	796			
529	LOC105374108	(+)	130,844,840	130,846,883	2,044	**3,601**	**11**	
530	ATP2C1	(+)	130,850,483	131,016,712	166,230	**10,139**	**10**	
531	NEK11	(+)	131,026,850	131,350,465	323,616	9,194	9	0.44
532	GC03P131359	(+)	131,359,658	131,360,747	1,090	**1,099**	**8**	
533	NUDT16P1	(+)	131,361,845	131,365,122	3,278	16,550	7	0.25
534	NUDT16	(+)	131,381,671	131,388,830	7,160	113,109	6	0.14
535	LOC105374114	(+)	131,501,938	131,689,554	187,617	113,150	5	0.05

536	BCL2L12P1		(+)	131,526,447	131,527,179	733							
537	LOC105374113		(+)	131,802,703	131,872,160	69,458	125,884	4	0.03				
538	LOC105374112		(+)	131,998,043	132,013,560	15,518	26,579	3	0.02				
539	GC03P132040/LOC105374111		(+)	132,040,138	132,083,115	42,978	92,220	2	0.07				
540	LOC729674		(+)	132,175,334	132,176,811	1,478	140,557	1	0.07				
**541**	**ACPP**	**3q22.1**	(+)	132,317,367	132,368,302	**50,936**					**LEnC**	**2**	**> 11,864**
542	LNC-CCRL1-1		(+)	132,378,762	132,379,789	1,028	10,461	1					**≤ 265, 005**
543	LOC100130550		(+)	132,386,522	132,389,074	2,553	6,734	2					
544	LNC-CCRL1-2		(+)	132,389,526	132,391,004	1,479	453	3					
545	LNC-CCRL1-3		(+)	132,394,226	132,395,385	1,160	3,223	4					
546	NIP7P2		(+)	132,401,253	132,401,796	544	5,869	5					
547	DNAJC13		(+)	132,417,526	132,539,032	121,507	15,731	6					
548	LNC-NPHP3-AS1		(+)	132,440,594	132,593,067	152,474	-98,437						
549	ACKR4		(+)	132,597,237	132,618,967	21,731	4,171	7					
550	HSPA8P19		(+)	132,645,831	132,650,394	4,564	**26,865**	**8**	**0.04**	**NC**			
551	UBA5		(+)	132,654,446	132,678,253	23,808	4,053	9					
552	LNC-TMEM108-4		(+)	132,704,550	132,722,386	17,837	**26,298**	**10**	**0.39**	**0**			
553	NPHP3-AS1		(+)	132,721,750	132,874,223	152,474	-635						
554	GC03P132823		(+)	132,823,492	132,981,675	158,184	-50,730						
555	TMEM108		(+)	133,038,288	133,397,792	359,505	**56,614**	**11**	**0.06**	**1**			
556	BFSP2		(+)	133,399,995	133,475,222	75,228	2,204	12					
557	LNC-SRPRB-3		(+)	133,479,144	133,481,207	2,064	3,923	13					
558	GC03P133482		(+)	133,482,618	133,483,861	1,244	1,412	14					
559	LNC-SRPRB-2		(+)	133,486,876	133,489,800	2,925	3,016	15					
560	LOC391578		(+)	133,490,806	133,491,560	755	1,007	16					
561	LNC-SRPRB-1		(+)	133,543,100	133,547,693	4,594	**51,541**	**17**	**0.34**	**2**			
562	ENSG00000201827		(+)	133,551,186	133,551,315	130	3,494	18					
563	GC03P133569		(+)	133,569,392	133,569,970	579	**18,078**	**19**	***0.79***	**3**			
564	CDV3		(+)	133,573,590	133,590,274	16,685	3,621	20					
565	GC03P133653		(+)	133,653,630	133,654,136	507	63,357	21					
566	TF		(+)	133,661,998	133,779,006	117,009	7,863	22					
567	TFP1		(+)	133,661,998	133,754,576	92,579							
568	RNU6-678P		(+)	133,664,926	133,665,034	109							
569	SRPRB		(+)	133,784,033	133,825,772	41,740	5,028	23					
570	LNC-CEP63-5		(+)	133,889,331	133,889,929	599	**63,560**	**24**	**0.04**	**4**			
571	LNC-CEP63-4		(+)	133,923,876	134,075,740	151,865	**33,948**	**25**	**0.62**	**5**			
572	LNC-CEP63-3		(+)	134,104,039	134,104,336	298	28,300	26					
573	HMGB3P14		(+)	134,170,390	134,171,099	710	**66,055**	**27**	**0.12**				
574	LNC-EPHB1-2		(+)	134,204,887	134,214,214	9,328	**33,789**	**28**	**0.03**				
575	LNC-CEP63-2		(+)	134,254,185	134,293,859	39,675	**39,972**	**29**	**0.16**				
576	LOC105374119/LNC-EPHB1-1		(+)	134,312,189	134,498,741	186,553	18,331	30					
577	HMGN1P9		(+)	134,385,197	134,385,494	298							
578	HMGB3P13		(+)	134,437,502	134,438,388	887							
579	CEP63		(+)	134,485,733	134,575,017	89,285	-13,007						
580	LOC101927568		(+)	134,595,264	134,673,081	77,818	20,248	31					
581	EPHB1		(+)	134,597,801	135,260,467	662,667	-75,279						
582	GC03P135324		(+)	135,324,455	135,324,730	276	**63,989**	**32**	**0.16**				
583	GC03P135458		(+)	135,458,759	135,458,884	126	**134,030**	**33**	**0.06**				
584	LNC-PPP2R3A-2		(+)	135,644,733	135,645,009	277	**185,850**	**34**	**0.03**				
585	GC03P135691		(+)	135,691,238	135,984,335	293,098	**46,230**	**35**	**0.09**				

586	PPP2R3A	(+)	135,965,673	136,147,910	182,238	-18,661		
587	TDGF1P6	(+)	136,155,548	136,157,044	1,497	7,639	36	0.14
588	PCCB/LOC105374125	(+)	136,250,306	136,338,003	87,698	93,263	37	0.14
589	LNC-TMEM22-1	(+)	136,471,472	136,474,622	3,151	133,470	38	0.26
590	LNC-TMEM22-3	(+)	136,496,640	136,497,310	671	22,019	39	0.86
591	LNC-TMEM22-2	(+)	136,537,489	136,562,164	24,676	40,180	40	0.24
592	LNC-TMEM22-4	(+)	136,581,109	136,647,566	66,458	18,946	41	0.02
593	RNY4P4	(+)	136,588,209	136,588,311	103			
594	HMGN1P10	(+)	136,609,050	136,609,602	553			
595	LNC-IL20RB-1	(+)	136,665,072	136,708,336	43,265	17,507	42	
596	LNC-IL20RB-2	(+)	136,734,713	136,735,851	1,139	26,378	43	0.07
597	LNC-IL20RB-3	(+)	136,744,772	136,745,131	360	8,922	44	0.17
598	ENSG00000261758	(+)	136,752,630	136,755,780	3,151	7,500	45	0.86
599	SLC35G2	(+)	136,818,647	136,855,892	37,246	62,868	46	0.01
600	NCK1	(+)	136,862,208	136,951,610	89,403	6,317	47	
601	GC03P136873	(+)	136,873,080	137,043,690	170,611	-78,529		
602	GC03P137161	(+)	137,161,005	137,161,378	374	117,316	48	0.01
603	GC03P137283	(+)	137,283,683	137,283,802	120	122,306	49	0.01
604	GC03P137406	(+)	137,406,760	137,407,170	411	122,959	50	0.11
605	LNC-SOX14-3	(+)	137,442,616	137,443,385	770	35,447	51	0.03
606	LNC-LINC01210	(+)	137,490,791	137,499,732	8,942	47,407	52	0.04
607	LNC-SOX14-2	(+)	137,510,815	137,515,520	4,706	11,084	53	0.97
608	NPM1P17	(+)	137,723,655	137,724,568	914	208,136	54	0.01
609	LNC-CLDN18-1 (SOX14)	(+)	137,764,168	137,771,903	7,736	39,601	55	0.17
610	LINC01210	(+)	137,771,911	137,780,878	8,968	9		
611	ENSG00000261146	(+)	137,791,973	137,796,678	4,706	11,096	56	0.04
612	HSPA8P9	(+)	137,880,210	137,882,237	2,028	83,533	57	0.001
613	CLDN18	(+)	137,998,735	138,033,655	34,921	116,499	58	0.001
614	LNC-ARMC8-2	(+)	138,067,749	138,069,264	1,516	34,095	59	0.004
615	ENSG00000281378	(+)	138,102,085	138,102,222	138	32,822	60	0.01
616	LNC-ESYT3-4	(+)	138,119,051	138,121,035	1,985	16,830	61	0.04
617	ARMC8	(+)	138,187,248	138,298,389	111,142	66,214	62	0.01
618	GC03P138286	(+)	138,286,326	138,302,569	16,244	-12,062		
619	LNC-FAIM-2	(+)	138,325,237	138,326,255	1,019	22,669	63	0.05
620	LNC-FAIM-1	(+)	138,327,448	138,349,981	22,534	1,194	64	0.13
621	MRAS	(+)	138,347,648	138,405,535	57,888	-2,332		
622	GC03P138407	(+)	138,407,342	138,450,023	42,682	1,808	65	0.01
623	ESYT3	(+)	138,434,573	138,481,686	47,114	-15,449		
624	ENSG00000280399	(+)	138,482,065	138,485,755	3,691	380	66	0.39
625	LNC-C3orf72-6	(+)	138,515,693	138,516,082	390	29,939	67	0.01
626	LNC-C3orf72-5	(+)	138,531,134	138,531,420	287	15,053	68	0.01
627	LNC-C3orf72-4	(+)	138,543,905	138,545,200	1,296	12,486	69	0.09
628	LNC-C3orf72-3	(+)	138,551,007	138,551,271	265	5,808	70	0.22
629	GC03P138606	(+)	138,606,395	138,607,413	1,019	55,125	71	0.0004
630	LNC-C3orf72-2/FAIM	(+)	138,608,338	138,633,376	25,039	926	72	0.02
631	GC03P138644/LNC-BPESC1	(+)	138,652,698	138,844,013	191,316	19,323	73	0.004
632	GAPDHP39	(+)	138,777,760	138,779,011	1,252			
633	RPL23AP40	(+)	138,796,851	138,797,240	390			
634	EEF1A1P25	(+)	138,825,063	138,826,329	1,267			
635	FOXL2NB	(+)	138,947,234	138,953,990	6,757	103,222	74	0.001

636	LNC-C3orf72-7	(+)	138,972,837	138,982,407	9,571	**18,848**	**75**	**0.002**	
637	LNC-C3orf72-8	(+)	139,000,556	139,062,865	62,310	**18,150**	**76**	**0.01**	
638	MRPS22	(+)	139,005,806	139,357,223	351,418	-57,058			
639	LOC100507291	(+)	139,389,803	139,583,319	193,517	**32,581**	**77**	**0.01**	
640	ACTG1P1	(+)	139,493,740	139,495,682	1,943	-89,578			
641	LOC102724040	(+)	139,583,466	139,600,368	16,903	**87,785**	**78**	**0.002**	
642	LOC105374130	(+)	139,600,522	139,606,896	6,375	155	79		
643	GC03P139607	(+)	139,607,683	139,609,939	2,257	**788**	**80**	**0.09**	
644	PIR54817	(+)	139,680,791	139,680,818	28	**70,853**	**81**	**0.01**	
645	PIR37486	(+)	139,685,966	139,685,995	30	**5,149**	**82**	**0.09**	
646	PIR55855	(+)	139,690,374	139,690,405	32	**4,380**	**83**	**0.04**	
647	ENSG00000251471	(+)	139,837,220	139,859,894	22,675	**146,816**	**84**	**0.0004**	
648	GC03P139858	(+)	139,858,408	139,998,281	139,874	-1,485			
649	GC03P139907	(+)	139,907,080	140,022,653	115,574	-91,200			
650	CLSTN2	(+)	139,935,185	140,557,397	622,213	-87,467			
651	GC03P140571	(+)	140,571,706	140,577,397	5,692	**14,310**	**85**	**0.08**	
652	LNC-SLC25A36-2	(+)	140,598,568	140,600,176	1,609	**21,172**	**86**	**0.04**	
653	LNC-SLC25A36-1	(+)	140,591,276	140,646,435	55,160	-8,899			
654	LNC-SPSB4-4	(+)	140,660,685	140,675,911	15,227	**14,251**	**87**	**0.01**	
655	TRIM42	(+)	140,678,024	140,701,150	23,127	**2,114**	**88**	**0.26**	

656	FBXO48	(-)	68,459,419	68,467,316	7,898	**60,899**	**32**	**0.46**	
657	LOC100420887	(-)	68,528,214	68,529,452	1,239	**221,162**	**31**	**0.31**	
658	LOC105374790	(-)	68,750,613	68,763,013	12,401	**68,176**	**30**	**0.01**	
659	LOC105374791	(-)	68,831,188	68,837,207	6,020	**28,275**	**29**	**0.41**	**14**
660	BMP10	(-)	68,865,481	68,871,517	6,037	4,025	28		
661	LOC105374792	(-)	68,875,541	68,882,029	6,489	**31,360**	**27**	**0.43**	**13**
662	LNC-BMP10-2	(-)	68,913,388	68,917,952	4,565	**27,281**	**26**	**0.72**	**12**
663	GKN2	(-)	68,945,232	68,952,970	7,739	**106,979**	**25**	**0.10**	**11**
664	LNC-BMP10-1	(-)	69,059,948	69,064,326	4,379	**70,722**	**24**	**0.05**	**10**
665	LNC-GKN2-1	(-)	69,135,047	69,145,027	9,981	4,477	23		
666	GC02M069149	(-)	69,149,503	69,150,863	1,361	**31,035**	**22**	***0.97***	**9**
667	RNA5SP96	(-)	69,181,897	69,182,003	107	69,816	21		
668	LOC100421347	(-)	69,251,818	69,252,374	557	**67,396**	**20**	**0.41**	**8**
669	GFPT1	(-)	69,319,769	69,387,254	67,486	8,497	19		
670	NFU1	(-)	69,395,750	69,437,628	41,879	20,368	18		
671	AAK1	(-)	69,457,995	69,674,349	216,355	10,781	17		
672	RPL36AP16	(-)	69,594,689	69,595,086	398				
673	LNC-AC114772.1-1	(-)	69,685,129	69,693,664	8,536	4,396	16		
674	LNC-AC114772.1-2	(-)	69,698,059	69,701,735	3,677	-1,542			
675	ENSG00000231024	(-)	69,700,192	69,713,847	13,656	**66,982**	**15**	**0.51**	**7**
676	LNC-AC114772.1-3	(-)	69,780,828	69,794,483	13,656	27,391	14		
677	LNC-AC114772.1-4	(-)	69,821,873	69,822,190	318	**22,320**	**13**	**0.12**	**6**
678	ENSG00000275381	(-)	69,844,509	69,844,933	425	**25,077**	**12**	**0.10**	**5**
679	LNC-AAK1-5	(-)	69,870,009	69,901,481	31,473	870	11		
680	RPL23AP92	(-)	69,873,565	69,873,819	255				
681	ENSG00000279229	(-)	69,902,350	69,902,709	360	24,616	10		
682	LNC-AAK1-1	(-)	69,927,324	69,940,979	13,656	8,137	9		
683	PIR33848	(-)	69,949,115	69,949,141	27	**10,949**	**8**	**0.04**	**4**

684	ASPRV1/PCBP1-AS1		(-)	69,960,089	70,103,220	143,132	**20,817**	**7**	**0.34**	**3**			
685	RN7SL470P		(-)	70,075,014	70,075,272	259							
686	LOC100133985		(-)	70,124,036	70,125,317	1,282	24,564	6					
687	C2orf42/TIA1		(-)	70,149,880	70,248,707	98,828	-58,804						
688	LNC-PCBP1-AS1		(-)	70,189,902	70,330,352	140,451	**19,699**	**5**	**0.26**	**2**			
689	LNC-C2orf42-1		(-)	70,350,050	70,369,836	19,787	32,160	4					
690	GC02M070401		(-)	70,401,995	70,403,798	1,804	**5,732**	**3**	**0.25**	**1**			
691	LNC-TIA1-1		(-)	70,409,529	70,418,085	8,557	**21,724**	**2**	***0.83***	**0**			
692	LNC-SNRPG-1		(-)	70,439,808	70,443,976	4,169	3,305	1					
**693**	**TGFA**	**2p13.3**	(-)	70,447,280	70,554,193	**106,914**					**LEnC**	**2**	**> 11,864**
694	ADD2		(-)	70,607,618	70,768,243	160,626	53,426	1	0.06				**≤ 265, 005**
695	LNC-ADD2-2		(-)	70,683,498	70,780,958	97,461	-84,744						
696	FIGLA		(-)	70,777,310	70,790,643	13,334	-3,647						
697	CLEC4F		(-)	70,808,643	70,823,764	15,122	**18,001**	**2**					
698	CD207		(-)	70,825,208	70,836,601	11,394	**1,445**	**3**					
699	LNC-TGFA-2		(-)	70,883,916	70,885,457	1,542	47,316	4	0.68				
700	LNC-FIGLA-2		(-)	70,890,630	70,900,480	9,851	**5,174**	**5**					
701	LNC-ADD2-1		(-)	70,933,545	70,995,357	61,813	33,066	6	0.74				
702	TCEB1P21		(-)	70,955,700	70,956,318	619							
703	ENSG00000236469		(-)	71,002,531	71,064,743	62,213	**7,175**	**7**					
704	LNC-CD207-1		(-)	71,063,565	71,088,005	24,441	-1,177						
705	LNC-CD207-3		(-)	71,088,413	71,091,720	3,308	**409**	**8**					
706	MCEE		(-)	71,109,676	71,130,288	20,613	17,957	9	0.45				
707	LOC101060051		(-)	71,155,919	71,156,759	841	25,632	10	*0.96*				
708	LNC-ATP6V1B1-AS1		(-)	71,168,947	71,221,879	52,933	12,189	11	0.07				
709	PAIP2B		(-)	71,182,738	71,227,114	44,377	-39,140						
710	LNC-TEX261-2		(-)	71,229,661	71,291,873	62,213	**2,548**	**12**					
711	LNC-MCEE-1		(-)	71,294,649	71,295,255	607	**2,777**	**13**					
712	LNC-PAIP2B-1		(-)	71,336,944	71,345,514	8,571	41,690	14	0.66				
713	GC02M071380/RNU6-105P		(-)	71,379,754	71,379,858	105	**34,241**	**15**					
714	LNC-PAIP2B-2		(-)	71,662,503	71,663,689	1,187	282,646	16	0.02				
715	RPS20P10		(-)	71,984,177	71,984,434	258	320,489	17	0.03				
716	LOC105374798		(-)	72,010,400	72,033,799	23,400	25,967	18	*0.78*				
717	CYP26B1		(-)	72,129,238	72,148,038	18,801	95,440	19	0.09				
718	EXOC6B		(-)	72,175,984	72,826,048	650,065	**27,947**	**20**					
719	LOC100419678		(-)	72,901,977	72,902,931	955	75,930	21	*0.92*				
720	ENSG00000278060		(-)	72,932,974	72,934,355	1,382	**30,044**	**22**					
721	SFXN5		(-)	72,942,036	73,075,619	133,584	7,682	23	0.58				
722	RAB11FIP5		(-)	73,073,382	73,156,721	83,340	-2,236						
723	LNC-SFXN5-4		(-)	73,160,103	73,161,484	1,382	**3,383**	**24**					
724	LNC-SFXN5-1		(-)	73,171,732	73,208,246	36,515	**10,249**	**25**					
725	PRADC1		(-)	73,228,006	73,233,238	5,233	**19,761**	**26**					
726	LNC-SFXN5-1		(-)	73,246,576	73,315,456	68,881	**13,339**	**27**					
727	GC02M073331		(-)	73,331,674	73,340,914	9,241	16,219	28	0.25				
728	LOC105374804/LNC-RAB11FIP5-1		(-)	73,352,367	73,385,965	33,599	**11,454**	**29**					
729	LNC-RAB11FIP5-2		(-)	73,386,598	73,388,503	1,906	**634**	**30**					
730	LNC-FBXO41-2		(-)	73,456,927	73,460,357	3,431	**68,425**	**31**					
731	ENSG00000221087		(-)	73,488,579	73,488,660	82	**28,223**	**32**					

732	GC18P061104		(+)	61,104,991	61,106,061	1,071	**46,774**	**22**					
733	LNC-SERPINB12-3		(+)	61,152,834	61,153,251	418	**660**	**21**					
734	LNC-SERPINB12-2		(+)	61,153,910	61,156,696	2,787	7,527	20	0.33				
735	LNC-SERPINB12-1		(+)	61,164,222	61,164,633	412	**16,837**	**19**					
736	GC18P061181		(+)	61,181,469	61,182,259	791	**132,555**	**18**					
737	LNC-SERPINB7-1		(+)	61,314,813	61,387,314	72,502	-53,731						
738	CDH20		(+)	61,333,582	61,555,773	222,192	**20,567**	**17**					
739	RNU6-116P		(+)	61,391,595	61,391,703	109							
740	GC18P061576		(+)	61,576,339	61,603,066	26,728	**13,526**	**16**					
741	LNC-HMSD-1		(+)	61,616,591	61,649,008	32,418	**2,998**	**15**					
742	LOC105372157/LNC-SERPINB8-1		(+)	61,652,005	61,688,260	36,256	-11,798						
743	GC18P061676		(+)	61,676,461	61,698,390	21,930	41,003	14	0.32				
744	LNC-SERPINB8-4		(+)	61,739,392	61,739,887	496	**8,290**	**13**					
745	LINC01544/LOC101927387		(+)	61,748,176	61,756,647	8,472	**14,679**	**12**					
746	LNC-SERPINB8-5		(+)	61,771,325	62,090,836	319,512	**25,350**	**11**					
747	GC18P062116		(+)	62,116,185	62,123,214	7,030	64,031	10	0.28				
748	KIAA1468		(+)	62,187,244	62,307,829	120,586	**9,229**	**9**					
749	GC18P062317		(+)	62,317,057	62,318,111	1,055	7,177	8	*0.80*				
750	TNFRSF11A		(+)	62,325,287	62,391,292	66,006	**24,353**	**7**					
751	RPL17P44		(+)	62,415,644	62,416,257	614	**2,062**	**6**					
752	ENSG00000267487		(+)	62,418,318	62,418,576	259	**23,349**	**5**					
753	ACTBP9		(+)	62,441,924	62,443,715	1,792	67,836	4	0.13				
754	GC18P062511		(+)	62,511,550	62,511,935	386	**11,073**	**3**					
**755**	**ZCCHC2 (C18orf49; KIAA1744)**		(+)	62,523,007	62,587,709	64,703	68,041	2	0.28				
756	GC18P062655		(+)	62,655,749	62,657,049	1,301	**58,391**	**1**					
**757**	**PHLPP1**	**18q21.33**	(+)	62,715,439	62,980,443	**265,005**					LEnC	2	> 11,864
758	LOC105372161		(+)	63,088,532	63,097,162	8,631	**108,090**	**1**	**0.54**	**1**			≤ 265, 005
759	LNC-CDH7-2		(+)	63,091,249	63,114,748	23,500	-5,912						
760	GC18P063133		(+)	63,133,832	63,133,849	18	19,085	2					
761	GC18P063159		(+)	63,159,601	63,264,464	104,864	**25,753**	**3**	**0.43**	**2**			
762	LNC-CDH7-1		(+)	63,273,326	63,319,289	45,964	8,863	4					
763	ENSG00000267390		(+)	63,367,328	63,381,629	14,302	**48,040**	**5**	**0.49**	**3**			
764	LOC105372163		(+)	63,397,260	63,403,695	6,436	**15,632**	**6**	**0.13**	**4**			
765	GC18P063400		(+)	63,400,845	63,404,768	3,924	-2,849						
766	LNC-SERPINB8-3		(+)	63,430,244	63,471,046	40,803	**25,477**	**7**	***0.96***	**5**			
767	SERPINB5		(+)	63,476,761	63,505,085	28,325	5,716	8					
768	ATP5G1P6		(+)	63,496,942	63,497,498	557							
769	SERPINB12		(+)	63,519,090	63,569,329	50,240	**14,006**	**9**	**0.66**	**6**			
770	SERPINB13		(+)	63,586,989	63,604,639	17,651	17,661	10					
771	SERPINB11		(+)	63,647,579	63,726,432	78,854	**42,941**	**11**	**0.59**	**7**			
772	SERPINB7		(+)	63,752,935	63,805,376	52,442	**26,504**	**12**	***0.96***	**8**			
773	SERPINB2		(+)	63,871,692	63,903,890	32,199	**66,317**	**13**	**0.13**	**9**			
774	SERPINB10		(+)	63,897,174	63,936,111	38,938	-6,715						
775	HMSD		(+)	63,949,250	63,981,774	32,525	13,140	14					
776	SERPINB8		(+)	63,969,925	64,006,821	36,897	-11,848						
777	RPL12P39		(+)	64,072,158	64,072,653	496	**65,338**	**15**	**0.05**	**10**			
778	LOC284294		(+)	64,104,091	64,423,601	319,511	**31,439**	**16**	**0.43**	**11**			
779	GC18P0646650		(+)	64,650,235	64,698,507	48,273	**226,635**	**17**	**0.09**	**12**			
780	LOC105372168		(+)	65,131,920	65,144,128	12,209	**433,414**	**18**	**0.31**	**13**			
781	LNC-CCDC102B-8		(+)	65,173,826	65,181,267	7,442	**29,699**	**19**	**0.57**	**14**			

782	LNC-CCDC102B-7	(+)	65,183,783	65,566,856	383,074	2,517	20		
783	ENSG00000265217	(+)	65,606,090	65,652,053	45,964	**39,235**	**21**	**0.02**	
784	CDH7	(+)	65,750,252	65,890,341	140,090	**98,200**	**22**	**0.48**	

785	LOC105371590	(-)	166,475,772	166,490,223	14,452	**146,469**	**44**	**0.30**	
786	LNC-TADA1-2	(-)	166,636,691	166,658,518	21,828	41,559	43		
787	LNC-TADA1-1	(-)	166,700,076	166,731,506	31,431	**125,005**	**42**	**0.05**	**13**
788	TADA1/LNC-ILDR2-1	(-)	166,856,510	166,877,317	20,808	6,363	41		
789	LOC101928459	(-)	166,883,679	166,888,855	5,177	6,052	40		
790	LOC105371593/ILDR2/LNC-ILDR2-3/LNC-GPA33-2	(-)	166,894,906	167,010,352	115,447	42,485	39		
791	GPA33	(-)	167,052,836	167,166,479	113,644	8,884	38		
792	RPS17P6	(-)	167,162,380	167,162,862	483				
793	LINC01363	(-)	167,175,362	167,195,805	20,444	24,027	37		
794	ENSG00000272205	(-)	167,219,831	167,220,512	682	**179,366**	**36**	**0.02**	**12**
795	LNC-CD247-1	(-)	167,399,877	167,487,766	87,890	-57,125			
796	CD247	(-)	167,430,640	167,518,616	87,977	**11,062**	**35**	***0.83***	**11**
797	CREG1	(-)	167,529,677	167,553,819	24,143	**42,804**	**34**	**0.35**	**10**
798	LNC-CREG1-3	(-)	167,596,622	167,599,911	3,290	**27,475**	**33**	**0.02**	**9**
799	ENSG00000241666	(-)	167,627,385	167,630,674	3,290	**47,827**	**32**	**0.11**	**8**
800	LNC-ADCY10-2	(-)	167,678,500	167,696,314	17,815	**18,801**	**31**	**0.06**	**7**
801	GC01M167716/TRP-AGG2-1	(-)	167,715,114	167,715,559	446	3,530	30		
802	GC01M167719	(-)	167,719,088	167,719,503	416	69,634	29		
803	LNC-BRP44-3	(-)	167,789,136	167,789,486	351	19,903	28		
804	ADCY10 (SAC)	(-)	167,809,388	167,915,522	106,139	-95,627			
805	ENSG00000250762	(-)	167,819,898	167,820,248	351	**96,428**	**27**	**0.05**	**6**
806	MPC2	(-)	167,916,675	167,936,805	20,131	**61,651**	**26**	**0.04**	**5**
807	LNC-AL033532.1-3	(-)	167,998,455	168,000,486	2,032	48,296	25		
808	LNC-AL033532.1-1	(-)	168,048,781	168,049,929	1,149	5,431	24		
809	GCSHP5	(-)	168,055,359	168,056,493	1,135	23,050	23		
810	GPR161	(-)	168,079,542	168,137,667	58,126	-23,986			
811	GC01M168113	(-)	168,113,680	168,398,964	285,285	1,225	22		
812	ANKRD36BP1	(-)	168,245,565	168,247,430	1,866				
813	RNU6-1310P	(-)	168,263,375	168,263,481	107				
814	LOC100505918	(-)	168,400,188	168,422,656	22,469	**10,697**	**21**	**0.19**	**4**
815	LNC-XCL2-1	(-)	168,433,352	168,464,882	31,531	-767			
816	LOC101928565	(-)	168,464,114	168,495,644	31,531	11,527	20		
817	LNC-XCL2-3	(-)	168,507,170	168,519,973	12,804	20,793	19		
818	XCL2	(-)	168,540,765	168,543,997	3,233	3,895	18		
819	LNC-XCL2-4	(-)	168,547,891	168,548,677	787	29,925	17		
820	RPL7AP21	(-)	168,578,601	168,579,463	863	58,558	16		
821	LOC105371604	(-)	168,638,020	168,666,637	28,618	28,821	15		
822	DPT	(-)	168,695,457	168,729,264	33,808	-31,361			
823	LNC-DPT-2	(-)	168,697,902	168,753,457	55,556	9,909	14		
824	ENSG00000235736	(-)	168,763,365	168,774,490	11,126	86,265	13		
825	GC01M168860	(-)	168,860,754	168,863,368	2,615	**56,997**	**12**	**0.52**	**3**
826	GC01M168920	(-)	168,920,364	168,925,546	5,183	7,603	11		
827	PIR56817	(-)	168,933,148	168,933,176	29	27,527	10		
828	GC01M168960	(-)	168,960,702	168,962,084	1,383	31,775	9		
829	GC01M168993	(-)	168,993,858	168,996,364	2,507	**105,406**	**8**	**0.28**	**2**

830	LNC-NME7-2		(-)	169,101,769	169,199,970	98,202	-67,971						
831	NME7		(-)	169,131,998	169,367,967	235,970	3,495	7					
832	LOC105371605		(-)	169,371,461	169,373,946	2,486	**20,925**	**6**	**0.03**	**1**			
833	CCDC181		(-)	169,394,870	169,460,669	65,800	**3,241**	**5**	***0.998***	**0**			
834	SLC19A2		(-)	169,463,909	169,486,079	22,171	28,088	4					
835	F5		(-)	169,514,166	169,586,588	72,423	2,262	3					
836	SELP		(-)	169,588,849	169,630,193	41,345	30,630	2					
837	LNC-SELE-1		(-)	169,660,822	169,677,597	16,776	13,069	1					
838	**SELL**		(-)	169,690,665	169,711,702	**21,038**							
839	**GC01M169692**		(-)	169,692,321	169,725,104	**32,784**							
**840w/in**	**SELE**	**01q24.2**	(-)	169,722,640	169,764,705	**42,066**					**BMEnC**	**2**	**> 11,864**
841	**LOC101928628**		(-)	169,762,914	169,764,740	**1,827**							**≤ 265, 005**
842	METTL18		(-)	169,792,529	169,794,966	2,438	27,790	1	0.47				
843	SCYL3		(-)	169,849,631	169,894,267	44,637	54,666	2	0.56				
844	GC01M169872		(-)	169,871,563	169,894,846	23,284	-22,703						
845	KIFAP3		(-)	169,921,326	170,085,208	163,883	26,481	3	0.09				
846	GC01M170068		(-)	170,068,467	170,089,826	21,360	-16,740						
847	MIR3119-1		(-)	170,151,378	170,151,462	85	61,553	4	0.46				
848	GC01M170154		(-)	170,154,697	170,179,451	24,755	**3,236**	**5**					
849	LNC-KIFAP3-4		(-)	170,180,151	170,180,598	448	**701**	**6**					
850	ISCUP1		(-)	170,211,010	170,211,457	448	30,413	7	0.11				
851	LINC01142		(-)	170,240,546	170,284,208	43,663	**29,090**	**8**					
852	HAUS4P1		(-)	170,368,918	170,370,397	1,480	84,711	9	0.38				
853	LNC-KIFAP3-2		(-)	170,429,594	170,501,750	72,157	59,198	10	0.46				
854	LOC101928650		(-)	170,460,453	170,532,647	72,195	-41,296						
855	LNC-KIFAP3-3		(-)	170,556,390	170,557,377	988	23,744	11	0.32				
856	ENSG00000225545		(-)	170,587,249	170,588,236	988	**29,873**	**12**					
857	LNC-KIFAP3-5		(-)	170,709,055	170,709,319	265	120,820	13	0.71				
858	GC01M170739		(-)	170,739,914	170,740,178	265	30,596	14	0.32				
859	GC01M170815		(-)	170,815,077	170,818,495	3,419	74,900	15	0.38				
860	GC01M170893		(-)	170,893,323	170,924,887	31,565	74,829	16	*0.78*				
861	GC01M171011		(-)	171,011,873	171,017,008	5,136	86,987	17	0.34				
862	ENSG00000281281		(-)	171,041,347	171,041,435	89	24,340	18	0.16				
863	MIR1295A		(-)	171,101,728	171,101,806	79	60,294	19	0.34				
864	LOC105371611		(-)	171,120,402	171,251,882	131,481	18,597	20	0.62				
865	ENSG00000206692		(-)	171,253,906	171,254,022	117	**2,025**	**21**					
866	HMGB1P11		(-)	171,270,954	171,271,590	637	16,933	22	0.07				
867	LNC-MYOC-5		(-)	171,315,090	171,315,408	319	43,501	23	0.53				
868	GC01M171345		(-)	171,345,951	171,346,269	319	30,544	24	0.18				
869	LNC-MYOC-4		(-)	171,410,062	171,411,265	1,204	63,794	25	*0.76*				
870	LNC-MYOC-3		(-)	171,413,838	171,414,102	265	**2,574**	**26**					
871	RNU6-290P		(-)	171,418,644	171,418,750	107	**4,543**	**27**					
872	CYCSP53		(-)	171,444,684	171,444,963	280	25,935	28	*0.77*				
873	RNU6-773P		(-)	171,519,816	171,519,913	98	74,854	29	*0.93*				
874	MYOC		(-)	171,635,417	171,652,683	17,267	115,505	30	0.03				
875	LNC-VAMP4-4		(-)	171,653,836	171,677,859	24,024	**1,154**	**31**					
876	PFN1P1		(-)	171,670,277	171,671,088	812							
877	RPL4P3		(-)	171,683,066	171,684,492	1,427	**5,208**	**32**					
878	PIR43253		(-)	171,685,043	171,685,072	30	**552**	**33**					
879	VAMP4		(-)	171,700,156	171,742,247	42,092	**15,085**	**34**					

880	LOC100422548		(-)	171,751,436	171,752,028	593	**9,190**	**35**	
881	LOC100422549		(-)	171,755,803	171,756,423	621	**3,776**	**36**	
882	RPLP1P3		(-)	171,824,602	171,825,041	440	68,180	37	0.35
883	GC01M171926		(-)	171,926,788	172,022,757	95,970	101,748	38	0.09
884	LNC-DNM3OS		(-)	172,105,671	172,113,975	8,305	82,915	39	0.51
885	DNM3OS		(-)	172,136,531	172,144,835	8,305	22,557	40	0.27
886	LNC-PIGC-5		(-)	172,245,028	172,251,967	6,940	100,194	41	0.06
887	LNC-PIGC-3		(-)	172,257,872	172,258,194	323	**5,906**	**42**	
888	LNC-PIGC-2		(-)	172,339,329	172,413,223	73,895	81,136	43	0.51
889	LOC102724541/PIGC/LOC102724528		(-)	172,369,728	172,456,471	86,744	-43,494		
890	LNC-PIGC-4		(-)	172,500,900	172,502,606	1,707	**44,430**	**44**	

891	GC16M062353		(-)	62,353,760	62,354,534	775	**42,003**	**27**	**0.06**				
892	GC16M062396		(-)	62,396,536	62,396,553	18	**24,110**	**26**	***0.86***				
893	GC16M062420		(-)	62,420,662	62,421,070	409	**223,021**	**25**	**0.01**				
894	GC16M062644		(-)	62,644,090	62,645,370	1,281	**6,981**	**24**	**0.18**				
895	GC16M062652		(-)	62,652,350	62,653,341	992	**57,954**	**23**	**0.05**				
896	GC16M062711		(-)	62,711,294	62,711,711	418	9,114	22					
897	ENSG00000222299		(-)	62,720,824	62,720,914	91	**87,309**	**21**	**0.14**				
898	ENSG00000260332		(-)	62,808,222	62,808,706	485	**17,256**	**20**	**0.14**				
899	ENSG00000223123		(-)	62,825,961	62,826,048	88	**16,079**	**19**	***0.87***				
900	LNC-CDH8-6		(-)	62,842,126	62,842,610	485	1,681	18					
901	GC16M062844		(-)	62,844,290	62,845,160	871	**83,115**	**17**	**0.02**				
902	GC17M062928		(-)	62,928,274	62,930,614	2,341	**32,865**	**16**	**0.05**				
903	GC16M962963		(-)	62,963,478	62,964,600	1,123	3,127	15					
904	GC16M062967		(-)	62,967,726	62,986,616	18,891	25,128	14					
905	LNC-CDH8-7		(-)	63,011,743	63,019,431	7,689	**37,356**	**13**	**0.55**	**6**			
906	ENSG00000261502		(-)	63,056,786	63,129,654	72,869	-38,963						
907	LNC-CDH8-3		(-)	63,090,690	63,163,558	72,869	16,818	12					
908	UBE2FP2		(-)	63,180,375	63,181,084	710	**33,431**	**11**	**0.63**	**5**			
909	LNC-CDH8-9		(-)	63,214,514	63,214,975	462	**71,940**	**10**	**0.09**	**4**			
910	LOC105371308		(-)	63,286,914	63,618,092	331,179	-269,923						
911	LNC-CDH8-10		(-)	63,348,168	63,651,950	303,783	**543,279**	**9**	**0.001**	**3**			
912	RPS15AP34		(-)	63,350,759	63,351,149	391							
913	GC16M064195		(-)	64,195,228	64,244,496	49,269	-1,887						
914	ENSG00000261028		(-)	64,242,608	64,343,909	101,302	-67,396						
915	LNC-CDH11-5		(-)	64,276,512	64,377,813	101,302	**112,167**	**8**	**0.55**	**2**			
916	GC16M064489		(-)	64,489,979	64,496,572	6,594	17,822	7					
917	GC16M064514		(-)	64,514,393	64,517,080	2,688	50,754	6					
918	GC16M064567		(-)	64,567,833	64,588,841	21,009	**45,455**	**5**	**0.07**	**1**			
919	GC16M064634		(-)	64,634,295	64,635,578	1,284	26,145	4					
920	LNC-CDH11-7		(-)	64,661,722	64,725,448	63,727	141,430	3					
921	GC16M064866		(-)	64,866,877	64,868,987	2,111	**69,855**	**2**	**0.36**	**0**			
922	GC16M064938		(-)	64,938,841	64,942,040	3,200	1,714	1					
**923**	**CDH11**	**16q21**	(-)	64,943,753	65,126,112	**182,360**					**BMEnC**	**2**	**> 11,864**
924	LNC-CDH11-12		(-)	65,128,510	65,155,960	27,451	2,399	1	0.71				**≤ 265, 005**
925	LOC101927650		(-)	65,141,756	65,176,713	34,958	-14,203						
926	LNC-CDH11-1		(-)	65,175,263	65,210,664	35,402	-1,449						
927	ENSG00000260834		(-)	65,190,973	65,234,914	43,942	-19,690						

928	LNC-CDH11-2	(-)	65,224,876	65,310,258	85,383	-10,037		
929	LINC00922/LNC-LINC00922	(-)	65,284,499	65,610,203	325,705	-25,758		
930	LNC-CDH11-4	(-)	65,635,004	65,635,601	598	**24,802**	**2**	
931	ENSG00000260695	(-)	65,861,112	65,863,784	2,673	225,512	3	0.63
932	GC16M065892	(-)	65,892,725	65,968,769	76,045	28,942	4	*0.90*
933	LNC-TK2-10	(-)	65,972,092	65,972,356	265	**3,324**	**5**	
934	LNC-TK2-5	(-)	66,041,412	66,048,221	6,810	69,057	6	0.73
935	GC16M066075	(-)	66,075,957	66,123,937	47,981	27,737	7	0.64
936	GC16M066081/LOC101927676	(-)	66,081,797	66,134,038	52,242	-42,139		
937	LNC-TK2-4	(-)	66,104,221	66,167,947	63,727	-29,816		
938	GC16M066229	(-)	66,229,497	66,238,208	8,712	**61,551**	**8**	
939	LOC105371317	(-)	66,238,828	66,267,047	28,220	**621**	**9**	
940	LNC-TK2-3	(-)	66,273,431	66,300,927	27,497	**6,385**	**10**	
941	LNC-TK2-9	(-)	66,322,027	66,322,235	209	**21,101**	**11**	
942	LOC105371318	(-)	66,363,896	66,375,804	11,909	41,662	12	0.40
943	GC16M066396	(-)	66,396,285	66,412,774	16,490	**20,482**	**13**	
944	ENSG00000260146	(-)	66,450,998	66,451,351	354	38,225	14	0.66
945	PIR55464	(-)	66,468,267	66,468,297	31	16,917	15	0.18
946	BEAN1-AS1	(-)	66,469,796	66,481,230	11,435	**1,500**	**16**	
947	ENSG00000260851	(-)	66,469,812	66,517,312	47,501	-11,417		
948	LNC-BEAN1-AS1/TK2	(-)	66,503,699	66,552,544	48,846	-13,612		
949	PIR36312	(-)	66,554,149	66,554,180	32	**1,606**	**17**	
950	ENSG00000277978	(-)	66,565,620	66,566,001	382	11,441	18	0.15
951	ENSG00000260650/LNC-CMTM4-4	(-)	66,579,983	66,582,786	2,804	**13,983**	**19**	
952	LNC-CMTM4-1	(-)	66,585,262	66,586,381	1,120	**2,477**	**20**	
953	CMTM4/GC16M066696	(-)	66,598,214	66,705,250	107,037	**11,834**	**21**	
954	DYNC1LI2	(-)	66,720,893	66,751,798	30,906	15,644	22	0.58
955	CCDC79	(-)	66,754,411	66,801,620	47,210	**2,614**	**23**	
956	NAE1	(-)	66,802,875	66,873,256	70,382	**1,256**	**24**	
957	LNC-CDH16-2	(-)	66,875,336	66,887,351	12,016	**2,081**	**25**	
958	CDH16	(-)	66,908,122	66,918,984	10,863	**20,772**	**26**	
959	RRAD	(-)	66,921,679	66,925,644	3,966	**2,696**	**27**	

960	LNC-TNFRSF11A-2	(+)	60,085,551	60,085,809	259	**39,225**	**35**	
961	ENSG00000267686	(+)	60,125,033	60,161,195	36,163	**2,373**	**34**	
962	RPS3AP49	(+)	60,149,544	60,150,407	864			
963	RNU4-17P	(+)	60,163,567	60,163,707	141	**32,848**	**33**	
964	LNC-ZCCHC2-2	(+)	60,178,783	60,179,168	386	48,419	32	0.04
965	LOC342784	(+)	60,196,554	60,198,173	1,620	**50,866**	**31**	
966	LNC-TNFRSF11A-7	(+)	60,227,586	60,243,962	16,377	**50,190**	**30**	
967	LNC-ZCCHC2-1	(+)	60,249,038	60,253,962	4,925	192,671	29	0.24
968	LOC105372155	(+)	60,294,151	60,301,145	6,995	218,690	28	0.13
969	LNC-AC015989.1-1	(+)	60,446,632	60,527,746	81,115	27,398	27	0.69
970	MRPS5P4	(+)	60,519,834	60,520,171	338	**91,603**	**26**	
971	ENSG000000253011	(+)	60,555,143	60,555,224	82	107,816	25	0.45
972	LOC105372156	(+)	60,611,773	60,664,370	52,598	**12,078**	**24**	
973	CTBP2P3	(+)	60,663,039	60,664,370	1,332			
974	GC18P060676	(+)	60,676,447	60,678,115	1,669	196,741	23	0.20
975	LNC-PHLPP1-2	(+)	60,874,855	60,875,401	547	158,839	22	0.02

976	LNC-SERPINB5-1		(+)	61,034,239	61,048,862	14,624	56,130	21	0.53				
977	GC18P061104		(+)	61,104,991	61,106,061	1,071	46,774	20					
978	LNC-SERPINB12-3		(+)	61,152,834	61,153,251	418	660	19					
979	LNC-SERPINB12-2		(+)	61,153,910	61,156,696	2,787	7,527	18					
980	LNC-SERPINB12-1		(+)	61,164,222	61,164,633	412	16,837	17					
981	GC18P061181		(+)	61,181,469	61,182,259	791	132,555	16	0.10				
982	LNC-SERPINB7-1		(+)	61,314,813	61,387,314	72,502	-53,731						
983	CDH20		(+)	61,333,582	61,555,773	222,192	20,567	15					
984	RNU6-116P		(+)	61,391,595	61,391,703	109							
985	GC18P061576		(+)	61,576,339	61,603,066	26,728	13,526	14					
986	LNC-HMSD-1		(+)	61,616,591	61,649,008	32,418	2,998	13					
987	LOC105372157/LNC-SERPINB8-1		(+)	61,652,005	61,688,260	36,256	-11,798						
988	GC18P061676		(+)	61,676,461	61,698,390	21,930	41,003	12	0.43				
989	LNC-SERPINB8-4		(+)	61,739,392	61,739,887	496	8,290	11					
990	LINC01544/LOC101927387		(+)	61,748,176	61,756,647	8,472	14,679	10	0.39				
991	LNC-SERPINB8-5		(+)	61,771,325	62,090,836	319,512	25,350	9					
992	GC18P062116		(+)	62,116,185	62,123,214	7,030	64,031	8	0.24				
993	KIAA1468		(+)	62,187,244	62,307,829	120,586	9,229	7					
994	GC18P062317		(+)	62,317,057	62,318,111	1,055	7,177	6					
995	TNFRSF11A		(+)	62,325,287	62,391,292	66,006	24,353	5					
996	RPL17P44		(+)	62,415,644	62,416,257	614	2,062	4					
997	ENSG00000267487		(+)	62,418,318	62,418,576	259	23,349	3					
998	ACTBP9		(+)	62,441,924	62,443,715	1,792	67,836	2	*0.86*				
999	GC18P062511		(+)	62,511,550	62,511,935	386	11,073	1					
**1000**	**ZCCHC2 (C18orf49; KIAA1744)**	**18q21.33**	(+)	62,523,007	62,587,709	**64,703**					**BMEnC**	**2**	**> 11,864**
1001	GC18P062655		(+)	62,655,749	62,657,049	1,301	68,041	1	0.16	1			**≤ 265, 005**
**1002**	**PHLPP1**		(+)	62,715,439	62,980,443	265,005	58,391	2					
1003	LOC105372161		(+)	63,088,532	63,097,162	8,631	108,090	3	0.22	2			
1004	LNC-CDH7-2		(+)	63,091,249	63,114,748	23,500	-5,912						
1005	GC18P063133		(+)	63,133,832	63,133,849	18	19,085	4	0.11	3			
1006	GC18P063159		(+)	63,159,601	63,264,464	104,864	25,753	5	*0.95*	4			
1007	LNC-CDH7-1		(+)	63,273,326	63,319,289	45,964	8,863	6	*0.81*	5			
1008	ENSG00000267390		(+)	63,367,328	63,381,629	14,302	48,040	7	0.19	6			
1009	LOC105372163		(+)	63,397,260	63,403,695	6,436	15,632	8					
1010	GC18P063400		(+)	63,400,845	63,404,768	3,924	-2,849						
1011	LNC-SERPINB8-3		(+)	63,430,244	63,471,046	40,803	25,477	9	*0.995*	7			
1012	SERPINB5		(+)	63,476,761	63,505,085	28,325	5,716	10					
1013	ATP5G1P6		(+)	63,496,942	63,497,498	557							
1014	SERPINB12		(+)	63,519,090	63,569,329	50,240	14,006	11	0.59	8			
1015	SERPINB13		(+)	63,586,989	63,604,639	17,651	17,661	12					
1016	SERPINB11		(+)	63,647,579	63,726,432	78,854	42,941	13	0.07	9			
1017	SERPINB7		(+)	63,752,935	63,805,376	52,442	26,504	14	0.51	10			
1018	SERPINB2		(+)	63,871,692	63,903,890	32,199	66,317	15	0.31	11			
1019	SERPINB10		(+)	63,897,174	63,936,111	38,938	-6,715						
1020	HMSD		(+)	63,949,250	63,981,774	32,525	13,140	16					
1021	SERPINB8		(+)	63,969,925	64,006,821	36,897	-11,848						
1022	RPL12P39		(+)	64,072,158	64,072,653	496	65,338	17	0.26	12			
1023	LOC284294		(+)	64,104,091	64,423,601	319,511	31,439	18	0.24	13			
1024	GC18P0646650		(+)	64,650,235	64,698,507	48,273	226,635	19	0.003	14			
1025	LOC105372168		(+)	65,131,920	65,144,128	12,209	433,414	20	0.11	15			

1026	LNC-CCDC102B-8		(+)	65,173,826	65,181,267	7,442	29,699	21					
1027	LNC-CCDC102B-7		(+)	65,183,783	65,566,856	383,074	2,517	22					
1028	ENSG00000265217		(+)	65,606,090	65,652,053	45,964	39,235	23					
1029	CDH7		(+)	65,750,252	65,890,341	140,090	**98,200**	**24**	**0.12**	**16**			
1030	GC18P065938		(+)	65,938,622	65,940,418	1,797	48,282	25					
1031	LNC-CCDC102B-4		(+)	66,094,267	66,104,155	9,889	**153,850**	**26**	**0.60**				
1032	LNC-CCDC102B-10		(+)	66,123,188	66,124,114	927	19,034	27					
1033	LNC-CCDC102B-9		(+)	66,153,302	66,170,662	17,361	29,189	28					
1034	GC18P066217		(+)	66,217,290	66,217,363	74	46,629	29					
1035	LNC-CCDC102B-1		(+)	66,382,466	66,422,236	39,771	**165,104**	**30**	**0.30**				
1036	GC18P066479		(+)	66,479,271	66,815,309	336,039	**57,036**	**31**	***0.89***				
1037	LNC-CCDC102B-2		(+)	66,817,066	66,832,387	15,322	1,758	32					
1038	RNU6-1037P		(+)	66,946,116	66,946,222	107	**113,730**	**33**	**0.29**				
1039	PIR56096		(+)	67,029,874	67,029,903	30	**83,653**	**34**	**0.03**				
1040	MIR5011		(+)	67,081,584	67,081,686	103	**51,682**	**35**	***0.76***				

1041	LINC01527		(-)	152,930,040	152,949,249	19,210	**63,234**	**29**	**0.36**				
1042	ENSG00000252920		(-)	153,012,482	153,012,614	133	**27,112**	**28**	***0.85***				
1043	SPRR2D		(-)	153,039,725	153,041,931	2,207	14,183	27					
1044	SPRR2A		(-)	153,056,113	153,057,537	1,425	**12,688**	**26**	***0.95***				
1045	SPRR2B		(-)	153,070,224	153,071,608	1,385	**21,528**	**25**	**0.02**	**15**			
1046	SPRR2E		(-)	153,093,135	153,106,184	13,050	**5,931**	**24**	***0.75***	**14**			
1047	SPRR2F/LNC-SPPR2B-1		(-)	153,112,114	153,113,969	1,856	**8,090**	**23**	**0.49**	**13**			
1048	LNC-SPRR2B-2		(-)	153,122,058	153,122,608	551	**17,511**	**22**	***0.75***	**12**			
1049	SPRR2C		(-)	153,140,118	153,141,493	1,376	8,090	21					
1050	SPRR2G		(-)	153,149,582	153,150,951	1,370	**13,401**	**20**	**0.47**	**11**			
1051	LOC105371447		(-)	153,164,351	153,203,313	38,963	**19,256**	**19**	**0.19**	**10**			
1052	PIR33201		(-)	153,222,568	153,222,597	30	**61,694**	**18**	**0.10**	**9**			
1053	LNC-PGLYRP4-1		(-)	153,284,290	153,303,044	18,755	-5,454						
1054	PGLYRP3		(-)	153,297,589	153,312,975	15,387	**4,527**	**17**	**0.10**	**8**			
1055	LNC-S100A12-3		(-)	153,317,501	153,320,454	2,954	**6,927**	**16**	**0.14**	**7**			
1056	PGLYRP4		(-)	153,327,380	153,348,841	21,462	3,457	15					
1057	LNC-S100A12-1		(-)	153,352,297	153,353,275	979	**9,406**	**14**	**0.69**	**6**			
1058	LNC-S100A12-2		(-)	153,362,680	153,363,547	868	10,160	13					
1059	S100A12		(-)	153,373,706	153,375,649	1,944	4,166	12					
1060	LAPTM4BP1		(-)	153,379,814	153,380,964	1,151	9,069	11					
1061	S100A8		(-)	153,390,032	153,422,578	32,547	4,431	10					
1062	S100A7P1		(-)	153,427,008	153,428,401	1,394	**8,595**	**9**	**0.30**	**5**			
1063	S100A7L2		(-)	153,436,995	153,440,027	3,033	**17,718**	**8**	**0.06**	**4**			
1064	S100A7		(-)	153,457,744	153,460,701	2,958	**12,243**	**7**	**0.61**	**3**			
1065	LNC-S100A6-1		(-)	153,472,943	153,473,240	298	**27,224**	**6**	**0.69**	**2**			
1066	RN7SL44P		(-)	153,500,463	153,500,764	302	6,806	5					
1067	LNC-S100A5-1		(-)	153,507,569	153,508,467	899	**26,133**	**4**	**0.02**	**1**			
1068	S100A6		(-)	153,534,599	153,536,244	1,646	904	3					
1069	S100A5		(-)	153,537,147	153,541,765	4,619	1,849	2					
1070	S100A4		(-)	153,543,613	153,550,136	6,524	10,973	1					
**1071**	**S100A2**	**01q21.3**	(-)	153,561,108	153,567,890	**6,783**					**LEnC**	**3**	**≤ 11,864**
1072	LNC-S100A14-1		(-)	153,579,928	153,585,621	5,694	12,039	1	*0.91*				
1073	LIC-S100A14-2		(-)	153,591,284	153,606,873	15,590	5,664	2	0.33				

1074	S100A16	(-)	153,606,886	153,613,245	6,360	14		
**1075w/in**	**S100A14**	(-)	153,614,255	153,616,986	2,732	1,011	3	*0.89*
1076	**LNC-S100A13-1**	(-)	153,615,538	153,618,305	2,768	-1,447		
1077	S100A13	(-)	153,618,799	153,628,239	9,441	**495**	**4**	
1078	ENSG00000271853	(-)	153,626,332	153,634,340	8,009	-1,906		
1079	ILF2	(-)	153,661,788	153,671,048	9,261	27,449	5	0.25
1080	LNC-ILF2-1	(-)	153,689,779	153,690,823	1,045	**18,732**	**6**	
1081	GEMIN2P1	(-)	153,698,257	153,718,347	20,091	**7,435**	**7**	
1082	LNC-GATAD2B-1	(-)	153,719,327	153,723,703	4,377	**981**	**8**	
1083	ENSG00000199565	(-)	153,726,252	153,726,353	102	**2,550**	**9**	
1084	ENSG00000243613	(-)	153,746,851	153,751,227	4,377	20,499	10	0.22
1085	ENSG00000233222	(-)	153,750,983	153,752,176	1,194	-243		
1086	ENSG00000279767	(-)	153,772,371	153,774,079	1,709	20,196	11	0.45
1087	ENSG00000207039	(-)	153,785,721	153,785,821	101	11,643	12	0.36
1088	LNC-DENND4B-4	(-)	153,797,944	153,798,260	317	12,124	13	*0.84*
1089	GATAD2B	(-)	153,804,725	153,922,975	118,251	**6,466**	**14**	
1090	DENND4B	(-)	153,929,501	153,946,696	17,196	6,527	15	0.53
1091	CRTC2	(-)	153,947,669	153,958,656	10,988	**974**	**16**	
1092	SLC39A1	(-)	153,959,099	153,968,184	9,086	**444**	**17**	
1093	JTB	(-)	153,974,269	153,977,975	3,707	**6,086**	**18**	
1094	RAB13	(-)	153,981,617	153,986,377	4,761	**3,643**	**19**	
1095	NUP210L	(-)	153,992,685	154,155,116	162,432	**6,309**	**20**	
1096	RNU6-179P	(-)	154,039,916	154,040,022	107			
1097	LNC-NUP210L-2	(-)	154,128,835	154,156,292	27,458	-26,280		
1098	GC01M154167 (RN7SL431P)	(-)	154,166,246	154,166,549	304	9,955	21	*0.81*
1099	LNC-C1orf189-1	(+)	154,179,706	154,180,993	1,288	**13,158**	**22**	
1100	LNC-C1orf189-2	(+)	154,184,933	154,186,992	2,060	**3,941**	**23**	
1101	TPM3	(-)	154,155,304	154,194,648	39,345	-31,687		
1102	C1orf189	(-)	154,199,085	154,206,365	7,281	**4,438**	**24**	
1103	C1orf43	(-)	154,206,706	154,220,628	13,923	**342**	**25**	
1104	LNC-C1orf43-4	(-)	154,232,738	154,238,979	6,242	**12,111**	**26**	
1105	LNC-SHE-3	(-)	154,349,442	154,351,749	2,308	110,464	27	0.128
1106	LNC-SHE-2	(-)	154,374,804	154,379,273	2,308	**23,056**	**28**	
1107	MRPS33P1	(-)	154,377,852	154,378,218	367			
1108	RPSAP17	(-)	154,378,126	154,379,169	1,044			
1109	LOC101928101	(-)	154,402,321	154,408,834	6,514	**23,049**	**29**	

1110	GC06P029355	(+)	29,355,341	29,359,233	3,893	37,322	75	
1111	GC06P029404	(+)	29,396,554	29,396,699	146	2		
1112	OR12D2/OR12D2	(+)	29,396,700	29,397,620	921	**19,546**	**74**	
1113	OR12D1/OR12D1	(+)	29,417,165	29,418,296	1,132	**6,685**	**73**	
1114	LNC-OR10C1-1	(+)	29,424,980	29,428,863	3,884	11,154	72	0.39
1115	OR10C1/OR10C1	(+)	29,440,016	29,440,951	936	9,260	71	0.24
1116	ENSG00000277881	(+)	29,450,210	29,450,558	349	7,796	70	0.95
1117	GC06P029484	(+)	29,458,353	29,458,569	217	**706**	**69**	
1118	GC06P030168	(+)	29,459,274	29,459,369	96	**652**	**68**	
1119	GC06P029460	(+)	29,460,020	29,460,184	165	**413**	**67**	
1120	GC06P029476	(+)	29,460,596	29,460,652	57	**1,119**	**66**	
1121	OR2H1	(+)	29,461,770	29,462,717	948	**587**	**65**	

1122	GC06P030669	(+)	29,463,303	29,463,466	164	**1,821**	**64**	
1123	LNC-OR2H1-7/LOC105375008	(+)	29,465,286	29,490,405	25,120	6,779	63	0.27
1124	LNC-LINC01015/LOC105375009	(+)	29,497,183	29,510,558	13,376	4,863	62	0.52
1125	PIRC24	(+)	29,515,420	29,547,168	31,749	6,022	61	0.02
1126	OR2I1P	(+)	29,553,189	29,554,160	972	19,106	60	0.57
1127	LNC-OR2H2-4	(+)	29,573,265	29,586,312	13,048	**1,634**	**59**	
1128	OR2H5P	(+)	29,573,897	29,574,833	937			
1129	TMEM183AP1	(+)	29,577,459	29,577,748	290			
1130	SNORD32B	(+)	29,582,249	29,582,332	84			
1131	RPL13AP	(+)	29,582,508	29,583,025	518			
1132	OR2H2	(+)	29,587,945	29,588,880	936	16,297	58	0.22
1133	LNC-MOG-1	(+)	29,605,176	29,609,324	4,149	28,571	57	0.34
1134	LNC-HLA-F-3	(+)	29,637,894	29,638,718	825	18,264	56	0.23
1135	MOG	(+)	29,656,981	29,672,372	15,392	18,833	55	0.17
1136	LNC-MOG-2	(+)	29,691,204	29,697,128	5,925	7,666	54	0.49
1137	LNC-HLA-F-2	(+)	29,704,793	29,718,868	14,076	**3,908**	**53**	
1138	HLA-F	(+)	29,722,775	29,740,355	17,581	7,900	52	0.16
1139	HCG9P5	(+)	29,748,254	29,748,648	395	1,724	51	*1.00*
1140	ENSG00000199290	(+)	29,750,371	29,750,472	102	40,483	50	0.09
1141	HLA-V	(+)	29,790,954	29,797,811	6,858	2,234	49	0.30
1142	HLA-P	(+)	29,800,044	29,803,079	3,036	23,898	48	0.44
1143	HLA-G	(+)	29,826,976	29,831,125	4,150	24,793	47	0.22
1144	LNC-HLA-A-7	(+)	29,855,917	29,856,414	498	**800**	**46**	
1145	LNC-HLA-A-8	(+)	29,857,213	29,857,580	368	6,852	45	0.21
1146	LNC-HLA-A-5	(+)	29,864,431	29,865,563	1,133	**1,246**	**44**	
1147	LNC-HLA-A-4	(+)	29,866,808	29,870,429	3,622	**7,324**	**43**	
1148	HLA-H	(+)	29,877,752	29,891,080	13,329	**3,027**	**42**	
1149	LNC-HLA-A-3	(+)	29,894,106	29,897,502	3,397	-1,058		
1150	HLA-T	(+)	29,896,443	29,898,947	2,505	**85**	**41**	
1151	GC06P030675	(+)	29,899,031	29,902,652	3,622	3,892	40	0.66
1152	DDX39BP1	(+)	29,906,543	29,907,449	907	16,925	39	0.31
1153	LNC-HLA-A-2	(+)	29,924,373	29,926,347	1,975	**113**	**38**	
1154	HLA-K	(+)	29,926,459	29,929,839	3,381	**358**	**37**	
1155	PIR44105	(+)	29,930,196	29,930,226	31	**3,539**	**36**	
1156	HLA-U	(+)	29,933,764	29,934,880	1,117	**6,381**	**35**	
1157	HLA-A/LNC-HCG9	(+)	29,941,260	29,946,187	4,928	9,648	34	0.16
1158	HLA-W	(+)	29,955,834	29,959,058	3,225	**1,929**	**33**	
1159	LNC-HLA-A-6	(+)	29,960,986	29,961,382	397	**12,992**	**32**	
1160	LNC-ZNRD1-4/HCG9	(+)	29,974,373	29,978,410	4,038	14,800	31	0.09
1161	DDX39BP2	(+)	29,993,209	29,994,133	925	**5,358**	**30**	
1162	LNC-ZNRD1-3	(+)	29,999,490	30,000,780	1,291	5,192	29	0.12
1163	HLA-J	(+)	30,005,971	30,009,956	3,986	**1,406**	**28**	
1164	LNC-ZNRD1-2	(+)	30,011,361	30,011,958	598	**14,719**	**27**	
1165	LNC-ZNRD1-1	(+)	30,026,676	30,032,686	6,011	-972		
1166	ETF1P1	(+)	30,031,713	30,033,877	2,165	**2,040**	**26**	
1167	ENSG00000278104	(+)	30,035,916	30,035,983	68	**3,440**	**25**	
1168	LNC-PPP1R11-1	(+)	30,039,422	30,041,709	2,288	16,407	24	0.67
1169	ENSG00000275856	(+)	30,058,115	30,058,190	76	**710**	**23**	
1170	ZNRD1	(+)	30,058,899	30,064,909	6,011	**1,801**	**22**	
1171	PPP1R11	(+)	30,066,709	30,070,333	3,625	-2,309		

1172	LNC-TRIM31-AS1		(+)	30,068,023	30,082,501	14,479	21,401	21	*0.82*				
1173	LNC-TRIM15-1		(+)	30,103,901	30,104,975	1,075	**266**	**20**					
1174	TRIM31-AS1		(+)	30,105,240	30,114,724	9,485	19,787	19	0.73				
1175	TRIM40		(+)	30,134,510	30,148,773	14,264	14,434	18	0.07				
1176	TRIM15		(+)	30,163,206	30,172,696	9,491	54,644	17	0.12				
1177	LNC-TRIM39-2		(+)	30,227,339	30,234,728	7,390	3,574	16	0.42				
1178	TRIM26BP		(+)	30,238,301	30,242,279	3,979	17,036	15	0.39				
1179	LNC-TRIM39-1/HLA-L		(+)	30,259,314	30,293,014	33,701	19,906	14	0.63				
1180	LNC-RPP21-1		(+)	30,312,919	30,314,661	1,743	**11,819**	**13**					
1181	TRIM39		(+)	30,326,479	30,343,729	17,251	-14,821						
1182	TRIM39-RPP21		(+)	30,328,907	30,346,858	17,952	-1,728						
1183	RPP21		(+)	30,345,129	30,346,884	1,756	**4,191**	**12**					
1184	HLA-N		(+)	30,351,074	30,352,038	965	62,676	11	0.15				
1185	MICC		(+)	30,414,713	30,419,766	5,054	33,952	10	0.03				
1186	LNC-HLA-E-5		(+)	30,453,717	30,454,046	330	12,180	9	0.46				
1187	TMPOP1		(+)	30,466,225	30,468,869	2,645	<u>5</u>						
1188	SUCLA2P1		(+)	30,468,873	30,470,922	2,050	**3,988**	**8**					
1189	GC06P030613		(+)	30,474,909	30,474,938	30	**48**	**7**					
1190	GC06P030612		(+)	30,474,985	30,475,017	33	9,027	6	0.42				
1191	LNC-HLA-E-4		(+)	30,484,043	30,488,506	4,464	-2,620						
1192	RANBP1		(+)	30,485,885	30,486,976	1,092	**2,431**	**5**					
1193	HLA-E		(+)	30,489,406	30,494,205	4,800	22,062	4	0.01				
1194	ENSG00000235781		(+)	30,516,266	30,519,217	2,952	**5,875**	**3**					
1195	LNC-PRR3-1		(+)	30,525,091	30,531,500	6,410	14,405	2	0.05				
1196	LNC-PRR3-2		(+)	30,545,904	30,546,271	368	10,439	1	0.64				
**1197**	**PRR3**	**6p21.33**	(+)	30,556,709	30,564,696	**7,988**					**BMEnC**	**3**	**≤ 11,864**
1198	ABCF1/LNC-ATAT1-1		(+)	30,571,376	30,617,690	46,315	6,681	1					
1199	LNC-ATAT1-4/MRPS18B		(+)	30,614,963	30,626,397	11,435	-2,726						
1200	PIR40859		(+)	30,627,124	30,627,153	30	728	2					
1201	GC06P030636		(+)	30,633,632	30,633,898	267	**6,480**	**3**	***0.91***	**0**			
1202	PTMAP1		(+)	30,633,450	30,635,237	1,788	-447						
1203	ATAT1		(+)	30,626,836	30,646,823	19,988	-8,400						
1204	C6orf136		(+)	30,647,039	30,653,210	6,172	217	4					
1205	LNC-MDC1-AS1		(+)	30,670,844	30,680,961	10,118	**17,635**	**5**	**0.14**	**1**			
1206	ENSG00000277346		(+)	30,684,796	30,684,892	97	3,836	6					
1207	LNC-C6orf136-2		(+)	30,688,157	30,693,193	5,037	**3,266**	**7**	**0.01**	**2**			
1208	MDC1-AS1		(+)	30,703,067	30,713,184	10,118	**9,875**	**8**	**0.40**	**3**			
1209	LNC-TUBB-15		(+)	30,718,815	30,719,062	248	5,632	9					
1210	TUBB		(+)	30,720,201	30,725,426	5,226	1,140	10					
1211	LNC-HCG20		(+)	30,734,602	30,760,027	25,426	9,177	11					
1212	EGID-105375013/HCG20/LNC-DDR1-4		(+)	30,744,491	30,806,451	61,961	-15,535						
1213	LNC-DDR1-3		(+)	30,818,686	30,819,566	881	**12,236**	**12**	**0.34**	**4**			
1214	LNC-DDR1-2		(+)	30,832,027	30,832,329	303	**12,462**	**13**	***0.95***	**5**			
1215	LNC-DDR1-1		(+)	30,842,581	30,868,006	25,426	10,253	14					
1216	RN7SKP186		(+)	30,864,250	30,864,552	303							
1217	LNC-GTF2H4-1		(+)	30,874,655	30,874,913	259	6,650	15					
1218	DDR1		(+)	30,876,421	30,900,156	23,736	1,509	16					
1219	GC06P030906		(+)	30,906,878	30,907,136	259	6,723	17					
1220	GTF2H4/VARS2		(+)	30,908,184	30,926,459	18,276	1,049	18					
1221	DPCR1		(+)	30,940,972	30,954,221	13,250	14,514	19					

1222	LOC100420530	(+)	30,964,215	30,966,160	1,946	**9,995**	**20**	**0.03**	**6**
1223	MUC21	(+)	30,983,708	30,989,903	6,196	17,549	21		
1224	LNC-MUC21-1	(+)	30,997,689	31,002,485	4,797	**7,787**	**22**	**0.23**	**7**
1225	MUC22/GC06P031035	(+)	31,005,952	31,042,436	36,485	**3,468**	**23**	**0.20**	**8**
1226	HCG22	(+)	31,053,450	31,059,890	6,441	11,015	24		
1227	RNU6-1133P	(+)	31,083,010	31,083,109	100	**23,121**	**25**	**0.15**	**9**
1228	GC06P021085	(+)	31,085,479	31,090,272	4,794	**2,371**	**26**	***0.86***	**10**
1229	LNC-PSORS1C1-2	(+)	31,105,668	31,110,464	4,797	**15,397**	**27**	***0.96***	**11**
1230	PSORS1C1	(+)	31,114,750	31,140,092	25,343	**4,287**	**28**	**0.33**	**12**
1231	POLR2LP1	(+)	31,140,727	31,140,913	187	636	29		
1232	TCF19/LNC-HCG27	(+)	31,158,526	31,196,425	37,900	**17,614**	**30**	**0.30**	**13**
1233	HCG27	(+)	31,197,760	31,203,968	6,209	1,336	31		
1234	ENSG00000255726	(+)	31,222,913	31,223,093	181	**18,946**	**32**	**0.69**	**14**
1235	USP8P1	(+)	31,275,572	31,278,754	3,183	**52,480**	**33**	**0.04**	**15**
1236	RPL3P2	(+)	31,280,291	31,281,571	1,281	1,538	34		
1237	LNC-MICA-9	(+)	31,323,945	31,325,414	1,470	**42,375**	**35**	**0.15**	**16**
1238	LNC-MICA-8	(+)	31,345,196	31,345,796	601	**19,783**	**36**	**0.18**	**17**
1239	LNC-MICA-7	(+)	31,348,188	31,348,616	429	**2,393**	**37**	**0.15**	**18**
1240	LNC-MICA-6	(+)	31,350,312	31,351,479	1,168	**1,697**	**38**	**0.07**	**19**
1241	ENSG00000271581	(+)	31,356,647	31,357,637	991	5,169	39		
1242	LNC-MICA-5	(+)	31,360,224	31,360,751	528	2,588	40		
1243	LNC-HCP5/LNC-HCG26	(+)	31,368,479	31,445,283	76,805	**7,729**	**41**	**0.01**	**20**
1244	RNU6-283P	(+)	31,370,134	31,370,240	107				
1245	FGFR3P1	(+)	31,377,419	31,378,028	610				
1246	ZDHHC20P2	(+)	31,380,408	31,380,839	432				
1247	MICA/HCP5	(+)	31,399,784	31,477,506	77,723	-45,498			
1248	LNC-MICB-4	(+)	31,483,756	31,483,988	233	**6,251**	**42**	**0.48**	**21**
1249	MICB	(+)	31,494,277	31,511,124	16,848	**10,290**	**43**	**0.71**	**22**
1250	LNC-NFKBIL1-8	(+)	31,513,762	31,514,567	806	**2,639**	**44**	**0.47**	**23**
1251	ENSG00000256851	(+)	31,515,979	31,516,211	233	1,413	45		
1252	LNC-LTA-1	(+)	31,517,423	31,522,729	5,307	**1,213**	**46**	**0.66**	**24**
1253	RPL15P4/LNC-NFKBIL1-9	(+)	31,528,076	31,529,126	1,051	5,348	47		
1254	MCCD1	(+)	31,528,717	31,530,232	1,516	-408			
1255	LNC-TNF-2	(+)	31,540,751	31,541,629	879	10,520	48		
1256	DDX39B-AS1	(+)	31,542,304	31,543,138	835	676	49		
1257	NFKBIL1	(+)	31,546,851	31,558,829	11,979	3,714	50		
1258	LTA	(+)	31,560,550	31,574,324	13,775	1,722	51		
1259	TNF	(+)	31,575,567	31,578,336	2,770	1,244	52		
1260	LST1	(+)	31,586,124	31,588,909	2,786	**7,789**	**53**	**0.50**	**25**
1261	LNC-AIF1-1	(+)	31,592,679	31,593,274	596	3,771	54		
1262	LNC-AIF1-2	(+)	31,596,417	31,597,545	1,129	3,144	55		
1263	LNC-PRRC2A-3	(+)	31,601,731	31,605,548	3,818	4,187	56		
1264	AIF1	(+)	31,615,184	31,617,021	1,838	9,637	57		
1265	PRRC2A	(+)	31,620,673	31,637,777	17,105	3,653	58		
1266	LNC-LY6G5B-1	(+)	31,640,158	31,641,553	1,396	**2,382**	**59**	**0.69**	**26**
1267	APOM	(+)	31,652,410	31,658,210	5,801	10,858	60		
1268	C6orf47-AS1	(+)	31,658,329	31,660,772	2,444	120	61		
1269	ENSG00000201207	(+)	31,663,288	31,663,401	114	2,517	62		
1270	CSNK2B	(+)	31,665,236	31,670,343	5,108	1,836	63		
1271	LY6G5B	(+)	31,670,167	31,673,776	3,610	-175			

1272	LOC105375019	(+)	31,682,895	31,687,124	4,230	**9,120**	**64**	**0.20**	**27**
1273	LOC105375018	(+)	31,689,711	31,691,316	1,606	**2,588**	**65**	**0.23**	**28**
1274	LY6G6F/ENSG00000250641	(+)	31,706,885	31,717,918	11,034	**15,570**	**66**	**0.07**	**29**
1275	LNC-MSH5-1	(+)	31,707,725	31,732,076	24,352	-10,192			
1276	MSH5-SAPCD1	(+)	31,739,948	31,764,851	24,904	**7,873**	**67**	**0.05**	**30**
1277	LNC-HSPA1B-1	(+)	31,789,991	31,795,104	5,114	**25,141**	**68**	**0.03**	**31**
1278	HSPA1A	(+)	31,815,464	31,817,946	2,483	**20,361**	**69**	**0.03**	**32**
1279	LNC-C6orf48-1	(+)	31,825,327	31,825,541	215	7,382	70		
1280	HSPA1B	(+)	31,827,735	31,830,255	2,521	2,195	71		
1281	C6orf48	(+)	31,834,608	31,839,766	5,159	4,354	72		
1282	LNC-EHMT2-AS1	(+)	31,851,538	31,851,981	444	**11,773**	**73**	**0.57**	
1283	EHMT2-AS1	(+)	31,883,761	31,884,204	444	**31,781**	**74**	**0.62**	
1284	C2/LNC-XXbac-BPG116M5.15.4-1	(+)	31,897,785	31,947,499	49,715	13,582			
1285	CFB/LNC-C4A-1/LNC-Xxbac-BPG116M5.15.4-2	(+)	31,945,650	31,952,175	6,526	-1,848			

1286	PIR56014	(+)	91,579,394	91,579,421	28	**28,632**	**35**	
1287	GC14P091608	(+)	91,608,052	91,609,126	1,075	**5,247**	**34**	
1288	LNC-CTD-2547L24.3.1-4	(+)	91,614,372	91,614,663	292	67,539	33	0.29
1289	LNC-CTD-2547L24.3.1-3	(+)	91,682,201	91,691,116	8,916	17,988	32	0.08
1290	LNC-CTD-2547L24.3.1-7	(+)	91,709,103	91,718,555	9,453	**6,089**	**31**	
1291	LNC-CTD-2547L24.3.1-1	(+)	91,724,643	91,725,347	705	159,264	30	0.01
1292	LNC-CTD-2547L24.3.1-2	(+)	91,884,610	91,887,520	2,911	4,117	29	0.74
1293	GC14P091891	(+)	91,891,636	92,038,689	147,054	**1,063**	**28**	
1294	PIR36842	(+)	92,039,751	92,039,780	30	30,451	27	*0.98*
1295	ENSG00000279963	(+)	92,070,230	92,071,042	813	50,896	26	0.10
1296	CPSF2/PIR62373	(+)	92,121,937	92,172,392	50,456	**3,064**	**25**	
1297	LNC-CPSF2-4	(+)	92,175,455	92,176,961	1,507	75,480	24	0.20
1298	GC14P092252	(+)	92,252,440	92,269,920	17,481	**34,495**	**23**	
1299	RNU6-366P	(+)	92,265,702	92,265,807	106			
1300	LOC105370627	(+)	92,304,414	92,319,650	15,237	2,932	22	0.11
1301	SLC24A4	(+)	92,322,581	92,501,483	178,903	-147,534		
1302	GC14P092353	(+)	92,353,948	92,914,905	560,958	**7,685**	**21**	
1303	CHGA	(+)	92,922,589	92,935,293	12,705	29,686	20	0.64
1304	LNC-SLC24A4-1	(+)	92,964,978	92,967,823	2,846	74,767	19	0.52
1305	LNC-SLC24A4-3	(+)	93,042,589	93,052,925	10,337	14,528	18	0.61
1306	ITPK1-AS1	(+)	93,067,452	93,072,152	4,701	**9,667**	**17**	
1307	LNC-SLC24A4-4	(+)	93,081,818	93,111,535	29,718	**5,729**	**16**	
1308	LNC-GOLGA5-2	(+)	93,117,263	93,118,460	1,198	**677**	**15**	
1309	GC14P093119	(+)	93,119,136	93,120,005	870	**17,951**	**14**	
1310	CYB5AP3	(+)	93,137,955	93,138,777	823	27,758	13	0.19
1311	LNC-RIN3-1	(+)	93,166,534	93,166,901	368	18,051	12	0.68
1312	TMEM251/ENSG00000259066	(+)	93,184,951	93,218,586	33,636	-11,529		
1313	UBR7	(+)	93,207,056	93,229,215	22,160	**14,746**	**11**	
1314	RPL36AP4	(+)	93,243,960	93,244,423	464	**51,023**	**10**	
1315	LOC105370630	(+)	93,295,445	93,296,187	743	37,033	9	0.28
1316	UNC79	(+)	93,333,219	93,707,878	374,660	172,549	8	0.27
1317	LOC105379631	(+)	93,880,426	93,885,404	4,979	10,359	7	0.60
1318	ENSG00000279593	(+)	93,895,762	93,897,759	1,998	21,136	6	0.43
1319	FAM181A	(+)	93,918,894	93,929,608	10,715	**9,948**	**5**	

1320	ENSG00000258987/LOC105370632		(+)	93,939,555	93,947,342	7,788	49,929	4	0.40				
1321	LINC00521		(+)	93,997,270	94,011,695	14,426	4,401	3					
1322	OTUB2		(+)	94,016,095	94,048,930	32,836	32,353	2					
1323	IFI27L1		(+)	94,081,282	94,103,846	22,565	991	1					
**1324**	**IFI27**	**14q32.12**	(+)	94,104,836	94,116,699	**11,864**					**BMEnC**	**3**	**≤ 11,864**
1325	PPP4R4		(+)	94,146,128	94,279,755	133,628	29,430	1	0.03	0			
1326	LNC-FAM181A-1		(+)	94,362,108	94,364,105	1,998	82,354	2	0.39	1			
1327	LNC-RP11-131H24.4.1-1		(+)	94,405,901	94,410,475	4,575	41,797	3	0.11	2			
1328	ENSG00000256357		(+)	94,430,633	94,464,730	34,098	20,159	4					
1329	LNC-LINC00521		(+)	94,463,616	94,478,041	14,426	-1,113						
1330	LNC-IFI27L1-1		(+)	94,547,639	94,570,192	22,554	69,599	5	0.14	3			
1331	SERPINA4/SERPINA5		(+)	94,561,091	94,593,120	32,030	-9,100						
1332	ENSG00000273259		(+)	94,592,058	94,624,646	32,589	-1,061						
1333	ADIPOR1P2		(+)	94,633,672	94,634,525	854	9,027	6					
1334	SERPINA13P		(+)	94,640,725	94,646,994	6,270	6,201	7					
1335	LNC-IFI27-2		(+)	94,693,727	94,716,023	22,297	46,734	8					
1336	RPSAP4		(+)	94,726,212	94,727,245	1,034	10,190	9					
1337	LNC-PPP4R4-1		(+)	94,856,404	94,871,146	14,743	129,160	10	0.40	4			
1338	LNC-SERPINA4-1		(+)	94,896,970	94,931,067	34,098	25,825	11	0.57	5			
1339	LNC-SERPINA4-3		(+)	94,943,354	94,949,280	5,927	12,288	12					
1340	PIR39242		(+)	94,954,648	94,954,675	28	5,369	13					
1341	LNC-SERPINA4-2		(+)	95,019,409	95,023,082	3,674	64,735	14	0.28	6			
1342	ENSG00000258866		(+)	95,048,220	95,050,953	2,734	25,139	15	0.03	7			
1343	LNC-SERPINA5-1		(+)	95,078,721	95,089,100	10,380	27,769	16	0.21	8			
1344	LNC-SERPINA3-2		(+)	95,100,042	95,100,861	820	10,943	17	*0.88*	9			
1345	LNC-SERPINA3-3		(+)	95,109,670	95,118,395	8,726	8,810	18					
1346	DICER1-AS1		(+)	95,157,645	95,179,933	22,289	39,251	19					
1347	ENSG00000258615		(+)	95,199,039	95,199,345	307	19,107	20					
1348	LOC101929080		(+)	95,329,584	95,335,504	5,921	130,240	21	0.06	10			
1349	ENSG00000276090		(+)	95,335,840	95,335,943	104	337	22					
1350	LNC-SERPINA3-1/LOC101929107		(+)	95,514,557	95,518,599	4,043	178,615	23	0.19	11			
1351	GLRX5		(+)	95,533,503	95,544,724	11,222	14,905	24					
1352	LOC100506999		(+)	95,573,554	95,582,449	8,896	28,831	25	0.11	12			
1353	LOC105370643		(+)	95,587,666	95,590,990	3,325	5,218	26					
1354	ENSG00000258927		(+)	95,620,914	95,643,285	22,372	29,925	27					
1355	ENSG00000270038		(+)	95,644,508	95,645,232	725	1,224	28	*0.87*	13			
1356	LNC-RP11-1070N10.3.1-2		(+)	95,648,278	95,654,010	5,733	3,047	29					
1357	TCL6		(+)	95,650,498	95,679,833	29,336	-3,511						
1358	ENSG00000259084/TCL1B		(+)	95,663,256	95,692,643	29,388	-16,576						
1359	PIR31480		(+)	95,693,525	95,693,551	27	883	30					
1360	ENSG00000257275		(+)	95,711,747	95,757,656	45,910	18,197	31	0.33	14			
1361	LNC-RP11-1070N10.3.1-5		(+)	95,759,022	95,767,223	8,202	1,367	32					
1362	LNC-RP11-1070N10.3.1-1		(+)	95,786,503	95,801,841	15,339	19,281	33					
1363	TUNAR		(+)	95,876,392	95,925,571	49,180	74,552	34	0.07	15			
1364	GC14P095978		(+)	95,978,181	96,020,139	41,959	52,611	35	0.54				
1365	TCHHL1		(-)	152,084,144	152,089,064	4,921	17,254	52	0.07				
1366	TCHH		(-)	152,106,317	152,115,454	9,138	7,707	51					
1367	PUDPP2		(-)	152,123,160	152,196,699	73,540	15,378	50	0.57	32			

1368	HRNR	(-)	152,212,076	152,224,196	12,121	11,033	49		
1369	GC01M152234/FLG	(-)	152,235,228	152,325,257	90,030	**23,479**	**48**	***0.97***	**31**
1370	FLG2	(-)	152,348,735	152,360,010	11,276	**49,234**	**47**	**0.02**	**30**
1371	CRNN	(-)	152,409,243	152,414,274	5,032	**97,518**	**46**	**0.06**	**29**
1372	ENSG0000029850	(-)	152,511,791	152,512,783	993	**38,723**	**45**	**0.02**	**28**
1373	LNC-LCE3D-1	(-)	152,551,505	152,552,411	907	13,244	44		
1374	LCE3E	(-)	152,565,654	152,566,772	1,119	**12,610**	**43**	**0.10**	**27**
1375	LCE3D	(-)	152,579,381	152,580,504	1,124	**36,366**	**42**	**0.01**	**26**
1376	LNC-LCE3A-1	(-)	152,616,869	152,617,193	325	**5,642**	**41**	**0.02**	**25**
1377	LCE3A	(-)	152,622,834	152,623,103	270	**21,291**	**40**	**0.05**	**24**
1378	LCEP4	(-)	152,644,393	152,644,717	325	**160,119**	**39**	**0.01**	**23**
1379	LCEIC	(-)	152,804,835	152,806,631	1,797	**31,535**	**38**	**0.14**	**22**
1380	LNC-LCEIC-1	(-)	152,838,165	152,838,510	346	1,258	37		
1381	LNC-LCEIC-2	(-)	152,839,767	152,840,039	273	**21,471**	**36**	**0.10**	**21**
1382	LOC105371444	(-)	152,861,509	152,868,176	6,668	**1,155**	**35**	**0.14**	**20**
1383	LOC105371445	(-)	152,869,330	152,872,495	3,166	30,022	34		
1384	LNC-LINC01527	(-)	152,902,516	152,921,686	19,171	8,355	33		
1385	LINC01527	(-)	152,930,040	152,949,249	19,210	**63,234**	**32**	**0.25**	**19**
1386	ENSG00000252920	(-)	153,012,482	153,012,614	133	**27,112**	**31**	**0.31**	**18**
1387	SPRR2D	(-)	153,039,725	153,041,931	2,207	14,183	30		
1388	SPRR2A	(-)	153,056,113	153,057,537	1,425	12,688	29		
1389	SPRR2B	(-)	153,070,224	153,071,608	1,385	21,528	28		
1390	SPRR2E	(-)	153,093,135	153,106,184	13,050	**5,931**	**27**	***0.92***	**17**
1391	SPRR2F/LNC-SPPR2B-1	(-)	153,112,114	153,113,969	1,856	8,090	26		
1392	LNC-SPRR2B-2	(-)	153,122,058	153,122,608	551	17,511	25		
1393	SPRR2C	(-)	153,140,118	153,141,493	1,376	8,090	24		
1394	SPRR2G	(-)	153,149,582	153,150,951	1,370	**13,401**	**23**	***0.90***	**16**
1395	LOC105371447	(-)	153,164,351	153,203,313	38,963	**19,256**	**22**	**0.02**	**15**
1396	PIR33201	(-)	153,222,568	153,222,597	30	**61,694**	**21**	**0.07**	**14**
1397	LNC-PGLYRP4-1	(-)	153,284,290	153,303,044	18,755	-5,454			
1398	PGLYRP3	(-)	153,297,589	153,312,975	15,387	**4,527**	**20**	***0.87***	**13**
1399	LNC-S100A12-3	(-)	153,317,501	153,320,454	2,954	6,927	19		
1400	PGLYRP4	(-)	153,327,380	153,348,841	21,462	3,457	18		
1401	LNC-S100A12-1	(-)	153,352,297	153,353,275	979	**9,406**	**17**	**0.67**	**12**
1402	LNC-S100A12-2	(-)	153,362,680	153,363,547	868	**10,160**	**16**	**0.36**	**11**
1403	S100A12	(-)	153,373,706	153,375,649	1,944	4,166	15		
1404	LAPTM4BP1	(-)	153,379,814	153,380,964	1,151	**9,069**	**14**	**0.05**	**10**
1405	S100A8	(-)	153,390,032	153,422,578	32,547	**4,431**	**13**	**0.22**	**9**
1406	S100A7P1	(-)	153,427,008	153,428,401	1,394	**8,595**	**12**	**0.76**	**8**
1407	S100A7L2	(-)	153,436,995	153,440,027	3,033	**17,718**	**11**	**0.36**	**7**
1408	S100A7	(-)	153,457,744	153,460,701	2,958	**12,243**	**10**	***0.99***	**6**
1409	LNC-S100A6-1	(-)	153,472,943	153,473,240	298	**27,224**	**9**	**0.43**	**5**
1410	RN7SL44P	(-)	153,500,463	153,500,764	302	6,806	8		
1411	LNC-S100A5-1	(-)	153,507,569	153,508,467	899	**26,133**	**7**	**0.78**	**4**
1412	S100A6	(-)	153,534,599	153,536,244	1,646	904	6		
1413	S100A5	(-)	153,537,147	153,541,765	4,619	**1,849**	**5**	**0.53**	**3**
1414	S100A4	(-)	153,543,613	153,550,136	6,524	**10,973**	**4**	**0.68**	**2**
**1415**	**S100A2**	(-)	153,561,108	153,567,890	6,783	12,039	3		
1416	LNC-S100A14-1	(-)	153,579,928	153,585,621	5,694	5,664	2		
1417	LIC-S100A14-2	(-)	153,591,284	153,606,873	15,590	14			

1418	S100A16		(-)	153,606,886	153,613,245	6,360	**1,011**	**1**	**0.49**	**1**			
**1419w/in**	**S100A14**	**01q21.3**	(-)	153,614,255	153,616,986	**2,732**							
1420	**LNC-S100A13-1**		(-)	153,615,538	153,618,305	**2,768**					**BMEnC**	**3**	**≤ 11,864**
1421	S100A13		(-)	153,618,799	153,628,239	9,441	**495**	**1**					
1422	ENSG00000271853		(-)	153,626,332	153,634,340	8,009	-1,906						
1423	ILF2		(-)	153,661,788	153,671,048	9,261	27,449	2	0.21				
1424	MIR8083/LNC-ILF2-1		(-)	153,689,705	153,690,823	1,119	18,658	3	0.65				
1425	GEMIN2P1		(-)	153,698,257	153,718,347	20,091	**7,435**	**4**					
1426	LNC-GATAD2B-1		(-)	153,719,327	153,723,703	4,377	**981**	**5**					
1427	ENSG00000199565		(-)	153,726,252	153,726,353	102	2,550	6	0.35				
1428	ENSG00000243613		(-)	153,746,851	153,751,227	4,377	**20,499**	**7**					
1429	ENSG00000233222		(-)	153,750,983	153,752,176	1,194	-243						
1430	ENSG00000279767		(-)	153,772,371	153,774,079	1,709	20,196	8	0.34				
1431	ENSG00000207039		(-)	153,785,721	153,785,821	101	**11,643**	**9**					
1432	LNC-DENND4B-4		(-)	153,797,944	153,798,260	317	**12,124**	**10**					
1433	GATAD2B		(-)	153,804,725	153,922,975	118,251	**6,466**	**11**					
1434	DENND4B		(-)	153,929,501	153,946,696	17,196	**6,527**	**12**					
1435	CRTC2		(-)	153,947,669	153,958,656	10,988	**974**	**13**					
1436	SLC39A1		(-)	153,959,099	153,968,184	9,086	**444**	**14**					
1437	JTB		(-)	153,974,269	153,977,975	3,707	6,086	15	0.68				
1438	RAB13		(-)	153,981,617	153,986,377	4,761	**3,643**	**16**					
1439	NUP210L		(-)	153,992,685	154,155,116	162,432	**6,309**	**17**					
1440	RNU6-179P		(-)	154,039,916	154,040,022	107							
1441	LNC-NUP210L-2		(-)	154,128,835	154,156,292	27,458	-26,280						
1442	GC01M154167 (RN7SL431P)		(-)	154,166,246	154,166,549	304	9,955	18	0.35				
1443	LNC-C1orf189-1		(+)	154,179,706	154,180,993	1,288	13,158	19	0.53				
1444	LNC-C1orf189-2		(+)	154,184,933	154,186,992	2,060	**3,941**	**20**					
1445	TPM3		(-)	154,155,304	154,194,648	39,345	-31,687						
1446	C1orf189		(-)	154,199,085	154,206,365	7,281	**4,438**	**21**					
1447	C1orf43		(-)	154,206,706	154,220,628	13,923	**342**	**22**					
1448	LNC-C1orf43-4		(-)	154,232,738	154,238,979	6,242	**12,111**	**23**					
1449	LNC-SHE-3		(-)	154,349,442	154,351,749	2,308	110,464	24	0.073				
1450	LNC-SHE-2		(-)	154,374,804	154,379,273	2,308	23,056	25	*0.76*				
1451	MRPS33P1		(-)	154,377,852	154,378,218	367							
1452	RPSAP17		(-)	154,378,126	154,379,169	1,044							
1453	LOC101928101		(-)	154,402,321	154,408,834	6,514	23,049	26	0.35				
1454	PSMD8P1		(-)	154,414,272	154,415,222	951	**5,439**	**27**					
1455	LNC-SHE-1		(-)	154,442,113	154,445,591	3,479	26,892	28	0.80				
1456	SHE		(-)	154,469,637	154,502,113	32,477	24,047	29	0.53				
1457	LNC-ADAR-2		(-)	154,522,885	154,525,552	2,668	20,773	30	0.68				
1458	LNC-UBE2Q1-1		(-)	154,533,953	154,540,253	6,301	**8,402**	**31**					
1459	UBE2Q1		(-)	154,548,575	154,559,028	10,454	**8,323**	**32**					
1460	LNC-ADAR-3		(-)	154,574,554	154,600,475	25,922	15,527	33	0.54				
1461	ADAR		(-)	154,582,057	154,628,013	45,957	-18,417						
1462	LNC-KCNN3-2		(-)	154,671,125	154,675,050	3,926	43,113	34	0.70				
1463	LNC-KCNN3-1		(-)	154,675,205	154,680,702	5,498	**156**	**35**					
1464	LNC-ADAR-4		(-)	154,682,828	154,692,882	10,055	**2,127**	**36**					
1465	KCNN3		(-)	154,697,455	154,870,280	172,826	4,574	37	0.28				
1466	LNC-PMVK-8		(-)	154,874,765	154,874,969	205	**4,486**	**38**					
1467	LNC-PMVK-7		(-)	154,875,959	154,876,532	574	**991**	**39**					

1468	LNC-PMVK-6	(-)	154,877,513	154,877,872	360	**982**	**40**		
1469	LNC-PMVK-5	(-)	154,877,982	154,878,314	333	**111**	**41**		
1470	LNC-PMVK-4	(-)	154,878,566	154,878,768	203	**253**	**42**		
1471	LNC-PMVK-3/LNC-PMVK-2	(-)	154,880,039	154,883,596	3,558	**1,272**	**43**		
1472	LNC-PMVK-1	(-)	154,918,421	154,918,723	303	34,826	44	0.38	
1473	LOC105371449/LNC-PYGO2-2	(-)	154,919,506	154,928,413	8,908	**784**	**45**		
1474	PMVK/LNC-PYGO2-1	(-)	154,924,732	154,938,238	13,507	-3,680			
1475	PBXIP1	(-)	154,944,076	154,956,123	12,048	**5,839**	**46**		
1476	PYGO2	(-)	154,957,026	154,963,853	6,828	**904**	**47**		
1477	SHC1	(-)	154,962,298	154,974,395	12,098	-1,554			
1478	GC01M154997	(-)	154,997,267	155,000,640	3,374	**22,873**	**48**		
1479	DCST2	(-)	155,017,448	155,033,781	16,334	16,809	49	0.66	
1480	LNC-DCST2-1	(-)	155,017,667	155,036,467	18,801	-16,113			
1481	LOC100505666	(-)	155,045,191	155,063,991	18,801	**8,725**	**50**		
1482	DPM3	(-)	155,139,891	155,140,595	705	75,901	51	0.10	
1483	LNC-KRTCAP2-1	(-)	155,141,884	155,159,747	17,864	**1,290**	**52**		

1484	MTHFD2P5	(-)	82,588,770	82,590,691	1,922	**100,029**	**30**	**0.06**	
1485	LOC105375376	(-)	82,690,719	82,695,876	5,158	58,130	29		
1486	PCLO	(-)	82,754,005	83,162,930	408,926	5,226	28		
1487	LOC105375377	(-)	83,168,155	83,175,460	7,306	188,447	27		
1488	SEMA3E	(-)	83,363,906	83,649,163	285,258	135,054	26		
1489	GC07M083784	(-)	83,784,216	83,822,383	38,168	**97,220**	**25**	**0.27**	**19**
1490	ENSG00000222994	(-)	83,919,602	83,919,703	102	15,996	24		
1491	LNC-SEMA3D-5	(-)	83,935,698	84,001,607	65,910	-45,829			
1492	SEMA3A/LNC-SEMA3D-6	(-)	83,955,777	84,509,941	554,165	**18,177**	**23**	**0.53**	**18**
1493	RPL7P30	(-)	84,528,117	84,528,830	714	**39,833**	**22**	**0.09**	**17**
1494	LNC-SEMA3D-1	(-)	84,568,662	84,569,561	900	**78,976**	**21**	***0.87***	**16**
1495	LOC105375379	(-)	84,648,536	84,880,517	231,982	**14,966**	**20**	**0.25**	**15**
1496	GC07M084895	(-)	84,895,482	84,910,552	15,071	28,784	19		
1497	ENSG00000280325	(-)	84,939,335	84,940,256	922	**54,937**	**18**	***0.89***	**14**
1498	SEMA3D	(-)	84,995,192	85,186,855	191,664	**78,475**	**17**	**0.26**	**13**
1499	LNC-SEMA3D-4	(-)	85,265,329	85,265,660	332	**115,446**	**16**	**0.15**	**12**
1500	DYNLL1P7	(-)	85,381,105	85,381,471	367	**254,543**	**15**	**0.03**	**11**
1501	ENSG00000227785	(-)	85,636,013	85,636,344	332	**136,991**	**14**	**0.0005**	**10**
1502	GC07M085773	(-)	85,773,334	85,818,055	44,722	**28,047**	**13**	**0.72**	**9**
1503	LNC-KIAA1324L-4	(-)	85,846,101	85,847,049	949	**262,178**	**12**	**0.01**	**8**
1504	GC07M086110	(-)	86,109,226	86,123,992	14,767	**92,794**	**11**	**0.26**	**7**
1505	SOCS5P1	(-)	86,216,785	86,217,733	949	**144,032**	**10**	**0.46**	**6**
1506	GC07M086361	(-)	86,361,764	86,453,885	92,122	**66,627**	**9**	**0.11**	**5**
1507	LNC-C7orf23-3	(-)	86,520,511	86,522,251	1,741	**72,089**	**8**	**0.04**	**4**
1508	LOC105375381	(-)	86,594,339	86,614,750	20,412	**158,565**	**7**	**0.10**	**3**
1509	LOC105375382	(-)	86,773,314	86,795,187	21,874	-2,586			
1510	LNC-C7orf23-2	(-)	86,792,600	86,812,767	20,168	13,150	6		
1511	LNC-KIAA1324L-3	(-)	86,825,916	86,848,942	23,027	**105,691**	**5**	**0.58**	**2**
1512	LNC-TP53TG1	(-)	86,954,632	86,974,883	20,252	-97,976			
1513	KIAA1324L	(-)	86,876,906	87,059,699	182,794	**91,725**	**4**	***0.91***	**1**
1514	LOC101927420	(-)	87,151,423	87,156,676	5,254	39,485	3		
1515	TMEM243	(-)	87,196,160	87,220,587	24,428	8,848	2		

1516	LNC-ABCB1-2		(-)	87,229,434	87,342,728	113,295	-17,502						
1517	TP53TG1		(-)	87,325,225	87,345,515	20,291	**56,183**	**1**	***0.81***	**0**			
1518	**ABCB4**		(-)	87,401,697	87,581,152	**179,456**							
**1519w/in**	**ABCB1**	**7q21.12**	(-)	87,503,633	87,713,323	**209,691**					**BMEnC**	**4**	**> 265,005**
1520	**GC07M087666**		(-)	87,666,878	87,787,880	**121,003**							**< 607,463**
1521	SLC25A40		(-)	87,833,568	87,876,377	42,810	**45,689**	**1**					
1522	LOC105375385		(-)	87,888,066	87,926,669	38,604	11,690	2	*0.76*				
1523	GC07M087968		(-)	87,968,505	87,994,939	26,435	41,837	3	*0.94*				
1524	LNC-STEAP4-3		(-)	88,078,103	88,078,332	230	**83,165**	**4**					
1525	LOC105375386		(-)	88,140,085	88,157,816	17,732	**61,754**	**5**					
1526	GC07M088203		(-)	88,183,439	88,186,554	3,116	25,624	6	0.51				
1527	PIR58316		(-)	88,202,251	88,202,280	30	**15,698**	**7**					
1528	SRI		(-)	88,205,115	88,226,993	21,879	**2,836**	**8**					
1529	GC07M088224		(-)	88,224,713	88,263,709	38,997	-2,279						
1530	STEAP4		(-)	88,270,892	88,306,913	36,022	**7,184**	**9**					
1531	GC07M088373		(-)	88,373,253	88,399,418	26,166	**66,341**	**10**					
1532	PIR31447		(-)	88,423,189	88,423,216	28	**23,772**	**11**					
1533	GC07M088426		(-)	88,426,167	88,428,482	2,316	**2,952**	**12**					
1534	EIF4A1P13		(-)	88,448,788	88,449,026	239	**20,307**	**13**					
1535	GC07M088450		(-)	88,449,091	88,449,231	141	**66**	**14**					
1536	GC07M088457		(-)	88,457,975	88,461,795	3,821	**8,745**	**15**					
1537	GC07M088479		(-)	88,479,089	88,483,599	4,511	**17,295**	**16**					
1538	GC07M088503		(-)	88,503,785	88,515,842	12,058	**20,187**	**17**					
1539	LOC645680		(-)	88,564,468	88,566,406	1,939	**48,627**	**18**					
1540	GC07M088598		(-)	88,598,166	88,606,503	8,338	31,761	19	*0.91*				
1541	PQLC1P1		(-)	88,610,241	88,610,669	429	**3,739**	**20**					
1542	GC07M088679		(-)	88,679,260	88,680,136	877	**68,592**	**21**					
1543	GC07M088683		(-)	88,683,690	88,684,347	658	**3,555**	**22**					
1544	GC07M088694		(-)	88,694,060	88,695,468	1,409	**9,714**	**23**					
1545	GC07M088728		(-)	88,728,954	88,729,985	1,032	33,487	24	0.48				
1546	GC07M088756		(-)	88,756,666	88,801,445	44,780	**26,682**	**25**					
1547	GC07M088936		(-)	88,936,854	89,019,536	82,683	135,410	26	0.997				
1548	GC07M089217		(-)	89,217,066	89,236,264	19,199	197,531	27	0.95				
1549	GC07M089301		(-)	89,301,704	89,303,435	1,732	65,441	28	0.08				
1550	GC07M089581		(-)	89,581,498	89,583,473	1,976	278,064	29	0.21				
1551	GC07M089589		(-)	89,589,388	89,590,401	1,014	**5,916**	**30**					

1552	GC07P111351		(+)	111,351,442	111,354,792	3,351	**40,084**	**39**	
1553	ENSG00000243621		(+)	111,394,875	111,395,043	169	**16,277**	**38**	
1554	LOC100420226		(+)	111,411,319	111,411,883	565	**36,679**	**37**	
1555	LNC-DOCK4-AS1		(+)	111,448,561	111,461,835	13,275	179,838	36	0.04
1556	GC07P111641		(+)	111,641,672	111,656,837	15,166	22,642	35	0.47
1557	GC07P111679		(+)	111,679,478	111,680,597	1,120	**2,288**	**34**	
1558	GC07P111682		(+)	111,682,884	111,684,113	1,230	**72,862**	**33**	
1559	LOC105375455		(+)	111,756,974	111,758,372	1,399	**50,134**	**32**	
1560	DOCK4-AS1		(+)	111,808,505	111,821,779	13,275	18,916	31	0.38
1561	LOC105375454		(+)	111,840,694	111,846,992	6,299	81,687	30	0.27
1562	LNC-IFRD1-2		(+)	111,928,678	111,929,007	330	83,696	29	0.27
1563	LNC-ZNF277-2		(+)	112,012,702	112,013,784	1,083	**596**	**28**	

1564	LNC-ZNF277-3		(+)	112,014,379	112,015,484	1,106	**9,432**	**27**					
1565	GC07P112024		(+)	112,024,915	112,039,193	14,279	**6,152**	**26**					
1566	LNC-IFRD1-1		(+)	112,045,344	112,047,935	2,592	49,117	25	0.47				
1567	LNC-C7orf53-4		(+)	112,097,051	112,108,159	11,109	98,430	24	0.10				
1568	ZNF277/LNC-C7orf53-3		(+)	112,206,588	112,348,135	141,548	**24,513**	**23**					
1569	RN7SKP187		(+)	112,288,623	112,288,952	330							
1570	MTND5P8		(+)	112,372,647	112,373,729	1,083	**596**	**22**					
1571	MTCYBP24		(+)	112,374,324	112,375,429	1,106	26,811	21	0.52				
1572	LNC-C7orf53-5		(+)	112,402,239	112,402,812	574	**20,157**	**20**					
1573	IFRD1		(+)	112,422,968	112,481,017	58,050	-778						
1574	LSMEM1		(+)	112,480,238	112,491,062	10,825	**29,335**	**19**					
1575	NPM1P14		(+)	112,520,396	112,521,983	1,588	**292**	**18**					
1576	LOC105375458		(+)	112,522,274	112,617,338	95,065	**728**	**17**					
1577	LOC100996249		(+)	112,618,065	112,620,649	2,585	1,730	16	0.31				
1578	LOC101928012		(+)	112,622,378	112,708,080	85,703	15,205	15	0.41				
1579	LNC-AC073348.1-2		(+)	112,723,284	112,727,810	4,527	**548**	**14**					
1580	GC07P112728		(+)	112,728,357	112,731,015	2,659	**9,704**	**13**					
1581	LNC-AC073348.1-1		(+)	112,740,718	112,786,385	45,668	269,284	12	0.14				
1582	LOC105375460		(+)	113,055,668	113,061,751	6,084	**10,041**	**11**					
1583	GC07P113071		(+)	113,071,791	113,074,986	3,196	**25,678**	**10**					
1584	ENSG00000225457		(+)	113,100,663	113,146,330	45,668	199,141	9	0.28				
1585	ENSG00000277061		(+)	113,345,470	113,345,580	111	70,110	8					
1586	ENSG00000270997		(+)	113,415,689	113,416,322	634	34,751	7	0.49				
1587	ENSG00000278894		(+)	113,451,072	113,451,402	331	**12,042**	**6**					
1588	GC07P113463		(+)	113,463,443	113,476,672	13,230	100,088	5	0.18				
1589	PIR47966		(+)	113,576,759	113,576,788	30	**71,034**	**4**					
1590	GC07P113647		(+)	113,647,821	113,669,665	21,845	50,863	3	0.18				
1591	GC07P113720		(+)	113,720,527	113,723,349	2,823	**4,354**	**2**					
1592	LNC-MDFIC-7		(+)	113,727,702	114,066,565	338,864	19,746	1	*0.85*				
**1593**	**FOXP2**	**7q31.1**	(+)	114,086,310	114,693,772	**607,463**					**BMEnC**	**5**	**≥ 607,463**
1594	GC07P114710		(+)	114,710,534	114,710,734	201	16,763	1					**< 2,241,933**
1595	LNC-LINC01393		(+)	114,719,012	114,766,368	47,357	**8,279**	**2**	**0.53**	**0**			
1596	GC07P114775		(+)	114,775,609	114,789,243	13,635	9,242	3					
1597	LNC-MDFIC-3		(+)	114,903,933	114,904,197	265	**114,691**	**4**	**0.62**	**1**			
1598	LNC-MDFIC-4/MDFIC		(+)	114,921,881	115,019,916	98,036	17,685	5					
1599	LINCO1393		(+)	115,078,958	115,126,314	47,357	**59,043**	**6**	**0.20**	**2**			
1600	LNC-MDFIC-5		(+)	115,143,421	115,144,135	715	17,108	7					
1601	GC07P115309		(+)	115,309,520	115,313,204	3,685	**165,386**	**8**	**0.42**	**3**			
1602	LNC-TES-3		(+)	115,369,353	115,369,576	224	56,150	9					
1603	LNC-TES-1		(+)	115,429,783	115,431,383	1,601	**60,208**	**10**	**0.43**	**4**			
1604	GC07P115459		(+)	115,459,780	115,465,699	5,920	**28,398**	**11**	**0.35**	**5**			
1605	ENSG00000244565		(+)	115,503,367	115,504,081	715	37,669	12					
1606	GC07P115639		(+)	115,639,396	115,669,481	30,086	**135,316**	**13**	**0.07**	**6**			
1607	GC07P115673		(+)	115,673,868	115,674,130	263	**4,388**	**14**	**0.12**	**7**			
1608	LNC-TES-2		(+)	115,680,406	115,682,254	1,849	6,277	15					
1609	LOC105375461		(+)	115,682,791	115,684,594	1,804	538	16					
1610	GC07P115721/GC07P115722		(+)	115,721,523	115,721,881	359	**36,930**	**17**	**0.02**	**8**			
1611	LOC102724407		(+)	115,789,729	115,791,323	1,595	**67,849**	**18**	**0.004**	**9**			
1612	LNC-CAV2-2		(+)	115,893,170	115,895,118	1,949	**101,848**	**19**	**0.29**	**10**			
1613	LNC-CAV2-1		(+)	115,927,434	116,146,162	218,729	**32,317**	**20**	**0.62**	**11**			

1614	LNC-CAV1	(+)	116,160,067	116,160,303	237	13,906	21		
1615	TES	(+)	116,210,493	116,258,786	48,294	**50,191**	**22**	**0.01**	**12**
1616	LOC105375463/CAV2/LNC-ST7-4	(+)	116,285,341	116,544,390	259,050	**26,556**	**23**	***0.92***	
1617	CAV1	(+)	116,524,785	116,561,185	36,401	-19,604			
1618	LNC-CAPZA2-1	(+)	116,570,565	116,570,828	264	9,381	24		
1619	LNC-ST7-OT4-2	(+)	116,593,953	116,738,860	144,908	23,126	25		
1620	MET	(+)	116,672,390	116,798,386	125,997	-66,469			
1621	CAPZA2	(+)	116,811,070	116,922,049	110,980	**12,685**	**26**	**0.48**	**13**
1622	LOC105375465/LNC-ST7-1	(+)	116,938,519	116,962,290	23,772	**16,471**	**27**	**0.57**	**14**
1623	ST7/ST7-OT3	(+)	116,953,238	117,230,103	276,866	-9,051			
1624	LOC105375466	(+)	117,232,821	117,241,161	8,341	**2,719**	**28**	**0.22**	**15**
1625	MTND4P6	(+)	117,263,895	117,264,394	500	22,735	29		
1626	LOC105375467	(+)	117,286,797	117,322,563	35,767	22,404	30		
1627	LNC-CFTR-1	(+)	117,329,767	117,356,025	26,259	7,205	31		
1628	LOC105375468	(+)	117,447,916	117,475,729	27,814	**91,892**	**32**	**0.55**	**16**
1629	CFTR	(+)	117,465,784	117,715,971	250,188	-9,944			
1630	LOC105375469	(+)	117,715,739	117,730,525	14,787	-231			
1631	LNC-ANKRD7-1	(+)	117,854,727	117,882,769	28,043	**124,203**	**33**	**0.59**	**17**
1632	LOC101929527	(+)	117,873,680	117,883,723	10,044	-9,088			
1633	LSM8	(+)	118,184,032	118,204,039	20,008	**300,310**	**34**	**0.01**	**18**
1634	ANKRD7	(+)	118,214,669	118,496,171	281,503	10,631	35		
1635	LOC105375473	(+)	118,503,018	118,545,686	42,669	6,848	36		
1636	LOC105375474	(+)	118,859,070	118,862,193	3,124	**313,385**	**37**	**0.12**	**19**
1637	LNC-KCND2-3	(+)	119,259,423	119,264,891	5,469	**397,231**	**38**	**0.04**	**20**
1638	LNC-KCND2-2	(+)	119,344,514	119,348,369	3,856	**79,624**	**39**	**0.50**	

1639	LOC38942	(-)	25,645,592	25,646,439	848	**177,317**	**53**	**0.62**	
1640	GC0XM025823	(-)	25,823,755	25,864,238	40,484	**225,150**	**52**	**0.09**	**26**
1641	GC0XM026089	(-)	26,089,387	26,098,435	9,049	**101,262**	**51**	**0.50**	**25**
1642	PIR62809	(-)	26,199,696	26,199,722	27	129,096	50		
1643	GC0XM026328	(-)	26,328,817	26,338,284	9,468	**53,647**	**49**	**0.18**	**24**
1644	LOC105373149	(-)	26,391,930	26,410,110	18,181	**109,356**	**48**	**0.37**	**23**
1645	GC0XM026519	(-)	26,519,465	26,519,905	441	**30,311**	**47**	**0.17**	
1646	GC0XM026550	(-)	26,550,215	26,587,620	37,406	**69,672**	**46**	**0.57**	
1647	ENSG00000236160	(-)	26,657,291	26,657,507	217	**17,902**	**45**	**0.23**	**20**
1648	LNC-DCAF8L1-9	(-)	26,675,408	26,675,624	217	**117,602**	**44**	**0.08**	**19**
1649	GC0XM026793	(-)	26,793,225	26,825,626	32,402	**217,282**	**43**	**0.02**	**18**
1650	ENSG00000242021	(-)	27,042,907	27,176,298	133,392	-115,273			
1651	LNC-DCAF8L1-2	(-)	27,061,024	27,417,114	356,091	10,608	42		
1652	PTP4AIP5	(-)	27,427,721	27,428,231	511	**17,664**	**41**	**0.03**	**17**
1653	LNC-DCAF8L1-7	(-)	27,445,894	27,446,348	455	3,374	40		
1654	GC0XM027449	(-)	27,449,721	27,450,639	919	**9,573**	**39**	**0.09**	**16**
1655	PPP4R3CP	(-)	27,460,211	27,463,341	3,131	51,831	38		
1656	RDXP2	(-)	27,515,171	27,517,883	2,713	**225,869**	**37**	**0.20**	**15**
1657	ENSG00000263522	(-)	27,743,751	27,743,848	98	**103,741**	**36**	**0.07**	**14**
1658	ENSG000000232834	(-)	27,847,588	27,847,939	352	17,767	35		
1659	LNC-DCAF8L1-4	(-)	27,865,705	27,866,056	352	27,293	34		
1660	LOC340569	(-)	27,893,348	27,894,059	712	**17,407**	**33**	***0.81***	**13**
1661	LNC-DCAF8L1-3	(-)	27,911,465	27,912,176	712	11,310	32		

1662	GC0XM027923		(-)	27,923,485	27,963,854	40,370	14,140	31					
1663	DCAF8L1		(-)	27,977,993	27,981,449	3,457	**76,827**	**30**	**0.47**	**12**			
1664	GC0XM028058		(-)	28,058,275	28,065,342	7,068	**311,622**	**29**	**0.01**	**11**			
1665	ENSG00000263509		(-)	28,376,963	28,377,061	99	**118,495**	**28**	**0.24**	**10**			
1666	MIR6134		(-)	28,495,555	28,495,663	109	3,908	27					
1667	LNC-DCAF8L1-5		(-)	28,499,570	28,514,433	14,864	57,100	26					
1668	ENSG00000223742		(-)	28,571,532	28,586,395	14,864	3,255	25					
1669	LNC-DCAF8L1-1		(-)	28,589,649	28,604,512	14,864	**54,554**	**24**	**0.64**	**9**			
1670	LOC100506811		(-)	28,659,065	28,659,469	405	**323,384**	**23**	**0.27**	**8**			
1671	RNA5SP500		(-)	28,982,852	28,982,970	119	**386,032**	**22**	***0.83***	**7**			
1672	ENSG00000227393		(-)	29,369,001	29,369,845	845	17,274	21					
1673	LNC-NR0B1-3		(-)	29,387,118	29,387,962	845	**360,852**	**20**	**0.16**	**6**			
1674	GC0XM029748		(-)	29,748,813	29,752,713	3,901	**161,166**	**19**	**0.14**	**5**			
1675	GC0XM029913		(-)	29,913,878	29,914,243	366	**17,753**	**18**	**0.11**	**4**			
1676	LNC-NR0B1-2		(-)	29,931,995	29,932,360	366	**84,801**	**17**	**0.25**	**3**			
1677	PIGFP3		(-)	30,017,160	30,017,881	722	**17,397**	**16**	***0.90***	**2**			
1678	LNC-NR0B1-1		(-)	30,035,277	30,035,998	722	253,856	15					
1679	PLCE1P1		(-)	30,289,853	30,290,358	506	13,849	14					
1680	NR0B1		(-)	30,304,206	30,309,598	5,393	5,488	13					
1681	ENSG00000266257		(-)	30,315,085	30,315,178	94	167,442	12					
1682	GC0XM030482		(-)	30,482,619	30,488,035	5,417	31,025	11					
1683	GC0XM030519		(-)	30,519,059	30,527,717	8,659	31,108	10					
1684	CXorf21		(-)	30,558,824	30,577,916	19,093	70,359	9					
1685	LNC-CXorf21-1		(-)	30,648,274	30,648,587	314	12,941	8					
1686	GC0XM030661		(-)	30,661,527	30,675,365	13,839	**24,634**	**7**	**0.71**	**1**			
1687	GK-AS1/LNC-GK-AS1		(-)	30,699,998	30,742,291	42,294	20,968	6					
1688	LNC-TAB3-2		(-)	30,763,258	30,768,259	5,002	29,795	5					
1689	GC0XM030798		(-)	30,798,053	30,815,640	17,588	6,176	4					
1690	LOC100418759		(-)	30,821,815	30,823,432	1,618	4,011	3					
1691	TAB3		(-)	30,827,442	30,975,084	147,643	-122						
1692	GC0XM030974		(-)	30,974,961	30,989,833	14,873	**81,409**	**2**	**0.10**	**0**			
1693	FTHL17		(-)	31,071,241	31,072,053	813	25,625	1					
**1694**	**DMD**	**Xp21.1**	(-)	31,097,677	33,339,609	**2,241,933**					**BMEnC**	**6**	**≥ 2,241,933**
1695	GC0XM033707		(-)	33,707,437	33,714,935	7,499	367,829	1	0.07				
1696	GC0XM033723		(-)	33,723,233	33,729,484	6,252	**8,299**	**2**					
1697	FAM47A		(-)	34,129,752	34,132,330	2,579	400,269	3	0.01				
1698	ENSG00000233571/LNC-FAM47A-1		(-)	34,206,725	34,433,654	226,930	74,396	4	0.08				
1699	TMEM47		(-)	34,627,064	34,657,288	30,225	193,411	5	0.15				
1700	ENSG00000225384		(-)	34,717,440	34,717,992	553	60,153	6	0.35				
1701	LNC-TMEM47-1		(-)	34,735,557	34,736,109	553	**17,566**	**7**					
1702	ENSG00000273704		(-)	34,768,327	34,768,688	362	32,219	8	0.40				
1703	PIR51814		(-)	34,990,153	34,990,185	33	221,466	9	0.32				
1704	ENSG00000222864		(-)	35,056,331	35,056,458	128	66,147	10	0.47				
1705	GC0XM035122		(-)	35,122,823	35,127,861	5,039	66,366	11	0.47				
1706	GC0XM035958		(-)	35,958,517	36,092,964	134,448	830,657	12	0.20				
1707	RNU6-641P		(-)	36,021,939	36,022,045	107							
1708	LOC101928627/LNC-DYNLT3-1		(-)	36,365,626	36,458,375	92,750	272,663	13	0.02				
1709	RPS15AP40		(-)	36,374,755	36,375,213	459							
1710	GC0XM036497		(-)	36,496,931	36,498,771	1,841	38,557	14	0.36				
1711	PIR50996		(-)	36,778,807	36,778,835	29	280,037	15	0.91	TC			

1712	ENSG00000275314	(-)	36,794,500	36,795,460	961	**15,666**	**16**	
1713	LOC105373154	(-)	36,816,330	36,959,103	142,774	**20,871**	**17**	
1714	LOC441488	(-)	36,961,001	36,963,558	2,558	**1,899**	**18**	
1715	LOC100422408	(-)	36,985,346	36,986,093	748	**21,789**	**19**	
1716	FTH1P18	(-)	37,042,882	37,043,794	913	**56,790**	**20**	
1717	LOC442445	(-)	37,077,851	37,078,401	551	34,058	21	0.51
1718	LNC-DYNLT3-2	(-)	37,400,330	37,404,515	4,186	**321,930**	**22**	
1719	GC0XM037490	(-)	37,490,445	37,505,819	15,375	**85,931**	**23**	
1720	FAM47DP	(-)	37,540,929	37,545,262	4,334	**35,111**	**24**	
1721	GC0XM037554	(-)	37,554,271	37,557,232	2,962	9,010	25	0.36
1722	DYNLT3	(-)	37,836,757	37,847,637	10,881	279,526	26	0.20
1723	GC0XM037971/GC0XM037972	(-)	37,971,389	37,980,003	8,615	123,753	27	0.03
1724	LNC-RPGR-4	(-)	38,008,604	38,019,351	10,748	**28,602**	**28**	
1725	MIR548AJ2	(-)	38,023,895	38,023,986	92	**4,545**	**29**	
1726	GC0XM038060/GC0XM038061	(-)	38,060,093	38,072,878	12,786	**36,108**	**30**	
1727	SRPX	(-)	38,149,335	38,220,924	71,590	76,458	31	0.18
1728	GC0XM038216	(-)	38,216,119	38,232,939	16,821	-4,804		
1729	LNC-RPGR-3	(-)	38,254,622	38,255,148	527	21,684	32	0.52
1730	RPGR	(-)	38,269,163	38,327,564	58,402	**14,016**	**33**	
1731	TDGF1P1	(-)	38,395,369	38,395,895	527	67,806	34	0.40
1732	PIR55505	(-)	38,477,830	38,477,856	27	81,936	35	0.22
1733	FTLP16	(-)	38,484,752	38,485,264	513	**6,897**	**36**	
1734	UBTFL11	(-)	38,531,408	38,534,256	2,849	**46,145**	**37**	
1735	LNC-RPGR-1	(-)	38,639,280	38,659,679	20,400	105,025	38	0.49
1736	LNC-MID11P1-AS1	(-)	38,660,501	38,663,136	2,636	**823**	**39**	
1737	LOC105373174	(-)	38,779,501	38,800,658	21,158	116,366	40	0.03
1738	MID1IP1-AS1	(-)	38,801,248	38,803,883	2,636	**591**	**41**	
1739	LNC-RPGR-5	(-)	38,970,300	39,043,984	73,685	166,418	42	0.06
1740	LNC-BCOR-11	(-)	39,049,154	39,071,560	22,407	**5,171**	**43**	
1741	RNU6-591P	(-)	39,081,180	39,081,285	106	**9,621**	**44**	
1742	LNC-BCOR-5/LNC-BCOR-9	(-)	39,085,356	39,179,022	93,667	**4,072**	**45**	
1743	LNC-LINC01281	(-)	39,164,210	39,186,616	22,407	-14,811		
1744	LOC105373175	(-)	39,226,103	39,299,786	73,684	**39,488**	**46**	
1745	LINC01281	(-)	39,304,956	39,327,362	22,407	**5,171**	**47**	
1746	LINC01282	(-)	39,367,285	39,391,774	24,490	**39,924**	**48**	
1747	ENSG00000234191	(-)	39,401,252	39,434,824	33,573	**9,479**	**49**	
1748	LNC-BCOR-8	(-)	39,645,780	39,646,077	298	210,957	50	0.61
1749	LNC-BCOR-1	(-)	39,696,790	39,707,612	10,823	**50,714**	**51**	
1750	LOC105373179	(-)	39,726,792	39,849,655	122,864	**19,181**	**52**	
1751	RN7SL732P	(-)	39,786,524	39,786,823	300			
1752	GAPDHP1	(-)	39,786,931	39,788,136	1,206			
1753	LNC-CXorf38-6	(-)	39,958,945	39,962,809	3,865	**109,291**	**53**	

Table S2 Legend. OE, overexpression

(1) **NC, Not Considered (bold red)**

(2) TC, Tempered Considered (light red)

(3) Pseudogene (orange)

(4) *3′ -> 5′ reverse stabilizing isotropy (stIsotropy) ≥ 0.75* (*black italics*)

(5) Preceding 3'-> 5' reverse anisotropy $prpT_Q$s ($prpT_Q$s < 0.25) of equivalent or greater magnitude to result in not considered (NC) following 5'->3' $prpT_Q$ (green underlined)

(6) ≤ 15 base intergene distances, black underlined; ≤ 15 base intergene distances are considered overlap, which in series would be ~7.5 nm in total van der Waals diameter (vdWD) as each base has a van der Waals diameter of ~0.5 nm (thus, > 15 base intergene distance could be considered as sufficient weighted pressuromodulation-sensitive intergene distance)
