# Supplementary material for: Horizontal alignment of 5′ -> 3′ intergene distance segment tropy with respect to the gene as the conserved basis for DNA transcription
Source: Future Sci OA. 2016 Dec 2;3(1):FSO160. doi: 10.4155/fsoa-2016-0070 (PMC5351715; doi:10.4155/fsoa-2016-0070)
Supplement: Supplementary file 3 [file fsoa-03-160-s3.doc]

**Supplementary file 3 - Table S3. Sequential episodic sub-episode block sums split-integrated weighted average-averaged gene overexpression tropy quotients (*esebssiwaagoT*_Q_s) to final *esebssiwaagoT*_Q_**

| Gene Symbol | No. of Episodes  [No. of Final SEBs] |  |  |  |  |  |  |  |  |  |  |  |  |  |
| --- | --- | --- | --- | --- | --- | --- | --- | --- | --- | --- | --- | --- | --- | --- |
| SORL1 | 2  [5(-2): 3] | 0.51 | 0.26 | **0.29** | n/a | n/a | n/a | n/a | n/a | n/a | n/a | n/a | n/a | n/a |
| PDPN | 2  (5) | 0.55 | 0.41 | 0.48 | 0.44 | **0.41** | n/a | n/a | n/a | n/a | n/a | n/a | n/a | n/a |
| BTG1 | 2  (5) | 0.05 | 0.35 | 0.30 | 0.25 | **0.25** | n/a | n/a | n/a | n/a | n/a | n/a | n/a | n/a |
| HAPLN1 | 2  [5(+2): 7] | 0.50 | 0.40 | 0.36 | 0.33 | 0.36 | 0.36 | **0.35** | n/a | n/a | n/a | n/a | n/a | n/a |
| MRC1 | 2  [5(+2): 7] | 0.16 | 0.22 | 0.24 | 0.28 | 0.29 | 0.26 | **0.28** | n/a | n/a | n/a | n/a | n/a | n/a |
| ACPP | 2  [5(-2): 3] | 0.39 | 0.17 | **0.25** | n/a | n/a | n/a | n/a | n/a | n/a | n/a | n/a | n/a | n/a |
| TGFA | 2  (5) | 0.35 | 0.24 | 0.32 | 0.31 | **0.31** | n/a | n/a | n/a | n/a | n/a | n/a | n/a | n/a |
| PHLPP1 | 2  (5) | 0.51 | 0.48 | 0.50 | 0.38 | **0.35** | n/a | n/a | n/a | n/a | n/a | n/a | n/a | n/a |
| SELE | 2  [5(+2): 7] | 0.07 | 0.35 | 0.28 | 0.25 | 0.25 | 0.24 | **0.20** | n/a | n/a | n/a | n/a | n/a | n/a |
| CDH11 | 2  (5) | 0.36 | 0.29 | 0.38 | 0.12 | **0.12** | n/a | n/a | n/a | n/a | n/a | n/a | n/a | n/a |
| ZCCHC2 | 2  [5(+2): 7] | 0.16 | 0.26 | 0.27 | 0.29 | 0.28 | 0.28 | **0.17** | n/a | n/a | n/a | n/a | n/a | n/a |
| S100A2 | 3  (7) | 0.08 | 0.42 | 0.45 | 0.39 | 0.31 | 0.30 | **0.31** | n/a | n/a | n/a | n/a | n/a | n/a |
| PRR3 | 3  [7(+2): 9] | 0.15 | 0.26 | 0.26 | 0.27 | 0.27 | 0.33 | 0.24 | 0.26 | **0.24** | n/a | n/a | n/a | n/a |
| IFI27 | 3  (7) | 0.03 | 0.30 | 0.26 | 0.30 | 0.25 | 0.23 | **0.23** | n/a | n/a | n/a | n/a | n/a | n/a |
| S100A14 | 3  (7) | 0.49 | 0.44 | 0.42 | 0.28 | 0.25 | 0.13 | **0.12** | n/a | n/a | n/a | n/a | n/a | n/a |
| ABCB1 | 4  [9(-2): 7] | 0.69 | 0.29 | 0.25 | 0.24 | 0.21 | 0.18 | **0.18** | n/a | n/a | n/a | n/a | n/a | n/a |
| FOXP2 | 5  (11) | 0.63 | 0.50 | 0.42 | 0.31 | 0.28 | 0.30 | 0.28 | 0.31 | 0.34 | 0.21 | **0.21** | n/a | n/a |
| DMD | 6  (13) | 0.10 | 0.37 | 0.27 | 0.30 | 0.27 | 0.25 | 0.23 | 0.22 | 0.23 | 0.22 | 0.24 | 0.23 | **0.23** |
